# Fluorinated Aliphatic Thiols in the Design of Cephalosporin Conjugates for Metallo-β-Lactamase Inhibition

**DOI:** 10.1021/acsomega.6c07917

**Published:** 2026-09-30

**Authors:** Alexandra Kondratieva, Philip Rainsford, Perwez Bakht, Ranjana Pathania, Annette Bayer

**Affiliations:** † Department of Chemistry, UiT The Arctic University of Norway, NO-9037 Tromsø, Norway; ‡ Section for Molecular and Translational Pharmacology, Department of Biomedical Sciences, 4321 University of Copenhagen, 2200 Copenhagen, Denmark; § Department of Biosciences and Bioengineering, 30112 Indian Institute of Technology, Roorkee, Uttarakhand 247667, India

## Abstract

Antibiotic resistance is a major global health challenge, with the rapid spread of metallo-β-lactamase (MBL)-expressing bacteria presenting a serious clinical threat. In this study, we synthesized and investigated a series of novel cephalosporin conjugates envisioned to release MBL inhibitors upon MBL-mediated hydrolysis. The designed compounds consist of a cephalosporin core linked to trifluoromethylated aliphatic thiol-based MBL inhibitors. Conjugates releasing aliphatic thiols upon hydrolysis by the New Delhi Metallo-β-lactamase-1 (NDM-1) were identified, confirming that fluorination improves the leaving group properties of some aliphatic thiols compared to a nonfluorinated control. However, when comparing with a conjugate containing an aromatic thiol, fluorinated aliphatic thiol release appeared slower and mechanistically more complex, thus incompletely resolved. The conjugates exhibited potent inhibitory activity against several subclass B1 MBLs. In addition, the utility of ^19^F NMR as an alternative method for hydrolysis monitoring was exemplified. The findings highlight the potential, but also some unsolved challenges, of altering the leaving group ability of aliphatic thiol-based inhibitors to exploit the catalytic activity of NDM-1 for the selective release of inhibitors in MBL-producing bacteria.

## Graphical abstract

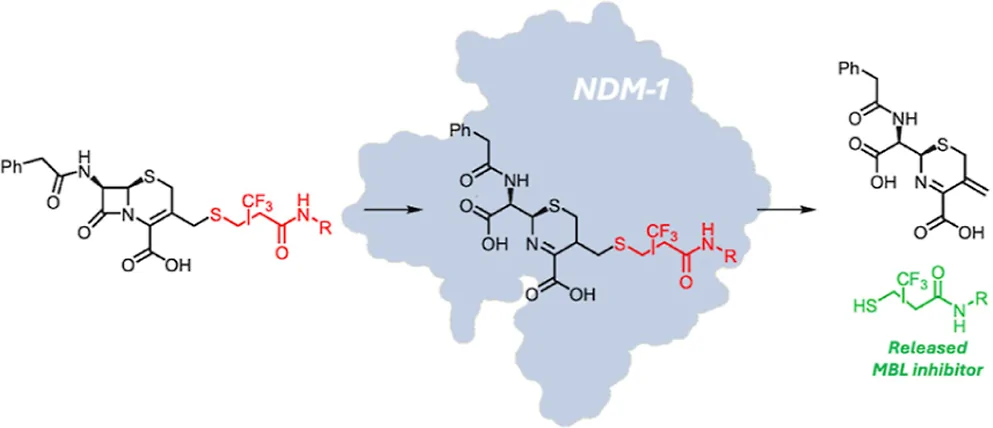

## 1 Introduction

The use of β-lactams, the most widely used class of antibiotics, is continuously compromised by the development of resistance mechanisms in bacteria.^1^ Expression of β-lactamases, enzymes capable of hydrolyzing the β-lactam ring, is of particular concern. Metallo-β-lactamases (MBLs) are capable of inactivating the majority of β-lactams, and there are currently no approved drugs targeting these enzymes.^2–4^ Hydrolysis of the β-lactam ring, mediated by MBLs, occurs through a water molecule activated by one or two zinc ions present in the active site.^5^

A promising strategy to combat resistance caused by MBLs is the development of β-lactamase inhibitors. These inhibitors predominantly act via coordination to, or removal of, the zinc ions in the active site. Many structurally diverse compound classes, such as sulfones, carboxylates, diazabicyclooctanones, boronates, and thiols, have been described.^6,7^ Thiols, more specifically aliphatic thiols, being among the most investigated MBL-inhibiting compound classes, have gained attention due to their broad-spectrum inhibition of all three MBL subclasses.^8,9^

A major limitation of thiol-based MBL inhibitors is their tendency to form disulfides, resulting in the loss of zinc binding ability.^10^ Due to this stability issue, protecting the thiol moiety with a scaffold that is removable under certain conditions is of great interest. Furthermore, using zinc-binding molecules in a complex biological environment could inevitably lead to selectivity issues, as metalloproteins are estimated to constitute a substantial part of the human proteome.^11^ Controllable release of an inhibitor, only in the presence of the target MBL, would limit off-target effects, while the β-lactam-like structure of the conjugate could improve bacterial uptake.

β-Lactamase-mediated hydrolysis of the β-lactam ring has been exploited in the design of diagnostic probes and bioactive conjugates, including conjugates with inhibitory activity toward MBLs. Conjugation of MBL-inhibiting moieties such as ebselen or zinc chelators to a β-lactam via nonhydrolyzable linkers has resulted in promising MBL inhibitors.^12,13^ Fragmenting conjugates are designed based on the principle that β-lactamase-mediated cleavage of the β-lactam ring, followed by spontaneous fragmentation, leads to the release of a reporter molecule or a bioactive species. Various fragmenting β-lactam conjugates have been designed for application in diagnostics, imaging, and as co- and pro-drugs.^14^ Bioactive fragmenting conjugates reported in literature predominantly consist of a cephalosporin core linked to an antibiotic, siderophore, membrane permeabilizer, or inhibitor.^14^ A selection of cephalosporin-based conjugates designed to release thiol inhibitors have been described in literature.^15–19^

Hydrolysis of β-lactams has been thoroughly investigated, and it has been established that hydrolysis of the β-lactam ring, in the presence of a suitable leaving group, leads to release of the attached moiety and the formation of a vinylic elimination product **V** (Figure 1, top). While aromatic thiols are readily released upon enzymatic hydrolysis, aliphatic thiol release remains a problem. Liberation of the inhibitor, in the latter case, has been shown to happen slowly or not at all due to the poor leaving group ability of the aliphatic thiols.

**Figure 1:**
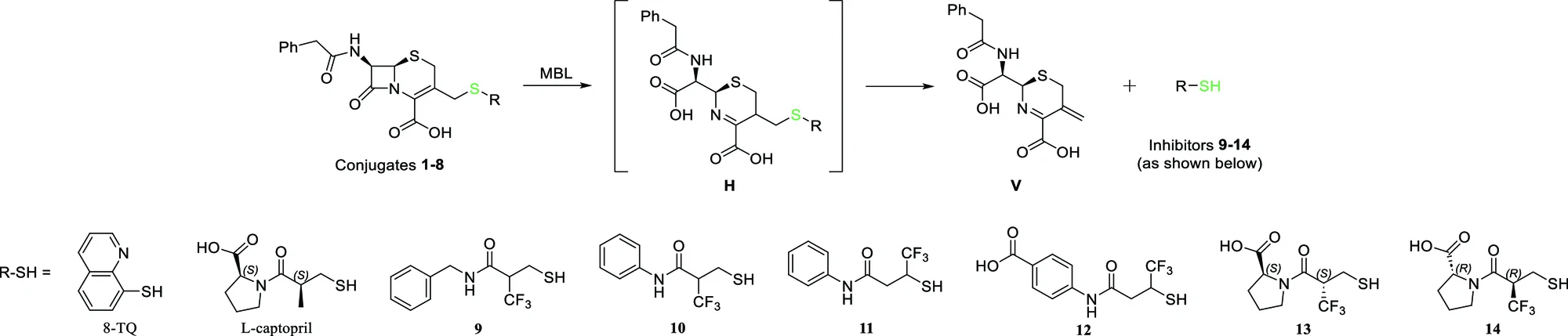
Cephalosporin conjugates of thiols previously reported as MBL inhibitors: **9–14**, 8-TQ, and L-captopril.

In a recent study,^18^ even though the conjugates demonstrated MBL inhibition, the thiols were not released after enzymatic hydrolysis, resulting in compounds with the general structure **H** (Figure 1, top), based on NMR and MS analyses. Intermediates possessing both the cleaved β-lactam ring and the leaving group were also obtained in a study of alkaline degradation of oxacephem derivatives.^20^ Depending on the leaving group, alkaline hydrolysis led to mixtures of various content, with or without the production of the vinylic elimination product **V**. These examples demonstrate that β-lactam ring cleavage and thiol release are not necessarily simultaneous processes and that, in order to design a conjugate releasing an aliphatic thiol inhibitor, alteration of the leaving group ability is necessary.

We hypothesized that the introduction of electron-withdrawing groups in the vicinity of the thiol could lead to the release of the inhibitor. Thus, we envisioned attaching trifluoromethylated aliphatic thiol MBL inhibitors to a cephalosporin scaffold. To investigate this strategy, conjugates **2**–**8** were designed (Figure 1), combining a cephalosporin core with previously reported thiol MBL inhibitors, including L-captopril and compounds **9**–**14**.^21^ The hydrolysis of the conjugates under basic and enzymatic conditions was studied by using LC–MS and NMR techniques. Furthermore, the inhibitory activity of the conjugates against different subclasses of B1 MBLs was investigated.

## 2 Results and Discussion

The cephalosporin–thiol conjugates were synthesized following a slightly adapted literature procedure^18^ (Scheme 1). The corresponding thiols were linked to 7-aminocephalosporanic acid (7-ACA) by Lewis acid catalysis, followed by acylation to yield compounds **1–8**. Compounds **3–6** were obtained as diastereomeric mixtures, given that the used thiols were a mixture of enantiomers. Conjugate **1** was synthesized as a reference compound, as it has previously been shown to release 8-thioquinoline (8-TQ) upon enzymatic hydrolysis.^15^

**Scheme 1:**
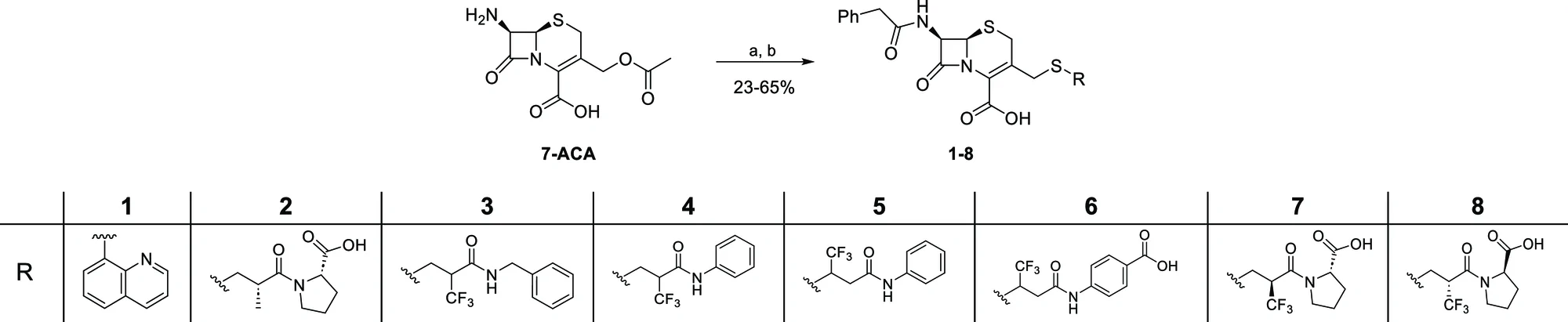
Synthesis of Conjugates **1–8**. Reaction Conditions: (a) BF_3_·Et_2_O, Acetonitrile, 50 °C, 2 h; (b) Phenylacetyl Chloride, Acetone, Saturated NaHCO_3(aq)_ Solution, r.t., Overnight

The hydrolysis of cephalosporin-based compounds has been extensively investigated by others, with conditions varying from buffer systems to enzyme-catalyzed assays.^22–24^ To gain a better understanding of the leaving group ability of the thiols for the synthesized compounds, we decided to test both base- and enzyme-mediated hydrolyses. Release of the thiol inhibitors was assessed using HPLC–MS and NMR to determine the presence of the released thiol **9**–**14** and elimination product **V**, respectively.

Previously, cephalosporin hydrolysis has been studied utilizing MS techniques.^15,18,19^ In a similar manner, we tested the hydrolysis of conjugates **2–8** in an aqueous 1 M Na_2_CO_3_ solution. Namely, the respective compound was incubated in the basic solution, and aliquots were taken at different time points (0.5, 1, and 4 h) and analyzed using HPLC–MS. In all cases, the formation of the vinylic elimination product (**V**) was observed through its relative abundance in the sample, determined by MS, as both UV- and ELS-detection of compound (**V**) were not successful. For conjugates **4–6** containing thiols **10**–**12**, monitoring of the release was based on the detection of the free thiol, as an aromatic ring present in the structure enables UV detection of the compounds. Considering the MS abundance of compound **V** and the observed thiol release (see Supporting Information, Figures S1–S8), the compounds could be divided into three groups. Compounds **5** and **6** seemed to be the most promising conjugates in terms of inhibitor release as both the vinylic product **V** and released thiol could be observed after 0.5 h of incubation. Compound **2** mostly resulted in hydrolysis of the β-lactam ring without the release of L-captopril (compound **2H**). For compounds **3**, **4**, **7**, and **8**, the vinylic elimination product **V** was detected; however, a major population of the respective compound **H** with the hydrolyzed β-lactam ring and unreleased thiol was also present. For compounds **7** and **8**, the vinylic product **V** was observed after 0.5 h, and for compounds **3** and **4**, after 4 h, indicating that compounds **7** and **8** release the thiol faster.

Next, we switched to a weaker base–ammonium carbonate, thus testing conditions closer to those of enzymatic hydrolysis. We used the 7-phenylacetamide derivative of the commercially available 7-ACA (compound **15**, Figure 2, A) as a positive control. After incubation in 3 M (NH_4_)_2_CO_3_ for 2 h, the peak of the conjugate **15** (*m*/*z* = 391.09) had disappeared, indicating that full consumption of compound **15** had occurred. Two new peaks emerged with a mass corresponding to the vinylic elimination product **V**_**N**_ (*m*/*z* = 348.10) (Figure 2, B), implying cleavage of the β-lactam ring by ammonia and release of the allylic leaving group (OAc). In contrast, under the same conditions, the conjugate prepared from L-captopril (compound **2**, Figure 2, A) did not produce the elimination product, according to MS. While the conjugate peak (*m*/*z* = 548.15) was no longer present after 2 h, showing full consumption of the conjugate, a new peak with *m*/*z* = 563.17 was observed. This corresponds to the formation of intermediate **2H**_**N**_, in which the β-lactam ring has been opened, but L-captopril is still attached to the compound. This result is in line with previous reports using aliphatic thiols as leaving groups^18^ and is attributed to their poor leaving group ability. Next, compound **5** was subjected to the same conditions (Figure 2, A), demonstrating a slower rate of ring cleavage than **15** and **2**, as conjugate **5** (*m*/*z* = 580.12) was still detectable by MS after 2 h of incubation in 3 M (NH_4_)_2_CO_3_. Nevertheless, the appearance of a new peak with *m*/*z* = 248.04 corresponding to the thiol **11** confirmed that degradation of compound **5** resulted in the release of the thiol.

**Figure 2:**
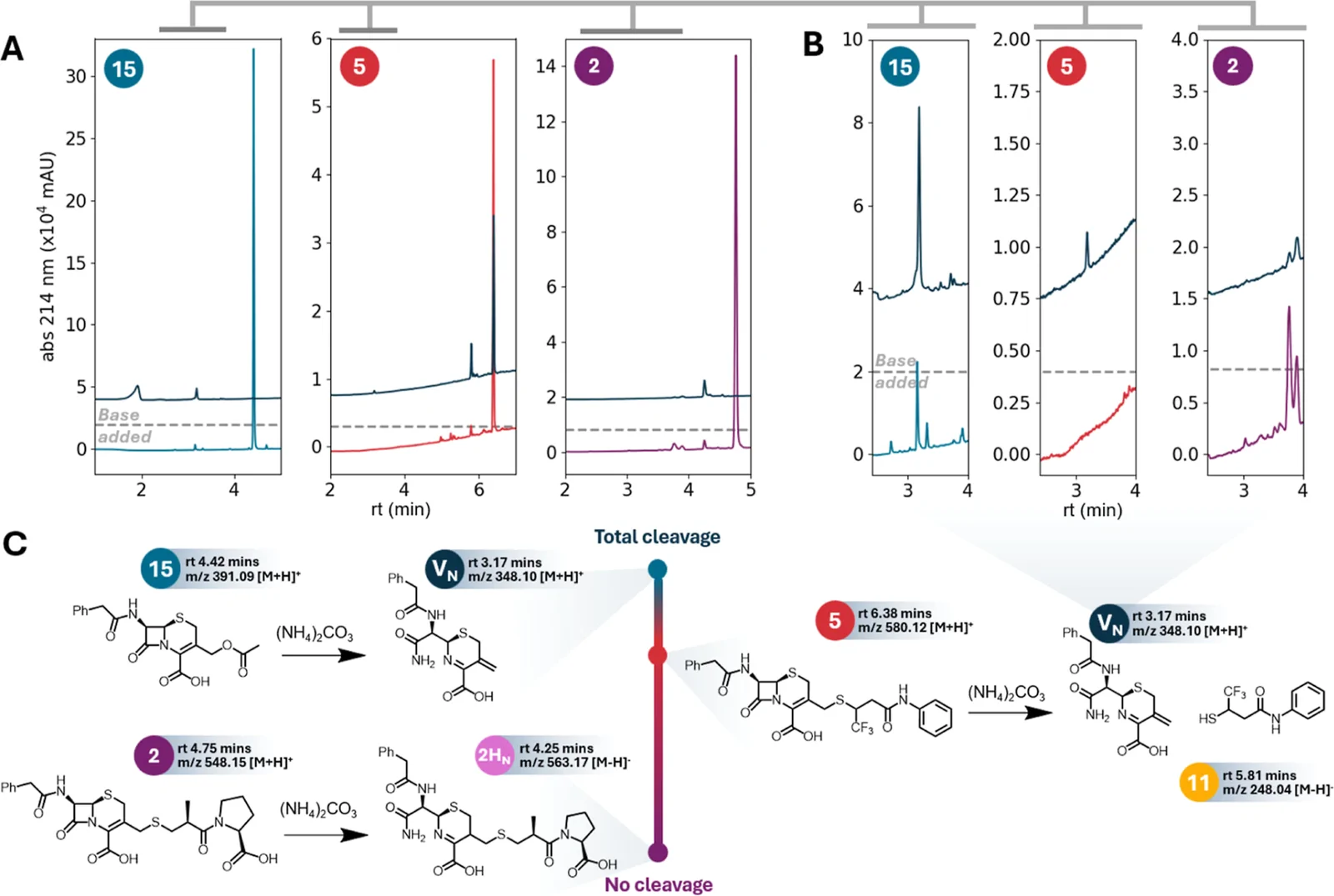
(A) LC–MS analyses of **15**, **5**, and **2**, respectively, before (trace below the dashed line) and after (NH_4_)_2_CO_3_-mediated degradation (trace above the dashed line). (B) Enlarged view of the **V**_**N**_ peak (rt 3.17 min) in the respective LC–MS traces shown in panel (A) to highlight the production of **V**_**N**_. (C) Reaction schemes for the degradation of **15**, **5**, and **2**, respectively, showing the conjugates and the observed degradation products. Conjugate **15** degraded completely upon treatment with (NH_4_)_2_CO_3_ and only **V**_**N**_ was observed. For conjugate **5**, **V**_**N**_ was formed slower, and unreacted conjugate was present. No formation of **V**_**N**_ was observed for conjugate **2**.

With these results in hand, we moved on to test enzyme-catalyzed hydrolysis of conjugates **4–6**. The conjugates were incubated with NDM-1 (for experiment details, see Supporting Information), and aliquots were taken at different time points (0, 20, 40, 60, and 90 min) and analyzed using HPLC–MS. For compound **4** (*m*/*z* = 580.12), after 20 min of incubation, two new peaks with *m*/*z* = 596.11 and *m*/*z* = 248.04 were observed, corresponding to intermediate **4H** and thiol **10**, respectively (Figure 3, A). In the subsequent measurements, the conjugate peak was found to be decreasing and the peak of **4H** increasing, with no increase being observed for the thiol peak. Furthermore, released thiol **10** was shown to degrade under the assay conditions, presumably forming the corresponding disulfide. Compounds **5** and **6** demonstrated similar hydrolysis patterns (Figures 4A and S12, respectively). In the case of conjugate **5**, the initial measurement after 20 min of incubation showed peaks with *m*/*z* = 248.05 (thiol **11**) and *m*/*z* = 596.10 (**5H**). In the following measurements, a slight increase of both the peaks could be observed.

**Figure 3:**
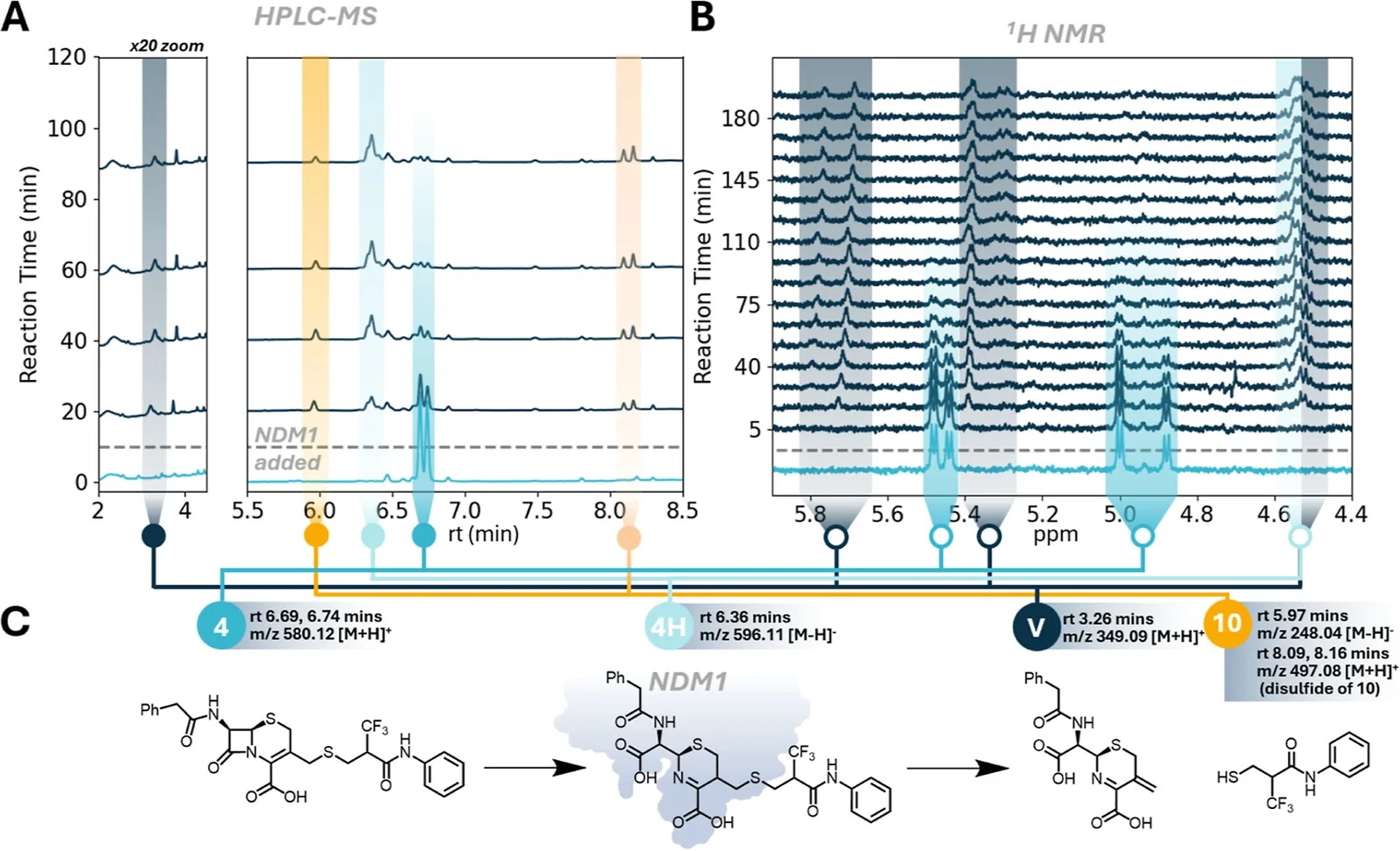
**A**) LC–MS and (**B**) ^1^H NMR analyses of NDM-1-mediated degradation of **4**. (**C**) Mechanism of NDM-1-mediated degradation of **4**.

**Figure 4:**
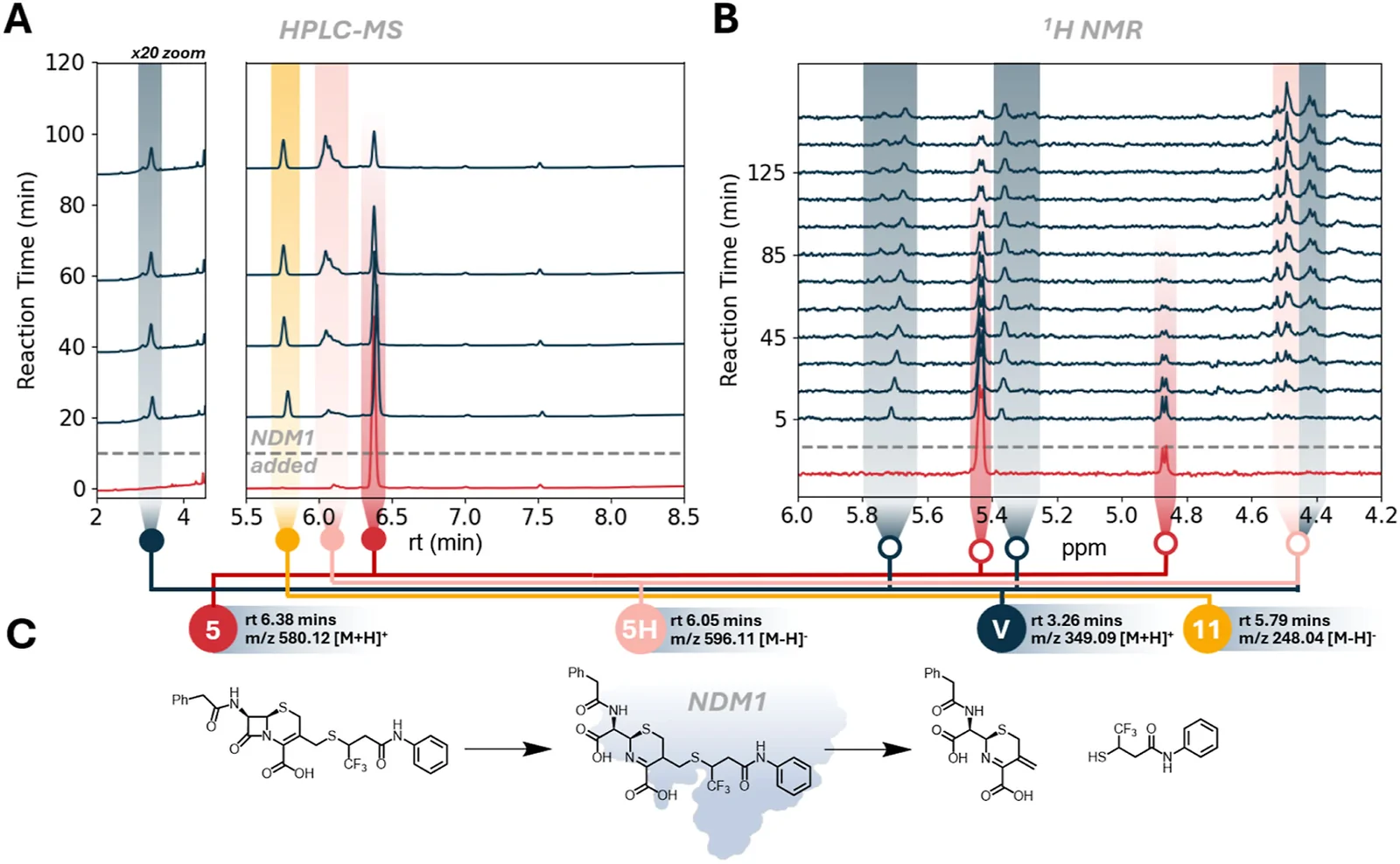
**A**) LC–MS and (**B**) ^1^H NMR analyses of NDM-1-mediated degradation of **5**. (**C**) Mechanism of NDM-1-mediated degradation of **5**.

In all three cases, the appearance of the thiol peak after the initial 20 min of incubation was observed, confirming the release of the inhibitor. However, only minimal changes occurred to the peak in subsequent measurements, indicating no further substantial release or alteration of the inhibitor. Nevertheless, the intermediates containing the cleaved β-lactam ring and the intact thioethers (of the general structure **H**) were also detected, complicating any quantitative analysis of the chromatograms.

To confirm our findings, we further examined MBL-mediated hydrolysis of conjugates **2**–**8** using an *in situ* NMR assay (for experiment details see Supporting Information). We utilized conjugate **1** as a positive control since it has been reported to form the vinylic elimination product (**V**) upon hydrolysis, as determined by NMR. The ^1^H NMR data (Figures 3B, 4B, and S13–S16) demonstrate the appearance of vinylic proton signals at ca. 5.70 ppm corresponding to the vinylic elimination product (**V**). However, the intensity of these peaks remains weak throughout the measurements and does not change greatly, while the proton signals of the conjugates gradually disappear over time. Comparing Figures 3 and 4, full consumption of conjugates **5** and **4** occurs at similar rates, indicating that the rate of MBL-mediated hydrolysis of the β-lactam ring in these cases is not strongly dependent on the position of the CF_3_ group. No hydrolysis was detected in control experiments in the absence of NDM-1 in the solution. It is also worth noting that addition of excess zinc ions during the course of hydrolysis (Figure S17) did not influence the chemical shift of the conjugate proton signals, indicating minimal zinc binding affinity of the conjugates. However, excess zinc caused clear shifts in the other peaks and led to sharper vinylic proton peak signals. The broader peaks observed in the absence of excess zinc and chemical shift changes are indicative of a small molecule in a fast exchange regime with a larger macromolecule, where the ^1^H signals of the bound and unbound states are observed as a single population-averaged peak. These observations suggest that the vinylic compound (**V**) is in part bound by NDM-1.

Both LC–MS and NMR analyses confirm thiol release and formation of the vinylic elimination product. All fluorinated conjugates appear to be superior to the nonfluorinated conjugate **2** chosen as the control in this study, demonstrating thiol release to varying extents. However, the analytical data of the most promising conjugates **5** and **6** is not directly comparable to the data reported for conjugate **1** containing an aromatic thiol inhibitor. The discrepancy between the appearance of the proton signals corresponding to compound **V** and the disappearance of the conjugate signals indicates that there are several processes occurring simultaneously, making quantitative interpretation of the data challenging. The formation of intermediates of the general structure **H** further complicates the analysis of conjugate hydrolysis. The results, acquired from basic and enzymatic hydrolysis experiments, indicate that the position of the CF_3_ group is not strongly influencing the hydrolysis of the β-lactam ring but is of significance for the release of the inhibitor as well as for the stability of the released thiol.

Fluorinated compounds have been used as probes for the investigation of structures and mechanisms in chemical biology.^25,26^ ^19^F NMR lacks background interference and has a broader chemical shift range than ^1^H NMR, making it a sensitive and convenient tool for solution-state NMR studies. Additionally, the presence of a CF_3_ group in the thiols provides additional sensitivity compared to the single protons that are observable in the ^1^H spectra. Hence, we set out to demonstrate that the *in situ* NMR assay can be extended to ^19^F NMR. For that, the course of NDM-1-mediated hydrolyses of conjugates **4** and **5** was monitored by ^19^F NMR (Figures 5A and 5C, respectively). The obtained spectra show the disappearance of the two starting peaks, corresponding to the two diastereomers of the conjugates, and the appearance of several new peaks. The LC–MS data indicated that the emerging peaks correspond to the two diastereomers of **4H** or **5H**, the thiol (**10** or **11**) and its corresponding disulfide. However, assigning the peaks to the respective products was challenging as the chemical shifts of isolated compounds in the buffer could only be determined for thiol **11**. Guided by the LC–MS experiments with conjugate **4** (Figure 3A), we assigned the two rapidly emerging peaks (light blue) to the diastereomeric intermediates **4H** (Figure 5A). Based on the time-dependence of the fluorine peak intensities, we suggest that the broader peaks (yellow) correspond to the thiol **10** and its disulfide, though it is not possible to discern between the two. For conjugate **5**, the broad upfield peak (ca. −72.2 ppm, yellow) was identified as the thiol (**11**), whereas the broad peak around −70.5 ppm (pale yellow), emerging later than the other peaks, was presumed to be the disulfide (Figure 5C). Surprisingly, three additional peaks were observed, two of which should account for the two diastereomers of **5H** (−71.07 ppm and −71.03 ppm), which is in agreement with LC–MS analysis and time-dependence of the fluorine peak intensities. We hypothesize that the additional peak at −70.46 ppm arises from a C-6 epimer of **5H** as previously observed in the hydrolysis of cephalosporin-based β-lactam antibiotics.^27^

**Figure 5:**
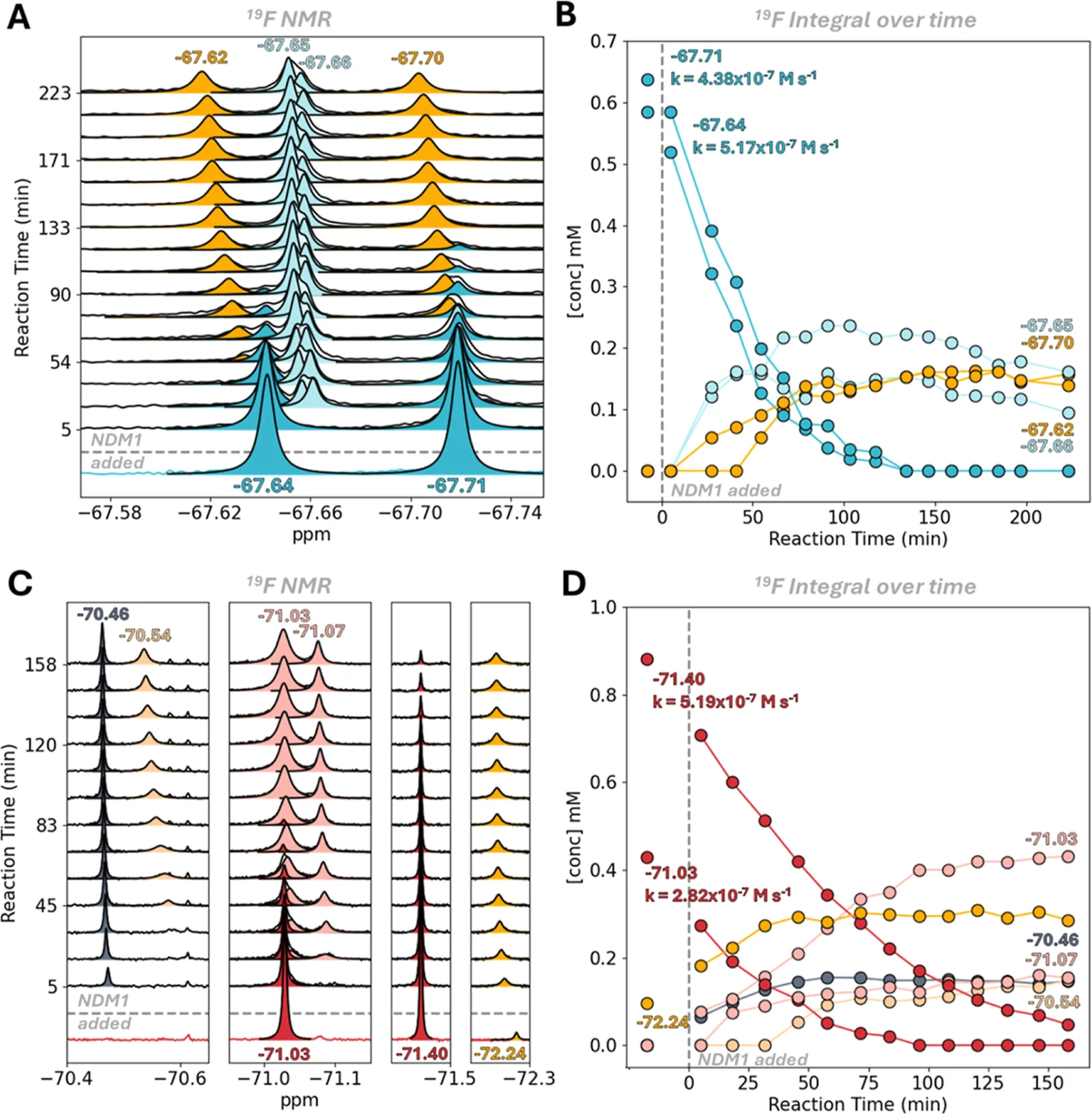
^19^F NMR-based monitoring of NDM-1-mediated degradation of **4** (A) and **5** (C) and the changes in concentration over time of all components with tentative assignments and determined reaction rates for **4** and **5** (B and D). Colors correspond to the products in Figures 3 and 4, respectively, based on the hypothetical assignments as described in the text. The signal at −70.46 ppm colored in black is hypothesized to correspond to the C-6 epimer of **5H**.

The sharp nature of ^19^F peaks corresponding to the diastereomers of **4** and **5**, together with the absence of significant line-broadening or chemical shift perturbation (S18–S19), suggests that **4** and **5** are either in slow exchange with NDM-1, consistent with binding in the low nanomolar range. More likely, however, hydrolysis of the conjugate to the intermediate occurs at a rate greater than the dissociation (off) rate of the conjugate from NDM-1. First-order reaction kinetics were observed for the depletion of the conjugates (S20–S21). On the basis of the extracted reaction rates and the relative concentrations of the diastereomers, NDM-1 appears to exhibit minimal stereoselectivity for conjugates **4** and **5**. Overall, the ^19^F NMR studies were in agreement with the results from LC–MS and ^1^H NMR assays and provided some complementary mechanistic information.

The inhibitory activity of conjugates **1**–**8** was evaluated against a panel of representative subclass B1 MBLs (Table 1) with nitrocefin used as the reporter substrate. Following 10 min preincubation of the enzyme with the conjugate, the initial rates of the reactions with various concentrations of the inhibitors in a 2-fold dilution series were measured. The half-maximal inhibitory concentration (IC_50_) values were determined by fitting the resulting dose–response curves. L-captopril and conjugate **1** were used as reference compounds, for which inhibitory activity data has been published previously.^15^ Compound **16** (for structure see Table 1) was included in the test set to verify that the cephalosporin core of the conjugates is not responsible for the inhibition of MBLs.

**Table 1:** Inhibitory Activity of Conjugates **1**–**8**, L-Captopril, and **16** against Selected B1 MBLs

	IC_50_ (μM)^a^			
compound	NDM-1	VIM-2	IMP-2	IMP-7
**1**	1.92 ± 0.06	1.99 ± 0.07	1.4 ± 0.09	2.4 ± 0.17
**2**	31.81 ± 4.93	10.37 ± 1.64	22.44 ± 3.11	6.91 ± 0.49
**3**	27.76 ± 8.4	4.58 ± 0.22	19.24 ± 0.73	18.61 ± 1.32
**4**	3.76 ± 0.35	**0.61** **±** **0.05**	11.28 ± 2.17	13.47 ± 1.75
**5**	8.22 ± 0.34	7.11 ± 0.37	6.07 ± 0.83	8.61 ± 0.45
**6**	4.5 ± 0.38	33.64 ± 4.49	2.79 ± 0.93	4.12 ± 0.11
**7**	91.72 ± 5.08	15.34 ± 1.22	82.33 ± 26.17	21.84 ± 0.84
**8**	**2.02 ± 0.55**	4.28 ± 0.86	**1.98 ± 0.52**	**3.19** **±** **0.11**
**16** ^b^	>300	49.94 ± 5.18	>300	60.05 ± 6.94
L-captopril	13.12 ± 3.23	4.487 ± 0.19	6.16 ± 0.31	10.15 ± 0.76

a The half-maximal inhibitory concentrations of the compounds are provided as the mean values along with the standard error of the mean. The reported values are based on triplicate experiments.

b Structure of compound **16**.
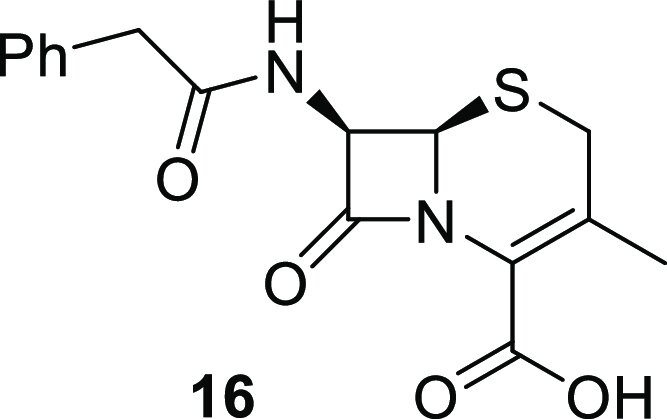

The tested conjugates demonstrated inhibition to various extents with IC_50_ values ranging from 0.61 to 91.72 μM. Conjugate **8** displayed activity against all of the tested MBLs with IC_50_ values of 2.02, 4.28, 1.98, and 3.19 μM against NDM-1, VIM-2, IMP-2, and IMP-7, respectively. Comparing the inhibitory activity of conjugates **4** and **5**, the β–CF_3_–derivative (**5**) appears slightly more potent than the α–CF_3_–derivative (**4**) against IMP-type MBLs, while conjugate **4** appears more active against NDM-1 and VIM-2. Notably, the IC_50_ values obtained for the conjugates are in a similar range to those reported for the corresponding thiol inhibitors (see Table S1 for comparison).^21^ We also evaluated the effect of the enzyme–inhibitor preincubation time on the inhibitory activity by determining IC_50_ values after 0, 10, and 30 min of preincubation (Table S2 and Figures S26–S29). For conjugate **2**, the IC_50_ values showed little variation with preincubation time. In contrast, the remaining conjugates exhibited varying degrees of time-dependent inhibition, with IC_50_ values decreasing by approximately 10- to 400-fold depending on the conjugate/enzyme combination. Compound **7** was most consistent, with an IC_50_ reduction of approximately 10-fold detected for all enzymes after 10 min incubation (NDM-1, VIM-2) and 30 min (IMP-2 and IMP-7) when compared to minimal incubation time. The strongest dependency on preincubation time was observed with conjugate **8**, with the highest decrease in IC_50_ identified in the case of IMP-2 from 281 μM with minimal incubation time to 0.67 μM with 30 min incubation time. The observed time-dependent inhibition for several conjugates is consistent with the enzyme-mediated hydrolysis demonstrated by the LC–MS and NMR analyses.

To determine whether the observed biochemical inhibition translated into antibacterial potentiation, the conjugates were next evaluated in combination with meropenem (Table S3). For conjugates **2**–**7**, no significant reduction in the MIC of meropenem was observed, while conjugate **8** reduced the MIC of meropenem by 8-fold at a concentration of 256 μg/mL. These findings suggest that factors other than β-lactam hydrolysis and inhibitor release contribute to whole cell activity and imply that optimization of the conjugate scaffold, possibly addressing poor membrane permeability or deactivation through disulfide formation, remains necessary.

## 3 Conclusion

A series of novel conjugates, designed to release trifluoromethylated thiol-based MBL inhibitors upon enzymatic hydrolysis, was synthesized. Thiol release, as determined using LC–MS and ^1^H NMR assays, confirmed improved leaving group properties of aliphatic thiols upon fluorination when compared with a nonfluorinated control. However, when compared with a conjugate containing the aromatic thiol 8-TQ, release of fluorinated thiols was slower and mechanistically more complex. Neither the stalling of the release nor the formation of intermediates containing the cleaved β-lactam ring and the intact thioethers has been reported for conjugates with aromatic thiols. Formation of disulfides was not prevented by the conjugate approach, and in total, the accumulation of the free fluorinated thiol appeared low. Overall, these findings provide new insights for further development of cephalosporin-based MBL-inhibitor conjugates.

Furthermore, the course of hydrolysis was monitored by using ^19^F NMR, demonstrating its potential use for the analysis of enzyme-catalyzed reactions.

The conjugates exhibited inhibitory activity against B1 MBLs such as NDM-1, VIM-2, IMP-2, and IMP-7. Nevertheless, improvements are still needed, in particular with a focus on improving thiol release and enhancing synergistic activity. Among the compounds of this study, conjugate **8** displayed the strongest time-dependent inhibition together with improved potentiation of meropenem, suggesting this scaffold as the most promising candidate for further optimization to realize the full potential of thiol-releasing β-lactam conjugates as MBL-activated prodrugs.

## 4 Experimental Section

A description of the experimental procedures, analytical data, and IC_50_ curves can be found in the Supporting Information.

## Supplementary Material



## Notes

Detailed synthetic procedure and characterization of conjugates **2–8**; LC–MS hydrolysis experiments under basic (Figures S1–S9) and enzymatic conditions (Figures S10–S12); NMR-based hydrolysis experiments: ^1^H (Figures S13–S17) and ^19^F (Figures S18–S21); detailed description of inhibition assays, alongside IC_50_ values of conjugates and corresponding thiols (Table S1) and influence of pre-incubation time on inhibitory activity (Table S2, Figures S26–29); MIC assay (Table S3); and ^1^H and ^13^C NMR spectra of synthesized compounds (PDF)

## References

[1] Mora-Ochomogo M., Lohans C. T. (2021). β-Lactam Antibiotic Targets and Resistance Mechanisms:
from Covalent Inhibitors to Substrates. RSC
Med. Chem..

[2] Yang Y., Yan Y.-H., Schofield C. J., McNally A., Zong Z., Li G.-B. (2023). Metallo-β-Lactamase-Mediated
Antimicrobial Resistance and Progress
in Inhibitor Discovery. Trends Microbiol..

[3] Bahr G., González L. J., Vila A. J. (2021). Metallo-β-Lactamases in the
Age of Multidrug Resistance: From Structure and Mechanism to Evolution,
Dissemination, and Inhibitor Design. Chem. Rev..

[4] Denakpo E., Naas T., Iorga B. I. (2023). An Updated
Patent Review of Metallo-β-Lactamase
Inhibitors (2020–2023). Expert Opin.
Ther. Pat..

[5] Palzkill T. (2013). Metallo-β-Lactamase
Structure and Function. Ann. N. Y. Acad. Sci..

[6] Bush K., Bradford P. A. (2019). Interplay Between
β-Lactamases and New β-Lactamase
Inhibitors. Nat. Rev. Microbiol..

[7] Shi C., Chen J., Kang X., Shen X., Lao X., Zheng H. (2019). Approaches for the Discovery of Metallo-β-Lactamase Inhibitors:
A Review. Chem. Biol. Drug Des..

[8] Lienard B. M., Garau G., Horsfall L., Karsisiotis A. I., Damblon C., Lassaux P., Papamicael C., Roberts G. C., Galleni M., Dideberg O., Frere J. M., Schofield C. J. (2008). Structural Basis for the Broad-Spectrum Inhibition
of Metallo-β-Lactamases by Thiols. Org.
Biomol. Chem..

[9] Chen, C. ; Wang, D. ; Yang, K.-W. (2023). Chapter 14 - Thiols as a Privileged Scaffold Against Metallo-β-Lactamases. In: Yu, B. ; Li, N. ; Fu, C. , Eds. Privileged Scaffolds in Drug Discovery; Academic Press, 301–318.

[10] Tehrani K. H. M. E., Martin N. I. (2017). Thiol-Containing Metallo-β-Lactamase Inhibitors
Resensitize Resistant Gram-Negative Bacteria to Meropenem. ACS Infect. Dis..

[11] Lothian A., J Hare D., Grimm R., Ryan T. M., Masters C. L., Roberts B. R. (2013). Metalloproteomics:
Principles, Challenges and Applications
to Neurodegeneration. Front. Aging Neurosci..

[12] Peters B. K., Reddy N., Shungube M., Girdhari L., Baijnath S., Mdanda S., Chetty L., Ntombela T., Arumugam T., Bester L. A., Singh S. D., Chuturgoon A., Arvidsson P. I., Maguire G. E. M., Kruger H. G., Naicker T., Govender T. (2023). In Vitro and In Vivo Development of a β-Lactam-Metallo-β-Lactamase
Inhibitor: Targeting Carbapenem-Resistant Enterobacterales. ACS Infect. Dis..

[13] Liu W., Liu C., Guo Y., Liu Z., Zhang C., Meng W., Wan S., Chen S., Qiu J., Yu R. (2026). The New Delhi Metallo-β-lactamase-1
Covalent Inhibitors Derived from Cephalexin Effectively Reverse Meropenem
Resistance. J. Med. Chem..

[14] Cole M. S., Hegde P. V., Aldrich C. C. (2022). β-Lactamase-Mediated
Fragmentation:
Historical Perspectives and Recent Advances in Diagnostics, Imaging,
and Antibacterial Design. ACS Infect. Dis..

[15] van
Haren M. J., Tehrani K. H. M. E., Kotsogianni I., Wade N., Bruchle N. C., Mashayekhi V., Martin N. I. (2021). Cephalosporin Prodrug Inhibitors Overcome Metallo-β-Lactamase
Driven Antibiotic Resistance. Chemistry.

[16] Jackson A. C., Zaengle-Barone J. M., Puccio E. A., Franz K. J. (2020). A Cephalosporin
Prochelator Inhibits New Delhi Metallo-β-lactamase 1 without
Removing Zinc. ACS Infect. Dis..

[17] Zaengle-Barone J. M., Jackson A. C., Besse D. M., Becken B., Arshad M., Seed P. C., Franz K. J. (2018). Copper
Influences the Antibacterial
Outcomes of a β-Lactamase-Activated Prochelator against Drug-Resistant
Bacteria. ACS Infect. Dis..

[18] Tehrani K. H. M. E., Wade N., Mashayekhi V., Bruchle N. C., Jespers W., Voskuil K., Pesce D., van Haren M. J., van Westen G. J. P., Martin N. I. (2021). Novel Cephalosporin
Conjugates Display
Potent and Selective Inhibition of Imipenemase-Type Metallo-β-Lactamases. J. Med. Chem..

[19] Tian H., Wang Y., Dai Y., Mao A., Zhou W., Cao X., Deng H., Lu H., Ding L., Shen H., Wang X. (2022). A Cephalosporin-Tripodalamine Conjugate Inhibits Metallo-β-Lactamase
with High Efficacy and Low Toxicity. Antimicrob.
Agents Chemother..

[20] Nishikawa J., Watanabe F., Shudou M., Terui Y., Narisada M. (1987). Proton NMR
Study of Degradation Mechanisms of Oxacephem Derivatives with Various
3′-Substituents in Alkaline Solution. J. Med. Chem..

[21] Kondratieva A., Palica K., Frøhlich C., Hovd R. R., Leiros H.-K. S., Erdelyi M., Bayer A. (2024). Fluorinated
Captopril Analogues Inhibit
Metallo-β-Lactamases and Facilitate Structure Determination
of NDM-1 Binding Pose. Eur. J. Med. Chem..

[22] Yamana T., Tsuji A. (1976). Comparative Stability of Cephalosporins
in Aqueous Solution: Kinetics
and Mechanisms of Degradation. J. Pharm. Sci..

[23] Hanessian S., Wang J. (1993). Design and Synthesis of a Cephalosporin–Carboplatinum
Prodrug
Activatable by a β-Lactamase. Can. J.
Chem..

[24] Feng H., Ding J., Zhu D., Liu X., Xu X., Zhang Y., Zang S., Wang D.-C., Liu W. (2014). Structural
and Mechanistic Insights into NDM-1 Catalyzed Hydrolysis of Cephalosporins. J. Am. Chem. Soc..

[25] Gimenez D., Phelan A., Murphy C. D., Cobb S. L. (2021). ^19^F NMR
as a Tool in Chemical Biology. Beilstein J.
Org. Chem..

[26] Cobb S. L., Murphy C. D. (2009). ^19^F NMR
Applications in Chemical Biology. J. Fluor.
Chem..

[27] Badarau A., Llinás A., Laws A. P., Damblon C., Page M. I. (2005). Inhibitors
of Metallo-β-Lactamase Generated from β-Lactam Antibiotics. Biochemistry.

