# Supplementary material for: Fluorinated Aliphatic Thiols in the Design of Cephalosporin Conjugates for Metallo-β-Lactamase Inhibition
Source: ACS Omega. 2026 Sep 30;11(40):61913–20. doi: 10.1021/acsomega.6c07917 (PMC13670874; doi:10.1021/acsomega.6c07917)
Supplement: Supplementary file 1 [file ao6c07917_si_001.pdf]

Supporting information for "*Fluorinated Aliphatic Thiols in the Design of Cephalosporin Conjugates for Metallo-β-Lactamase Inhibition*"

Alexandra Kondratieva[a], Philip Rainsford[a,b], Perwez Bakht[c], Ranjana Pathania[c], Annette Bayer[a]*

[a] *Department of Chemistry, UiT The Arctic University of Norway, NO-9037 Tromsø, Norway.*
[b] *Section for Molecular and Translational Pharmacology, Department of Biomedical Sciences, University of Copenhagen, 2200 Copenhagen, Denmark.*
[c] *Department of Biosciences and Bioengineering, Indian Institute of Technology, Roorkee, Uttarakhand-247667, India.*
**Corresponding author.*

Table of Contents

Chemistry S2
- General methods S2
- General procedure S3
- Spectroscopic data S3
- Carbon assignment S7
- LC-MS-based hydrolysis experiments S13
- NMR-based enzymatic hydrolysis experiments S23

MBL inhibition assay and IC_{50} curves S29
Meropenem restoration assay in bacteria S38
1H and 13C NMR spectra of final compounds S39
References S46

Chemistry

General methods

All reagents were purchased from commercial sources and used as supplied without further purification unless otherwise stated. Thiols **9-14** were prepared according to previously published procedures.[1] (6*R*,7*R*)-8-oxo-7-(2-phenylacetamido)-3-((quinolin-8-ylthio)methyl)-5-thia-1-azabicyclo[4.2.0]oct-2-ene-2-carboxylic acid (**1**)[2], (6*R*,7*R*)-3-(acetoxymethyl)-8-oxo-7-(2-phenylacetamido)-5-thia-1-aza-bicyclo[4.2.0]oct-2-ene-2-carboxylic acid (**15**)[3] and (6*R*,7*R*)-3-methyl-8-oxo-7-(2-phenylacetamido)-5-thia-1-azabicyclo[4.2.0]oct-2-ene-2-carboxylic acid (**16**)[4] were synthesized following previously reported procedures and their analytical data were found to be in accordance with those reported. Solvents were dried according to standard procedures over appropriately sized molecular sieves.

For thin layer chromatography (TLC) analysis, aluminium plates pre-coated with silica gel (Merck silica gel 60 F_{254}) were used and visualized using either ultraviolet light or by treatment with an appropriate stain. Compounds were purified via preparative HPLC performed on a CombiFlash® EZ Prep system with a YMC-Actus Triart C18 column, eluting with mixtures of acetonitrile and H_2O (both containing 0.1% TFA).

^{1}H and ^{13}C NMR spectra for assignment were obtained on a 400 MHZ Bruker Advance III HD spectrometer equipped with a 5 mm SmartProbe BB/1H (BB = 19F, 31P-15N) at 20 ˚C. Chemical shifts are reported in ppm relative to the solvent residual peak ($CDCl_3$: δH 7.26 and δC 77.16; Acetone-*d6*: δH 2.05 and δC 206.26; Methanol-*d4*: δH 3.31 and δC 49.00). Coupling constants J are reported in Hertz (Hz). The ^{13}C NMR spectra were ^{1}H decoupled. The following abbreviations were used to indicate the multiplicities: s = singlet, d = doublet, t = triplet, q = quartet, qnt = quintet, dd = doublet of doublets, dq = doublet of quartets, dqd = doublet of quartet of doublets, tt = triplet of triplets, qntd = quintet of doublets, m = multiplet, app = appearing and br = broad. When possible, for compounds obtained as rotameric mixtures, chemical shifts of minor rotamers are reported separately, enclosed in square brackets.

High-resolution mass spectrometry (HRMS) was conducted from methanol or acetonitrile solutions on a ThermoScientific Vanquish UHPLC system coupled to a ThermoScientific Orbitrap Exploris 120 with electrospray ionization (ESI). The following solvent system, at a flow rate of 0.3 mL/min, was used: solvent A – MiliQ water/0.1% formic acid; solvent B – 90% acetonitrile/10% MiliQ water/0.1% formic acid. Gradient elution was as follows: 95:5 to 30:70 (A:B) over 7 min, 0:100 (A:B) for 2 min, then reversion back to 95:5 (A:B) for 3 min.

Melting points were measured on a Stuart SMP50 automatic melting point detector and are reported uncorrected in degree Celsius (˚C).

All final compounds were analyzed using the described above UHPLC-HRMS system and the purity is specified for each compound below.

NDM-1 protein was expressed and purified as described in literature.[5]

General procedure

In an oven-dried two-neck round-bottom flask $BF_3 \cdot Et_2O$ (3.0 eq) was mixed with MeCN (2-4 mL) under an argon atmosphere, followed by the sequential addition of the respective thiol (1.5 eq) and 7-ACA (1.0 eq). The reaction mixture was stirred at 50 ºC for 2 h, after which water and an ammonium hydroxide solution (32%) were added, adjusting the pH to 4. The formed precipitate was collected, washed with water and the crude product was suspended in a mixture of a saturated bicarbonate solution and acetone (2:3). Next, phenylacetyl chloride (2.0 eq) was added dropwise and the resulting mixture was stirred overnight at ambient temperature. After that, the mixture was diluted with water and the pH was adjusted to 2 using 1 M HCl. If a precipitate formed, it was filtered and washed with a small amount of cold water. Alternatively, the volatiles were evaporated using a nitrogen stream. The obtained crude material was purified by reversed-phase prep-HPLC.

Spectroscopic data

Compounds **3-6** were obtained as diastereomeric mixtures and NMR data is reported for the mixture. For compounds **7-8**, obtained as rotameric mixtures, when possible, chemical shifts of the minor rotamers are reported separately, enclosed in square brackets. Carbon assignment for compounds **3-8** can be found on pages S7-S12.

(6R,7R)-3-((((S)-3-((S)-2-carboxypyrrolidin-1-yl)-2-methyl-3-oxopropyl)thio)methyl)-8-oxo-7-(2-phenylacetamido)-5-thia-1-azabicyclo[4.2.0]oct-2-ene-2-carboxylic acid **(2)**.

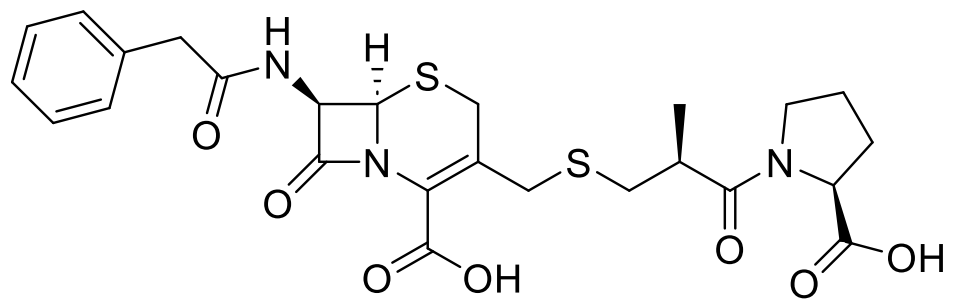

Synthesized according to the *General Procedure* using 7-ACA (220 mg, 0.81 mmol, 1.0 eq), *L*-captopril (264 mg, 1.21 mmol, 1.5 eq), boron trifluoride ethyl etherate (305 µl, 2.42 mmol, 3.0 eq), MeCN (3 mL) and phenylacetyl chloride (194 µl, 1.47 mmol, 2.0 eq). The crude was purified using H_2O/MeCN (0.1% TFA) to yield **2** (151 mg, 0.28 mmol, 35%, over two steps) as a white solid. **^{1}H NMR** (400 MHz, DMSO-*d6*) δ 9.10 (app d, J = 8.2 Hz, 1H), 7.35 – 7.17 (m, 5H), 5.61 (dd, J = 8.2, 4.7 Hz, 1H), 5.15 – 5.07 (m, 1H), 4.23 (dd, J = 8.8, 3.9 Hz, 1H), 3.74 – 3.29 (m, 8H), 2.83 – 2.63 (m, 2H), 2.42 – 2.32 (m, 1H), 2.21 – 2.05 (m, 1H), 1.99 – 1.71 (m, 3H), 1.10 – 0.96 (m, 3H). **^{13}C NMR** (101 MHz, DMSO-*d6*) δ 173.4, 172.7, 171.0, 164.6, 163.2, 135.9, 129.0 (2C), 128.2 (2C), 128.1, 126.5, 124.7, 59.0, 58.3, 58.0, 46.5, 41.6, 37.6, 33.8, 32.8, 28.7, 26.9, 24.4, 16.9. **HRMS** (ESI): calcd for $C_{25}H_{30}N_3O_7S_2^+$ $[M+H]^+$ 548.1520, found: 548.1518. **M.p.**: 122 – 125 ˚C. Purity: >99% (UV 256 nm).

(6R,7R)-3-(((2-(benzylcarbamoyl)-3,3,3-trifluoropropyl)thio)methyl)-8-oxo-7-(2-phenylacetamido)-5-thia-1-azabicyclo[4.2.0]oct-2-ene-2-carboxylic acid **(3)**.

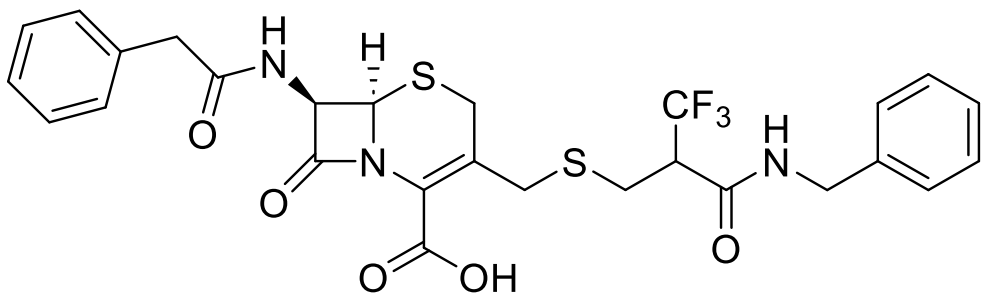

Synthesized according to the *General Procedure* using 7-ACA (85 mg, 0.31 mmol, 1.0 eq), *N*-benzyl-3,3,3-trifluoro-2-(mercaptomethyl)propanamide (123 mg, 0.47 mmol, 1.5 eq), boron trifluoride ethyl etherate (118 µl, 0.94 mmol, 3.0 eq), MeCN (2 mL) and phenylacetyl chloride (79 µl, 0.60 mmol, 2.0 eq). The crude was purified using H_2O/MeCN (0.1% TFA) to yield **3** (75 mg, 0.13 mmol, 36%, over two steps) as a white solid. **^{1}H NMR** (400 MHz, DMSO-*d6*) δ 9.16, 9.10 (d, *J* = 8.4 Hz, 1H), 9.03, 8.95 (d, *J* = 5.9 Hz, 1H), 7.43 – 7.16 (m, 10H), 5.71 – 5.58 (m, 1H), 5.17 – 5.05 (m, 1H), 4.46 – 4.31 (m, 2H), 3.98, 3.33 (app d, *J* = 13.6 Hz, 1H), 3.81 (app d, *J* = 13.5 Hz, 0.5H), 3.74 – 3.41 (m, 5.5H), 3.03 – 2.72 (m, 2H). **^{13}C NMR** (101 MHz, DMSO-*d6*) δ 171.0, 164.8, 164.6, 164.4, 163.1, 163.0, 138.6, 138.5, 135.9, 135.85, 129.1, 128.4, 128.35, 128.3, 127.9, 127.5, 127.2, 127.05, 127.0, 126.5, 125.2, 124.9, 124.8 (q, *J* = 281.9 Hz), 59.0, 58.95, 58.0, 49.8 (q, J = 24.6 Hz), 49.6 (q, J = 24.6 Hz), 42.5, 41.6, 33.1, 32.6, 27.1, 26.7, 26.1. **HRMS** (ESI): calcd for $C_{27}H_{27}F_3N_3O_5S_2^+$ [M+H]$^+$ 594.1339, found: 594.1337. **M.p.**: 113 – 115 ˚C. Purity: >99% (UV 256 nm).

(6R,7R)-8-oxo-7-(2-phenylacetamido)-3-(((3,3,3-trifluoro-2-(phenylcarbamoyl)propyl)thio)methyl)-5-thia-1-azabicyclo[4.2.0]oct-2-ene-2-carboxylic acid **(4)**.

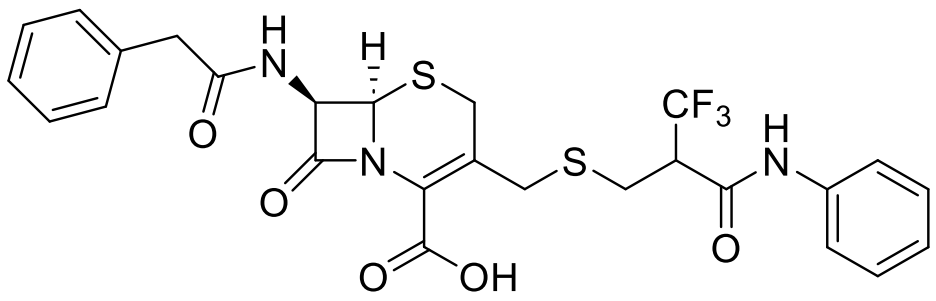

Synthesized according to the *General Procedure* using 7-ACA (95 mg, 0.35 mmol, 1.0 eq), 3,3,3-trifluoro-2-(mercaptomethyl)-*N*-phenylpropanamide (131 mg, 0.53 mmol, 1.5 eq), boron trifluoride ethyl etherate (132 µl, 1.05 mmol, 3.0 eq), MeCN (3 mL) and phenylacetyl chloride (178 µl, 1.34 mmol, 4.0 eq). The crude was purified using H_2O/MeCN (0.1% TFA) to yield **4** (131 mg, 0.23 mmol, 65%, over two steps) as a white solid. **^{1}H NMR** (400 MHz, DMSO-*d6*) δ 10.57, 10.51 (s, 1H), 9.16, 9.09 (d, *J* = 8.4 Hz, 1H), 7.64 – 7.57 (m, 2H), 7.39 – 7.31 (m, 2H), 7.31 – 7.19 (m, 5H), 7.16 – 7.09 (m, 1H), 5.70 – 5.63 (m, 1H), 5.13, 5.11 (d, *J* = 4.7 Hz, 1H), 4.05 (app d, *J* = 13.6 Hz, 0.5H), 3.89 – 3.80 (m, 1H), 3.71 – 3.44 (m, *overlapping water peak*, 5H), 3.30 (app d, *J* = 13.7 Hz, 0.5H), 3.09 – 2.74 (m, 2H). **^{13}C NMR** (101 MHz, DMSO-*d6*) δ 171.0, 170.95, 164.8, 164.7, 163.3, 163.1, 163.05, 163.0, 138.2, 138.1, 135.85, 135.8, 129.0, 128.95, 128.9, 128.2, 128.0, 127.4, 126.5, 125.1, 124.9, 124.7 (q, *J* = 281.5 Hz), 124.3, 124.2, 119.5, 119.4, 59.0, 58.0, 50.6 (q, *J* = 24.4 Hz), 50.4 (q, *J* = 24.4 Hz), 41.65, 41.6, 33.1, 32.4, 27.1, 26.7, 26.0. **HRMS** (ESI): calcd for $C_{26}H_{25}F_3N_3O_5S_2^+$ [M+H]$^+$ 580.1182, found: 580.1181. **M.p.**: 131 – 133 ˚C. Purity: >95% (UV 256 nm).

(6R,7R)-8-oxo-7-(2-phenylacetamido)-3-(((1,1,1-trifluoro-4-oxo-4-(phenylamino)butan-2-yl)thio)methyl)-5-thia-1-azabicyclo[4.2.0]oct-2-ene-2-carboxylic acid **(5)**.

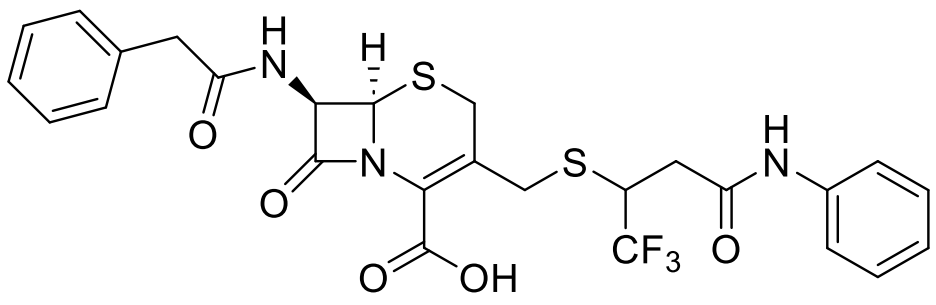

Synthesized according to the *General Procedure* using 7-ACA (172 mg, 0.63 mmol, 1.0 eq), 4,4,4-trifluoro-3-mercapto-*N*-phenylbutanamide (236 mg, 0.95 mmol, 1.5 eq), boron trifluoride ethyl etherate (238 µl, 1.90 mmol, 3.0 eq), MeCN (4 mL) and phenylacetyl chloride (321 µl, 2.43 mmol, 4.0 eq). The crude was purified using H_2O/MeCN (0.1% TFA) to yield **5** (134 mg, 0.23 mmol, 37%, over two steps) as a white solid. **^{1}H NMR** (400 MHz, CD_3CN) δ 8.50, 8.47 (s, 1H), 7.62 – 7.51 (m, 2H), 7.39 – 7.21 (m, 8H), 7.17 – 7.08 (m, 1H), 5.74, 5.68 (dd, *J* = 8.7, 4.8 Hz, 1H), 5.09, 4.96 (d, *J* = 4.8 Hz, 1H), 4.08 – 3.77 (m, 2H), 3.73 – 3.36 (m, 5H), 2.98 – 2.84 (m, 1H), 2.62 – 2.44 (m, 1H). **^{13}C NMR** (101 MHz, CD_3CN) δ 172.4, 172.35, 167.6, 167.5, 165.9, 165.7, 163.4, 163.3, 139.4, 139.3, 136.3, 130.1, 129.9, 129.85, 129.8, 129.4, 128.1 (q, *J* = 275.8 Hz), 127.8, 127.7, 125.9, 125.5, 125.2, 125.0, 120.6, 120.5, 60.25, 60.2, 58.5, 58.3, 44.9 (q, *J* = 29.7 Hz), 44.3 (q, *J* = 29.7 Hz), 43.0, 42.95, 37.5, 36.8, 36.0, 35.6, 28.2, 28.0. **HRMS** (ESI): calcd for $C_{26}H_{25}F_3N_3O_5S_2^+$ $[M+H]^+$ 580.1182, found: 580.1179. **M.p.**: 140 – 142 ˚C. Purity: >98% (UV 256 nm).

(6R,7R)-3-(((4-((4-carboxyphenyl)amino)-1,1,1-trifluoro-4-oxobutan-2-yl)thio)methyl)-8-oxo-7-(2-phenylacetamido)-5-thia-1-azabicyclo[4.2.0]oct-2-ene-2-carboxylic acid **(6)**.

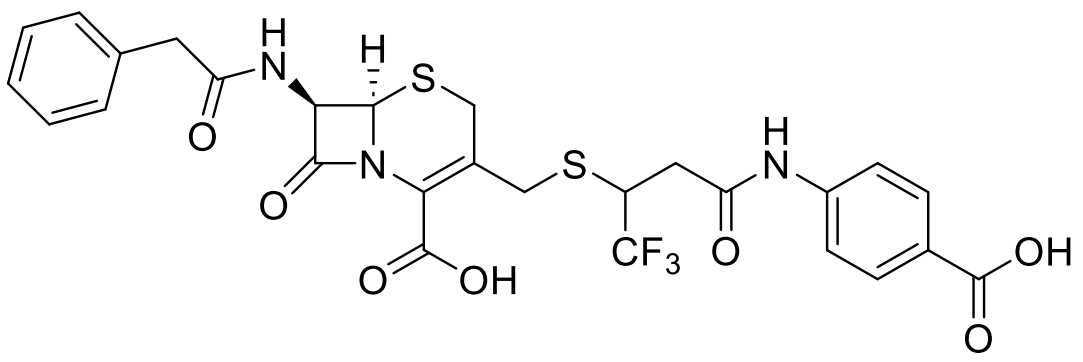

Synthesized according to the *General Procedure* using 7-ACA (150 mg, 0.55 mmol, 1.0 eq), 4-(4,4,4-trifluoro-3-mercaptobutanamido)benzoic acid (242 mg, 0.83 mmol, 1.5 eq), boron trifluoride ethyl etherate (208 µl, 1.65 mmol, 3.0 eq), MeCN (4 mL) and phenylacetyl chloride (283 µl, 2.14 mmol, 4.0 eq). The crude was purified using H_2O/MeCN (0.1% TFA) to yield **6** (118 mg, 0.19 mmol, 34%, over two steps) as a white solid. **^{1}H NMR** (400 MHz, DMSO-*d6*) δ 10.48, 10.44 (s, 1H), 9.19 – 9.03 (m, 1H), 7.96 – 7.87 (m, 2H), 7.76 – 7.64 (m, 2H), 7.38 – 7.16 (m, *overlapping water peak*, 5H), 5.71, 5.63 (dd, *J* = 8.3, 4.7 Hz, 1H), 5.13, 5.03 (d, *J* = 4.8 Hz, 1H), 4.11 – 3.77 (m, 2H), 3.72 – 3.42 (m, 5H), 3.08 – 2.90 (m, 1H), 2.77 – 2.57 (m, 1H). **^{13}C NMR** (101 MHz, DMSO-*d6*) δ 171.0, 167.0, 166.9, 166.85, 164.5, 163.05, 163.0, 142.75, 142.65, 135.9, 135.8, 130.6, 130.5, 129.1, 128.3, 127.1 (q, *J* = 277.6 Hz), 126.5, 126.2, 125.8, 125.6, 125.5, 125.4, 124.1, 118.6, 118.4, 59.05, 59.0, 57.7, 57.5, 43.8 (q, *J* = 29.1 Hz), 42.7 (q, *J* = 29.1 Hz), 41.6, 36.4, 35.6, 34.7, 26.8, 26.5. **HRMS** (ESI): calcd for $C_{27}H_{23}F_3N_3O_7S_2^-$ $[M-H]^-$ 622.0935, found: 622.0932. **M.p.**: 137 – 140 ˚C. Purity: 96% (UV 256 nm).

(6R,7R)-3-((((S)-2-((S)-2-carboxypyrrolidine-1-carbonyl)-3,3,3-trifluoropropyl)thio)methyl)-8-oxo-7-(2-phenylacetamido)-5-thia-1-azabicyclo[4.2.0]oct-2-ene-2-carboxylic acid **(7)**.

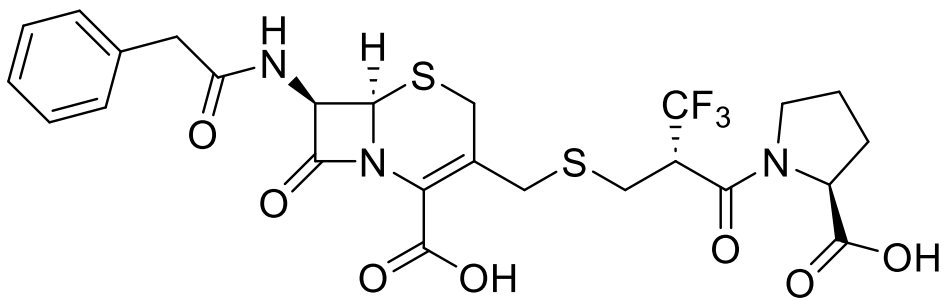

Synthesized according to the *General Procedure* using 7-ACA (100 mg, 0.37 mmol, 1.0 eq), ((*S*)-3,3,3-trifluoro-2-(mercaptomethyl)propanoyl)-L-proline (149 mg, 0.55 mmol, 1.5 eq), boron trifluoride ethyl etherate (138 µl, 1.10 mmol, 3.0 eq), MeCN (3 mL) and phenylacetyl chloride (175 µl, 1.32 mmol, 4.0 eq). The crude was purified using H_2O/MeCN (0.1% TFA) to yield **7** (47 mg, 0.08 mmol, 23%, over two steps) as a white solid. **^{1}H NMR** (400 MHz, DMSO-*d6*) (a 6:1 mixture of rotamers*) δ 9.20 – 9.07 (m, 1H), 7.35 – 7.18 (m, 5H), 5.65 (dd, *J* = 8.4, 4.7 Hz, 1H), 5.11 (d, *J* = 4.8 Hz, 1H), [4.75 – 4.69], 4.33 – 4.23 (m, 1H), 4.19 – 3.93 (m, 2H), 3.81 – 3.11 (m, *overlapping water peak*, 7H), 2.95 – 2.78 (m, 2H), 2.25 – 2.08 (m, 1H), 2.00 – 1.82 (m, 3H). **^{13}C NMR** (101 MHz, DMSO-*d6*) δ [173.2], 173.15, [171.85], 171.8, 165.1, [164.8], 164.0, 163.6, [163.5], 136.1, 129.4, 129.1, 128.7, [128.5], 127.0, 125.4, 125.0 (q, *J* = 282.4 Hz), [60.0], 59.5, 59.3, 58.3, 48.0, [47.7 (q, *J* = 24.8 Hz)], 47.6 (q, *J* = 24.8 Hz), [46.9], 42.0, [33.6], 33.0, [30.9], 29.3, [28.2], 27.0, 26.8, 24.6, [22.4]. **HRMS** (ESI calcd for $C_{25}H_{25}F_3N_3O_7S_2^-$ [M-H]$^-$ 600.1092, found: 600.1089. **M.p.**: 160 – 162 ˚C. Purity: >98% (UV 256 nm).

(6R,7R)-3-((((R)-2-((R)-2-carboxypyrrolidine-1-carbonyl)-3,3,3-trifluoropropyl)thio)methyl)-8-oxo-7-(2-phenylacetamido)-5-thia-1-azabicyclo[4.2.0]oct-2-ene-2-carboxylic acid **(8)**.

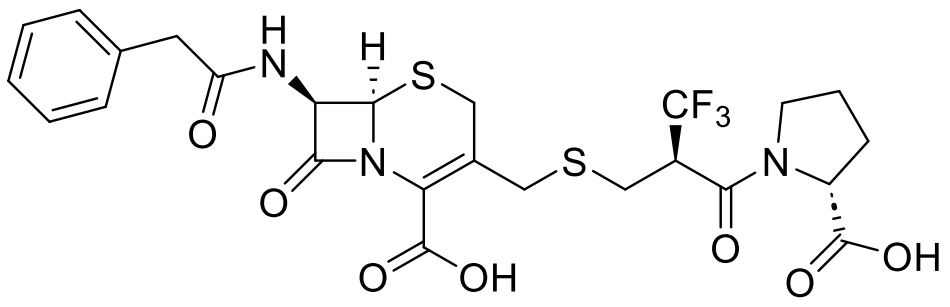

Synthesized according to the *General Procedure* using 7-ACA (193 mg, 0.71 mmol, 1.0 eq), ((*R*)-3,3,3-trifluoro-2-(mercaptomethyl)propanoyl)-D-proline (289 mg, 1.07 mmol, 1.5 eq), boron trifluoride ethyl etherate (268 µl, 2.13 mmol, 3.0 eq), MeCN (4 mL) and phenylacetyl chloride (186 µl, 1.41 mmol, 2.0 eq). The crude was purified using H_2O/MeCN (0.1% TFA) to yield **8** (104 mg, 0.17 mmol, 24%, over two steps) as a white solid. **^{1}H NMR** (400 MHz, DMSO-*d6*) (a 6:1 mixture of rotamers*) δ 9.19 – 8.99 (m, 1H), 7.34 – 7.18 (m, 5H), 5.70 – 5.54 (m, 1H), 5.21 – 5.05 (m, 1H), [4.76 – 4.67], 4.34 – 4.23 (m, 1H), 4.15 – 3.81 (m, 1H), 3.78 – 3.10 (m, *overlapping water peak*, 8H), 3.03 – 2.81 (m, 2H), 2.23 – 2.11 (m, 1H), 2.01 – 1.82 (m, 3H). **^{13}C NMR** (101 MHz, DMSO-*d6*) δ [172.8], 172.7, 171.0, [170.95], [164.6], 164.5, [164.1 (q, *J* = 2.1 Hz)], 163.3 (q, *J* = 2.1 Hz), 163.2, [163.1], 135.9, 129.0, 128.4, 128.3, [127.4], 126.5, [125.1], 125.0, 124.6 (q, *J* = 281.8 Hz), [59.6], 59.1, 59.0, 57.9, 47.5, 47.3 (q, *J* = 24.5 Hz), [46.5], 41.6, [34.0], 33.3, [30.4], 28.9, 27.4, 26.9, 24.2, [22.1]. *TFA signals were observed but are not reported*. **HRMS** (ESI): calcd for $C_{25}H_{25}F_3N_3O_7S_2^-$ [M-H]$^-$ 600.1092, found: 600.1086. **M.p.**: 127 – 129 ˚C. Purity: >95% (UV 256 nm).

* Chemical shifts of minor rotamer are enclosed in square brackets.

Carbon assignment

(6R,7R)-3-(((2-(benzylcarbamoyl)-3,3-trifluoropropyl)thio)methyl)-8-oxo-7-(2-phenylacetamido)-5-thia-1-azabicyclo[4.2.0]oct-2-ene-2-carboxylic acid **(3)**.

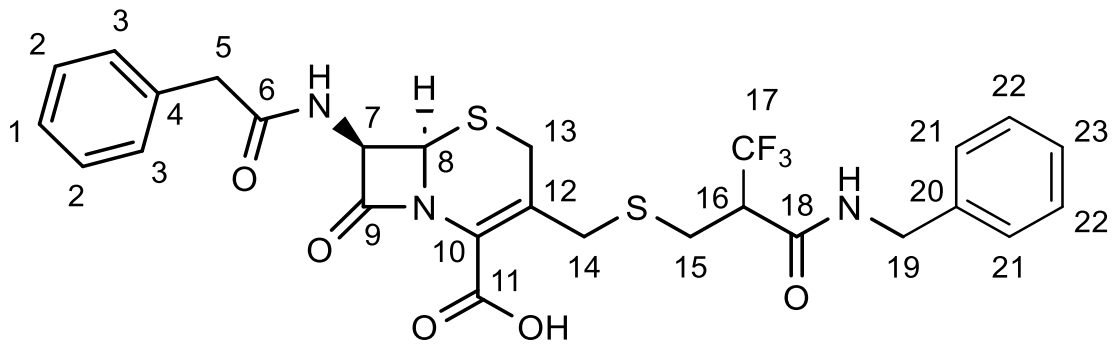

Carbon №	Shift (ppm)
1	126.5
2	128.3
3	129.1
4	135.9, 135.85
5	41.6
6	171.0
7	59.0, 58.95
8	58.0
9	164.8, 164.6
10	127.9, 127.5 or 125.2, 124.9
11	163.1, 163.0
12	127.9, 127.5 or 125.2, 124.9
13	27.1, 26.7
14	33.1, 32.6
15	26.7, 26.1
16	49.8, 49.6 (q, *J* = 24.6 Hz)
17	124.8 (q, *J* = 281.9 Hz)
18	164.6, 164.4
19	42.5
20	138.6, 138.5
21	127.2
22	128.4, 128.35
23	127.05, 127.0

(6R,7R)-8-oxo-7-(2-phenylacetamido)-3-(((3,3,3-trifluoro-2-(phenylcarbamoyl)propyl)thio)methyl)-5-thia-1-azabicyclo[4.2.0]oct-2-ene-2-carboxylic acid **(4)**.

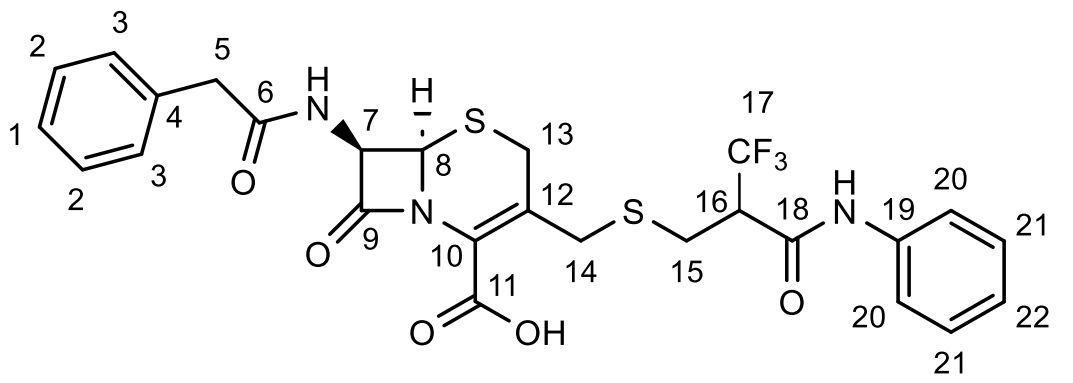

Carbon №	Shift (ppm)
1	126.5
2	128.2
3	129.0
4	135.85, 135.8
5	41.65, 41.6
6	171.0, 170.95
7	59.0
8	58.0
9	164.8, 164.7
10	128.0, 127.4 or 125.1, 124.9
11	163.05, 163.0
12	128.0, 127.4 or 125.1, 124.9
13	27.1, 26.7
14	33.1, 32.4
15	26.7, 26.0
16	50.6, 50.4 (q, *J* = 24.4 Hz)
17	124.7 (q, *J* = 281.5 Hz)
18	163.3, 163.1
19	138.2, 138.1
20	119.5, 119.4
21	128.95, 128.9
22	124.3, 124.2

(6R,7R)-8-oxo-7-(2-phenylacetamido)-3-(((1,1,1-trifluoro-4-oxo-4-(phenylamino)butan-2-yl)thio)methyl)-5-thia-1-azabicyclo[4.2.0]oct-2-ene-2-carboxylic acid **(5)**.

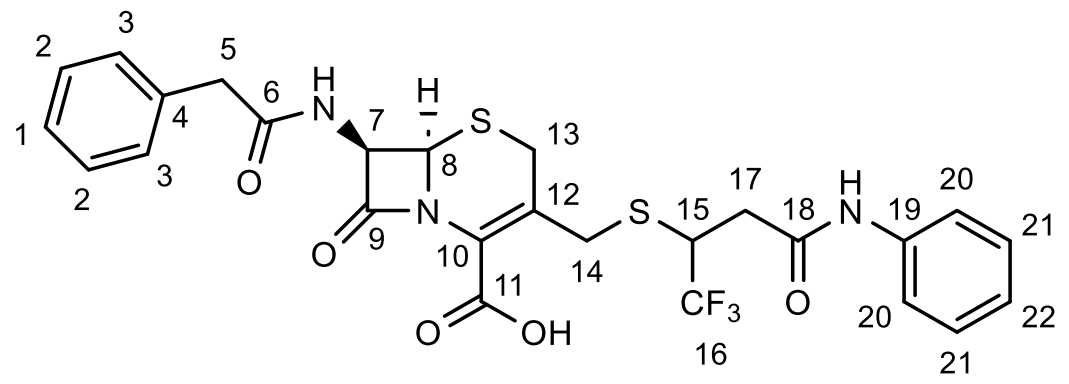

Carbon №	Shift (ppm)
1	127.8, 127.7
2	129.4
3	130.1
4	136.3
5	43.0, 42.95
6	172.4, 172.35
7	60.25, 60.2
8	58.5, 58.3
9	165.9, 165.7
10	125.9, 125.5
11	163.4, 163.3
12	129.9, 129.8
13	28.2, 28.0
14	36.0, 35.6
15	44.9, 44.3(q, *J* = 29.7 Hz)
16	128.1 (q, *J* = 275.8 Hz)
17	37.5, 36.8
18	167.6, 167.5
19	139.4, 139.3
20	120.6, 120.5
21	129.9, 129.85
22	125.2, 125.0

(6R,7R)-3-(((4-((4-carboxyphenyl)amino)-1,1,1-trifluoro-4-oxobutan-2-yl)thio)methyl)-8-oxo-7-(2-phenylacetamido)-5-thia-1-azabicyclo[4.2.0]oct-2-ene-2-carboxylic acid **(6)**.

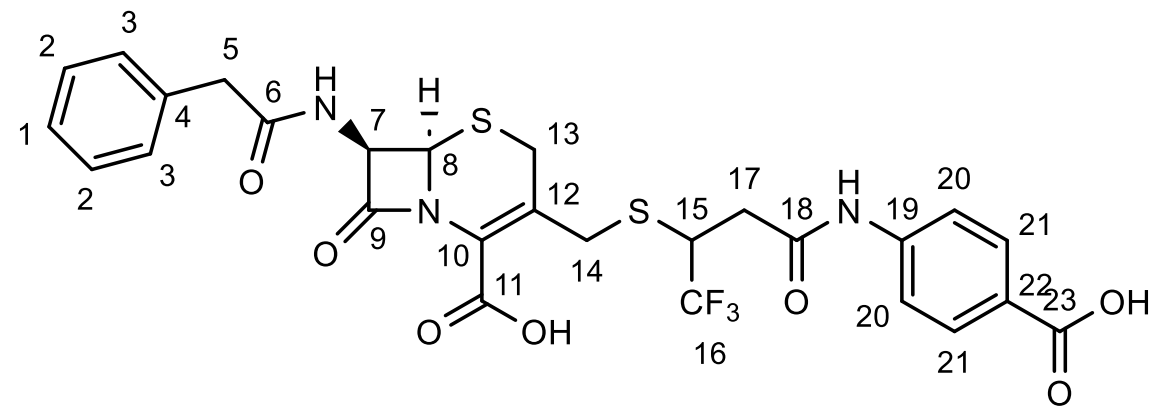

Carbon №	Shift (ppm)
1	126.5
2	128.3
3	129.1
4	135.9, 135.8
5	41.6
6	171.0
7	59.05, 59.0
8	57.7, 57.5
9	164.5
10	126.2, 125.8 or 125.5, 124.1
11	163.05, 163.0
12	126.2, 125.8 or 125.5, 124.1
13	26.8, 26.5
14	34.7
15	43.8, 42.7 (q, J = 29.1 Hz)
16	127.1 (q, J = 277.6 Hz)
17	36.4, 35.6
18	167.0 or 166.9, 166.85
19	125.6, 125.4
20	118.6, 118.4
21	130.6, 130.5
22	142.75, 142.65
23	167.0 or 166.9, 166.85

(6R,7R)-3-((((S)-2-((S)-2-carboxypyrrolidine-1-carbonyl)-3,3,3-trifluoropropyl)thio)methyl)-8-oxo-7-(2-phenylacetamido)-5-thia-1-azabicyclo[4.2.0]oct-2-ene-2-carboxylic acid **(7)**.

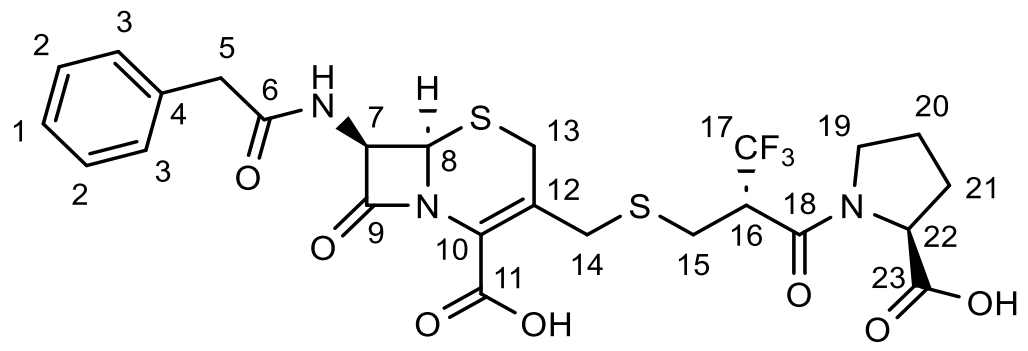

Carbon №	Shift (ppm)
1	127.0
2	128.7
3	129.4
4	136.1
5	42.0
6	171.85, 171.8
7	59.3
8	58.3
9	165.1
10	129.1, 128.5 or 125.4
11	163.6, 163.5
12	129.1, 128.5 or 125.4
13	26.8
14	33.6, 33.0
15	28.2, 27.0
16	47.7, 47.6 (q, J = 24.8 Hz)
17	125.0 (q, J = 282.4 Hz)
18	164.8, 164.0
19	48.0, 46.9
20	24.6, 22.4
21	30.9, 29.3
22	60.0, 59.5
23	173.2, 173.15

(6R,7R)-3-((((R)-2-((R)-2-carboxypyrrolidine-1-carbonyl)-3,3,3-trifluoropropyl)thio)methyl)-8-oxo-7-(2-phenylacetamido)-5-thia-1-azabicyclo[4.2.0]oct-2-ene-2-carboxylic acid **(8)**.

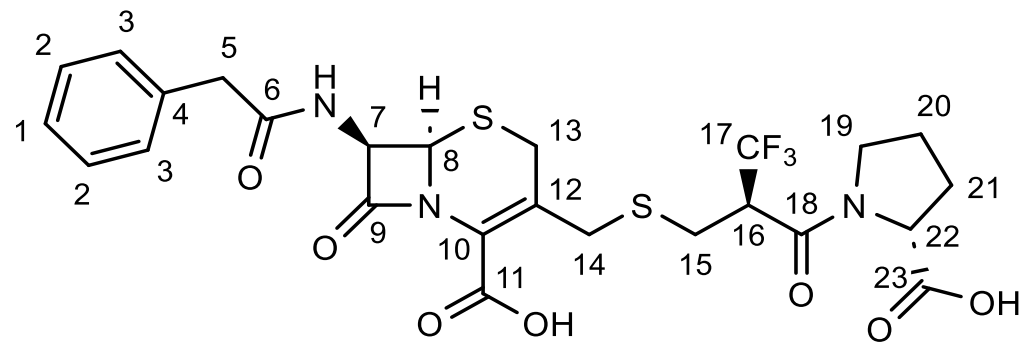

Carbon №	Shift (ppm)
1	126.5
2	128.3
3	129.0
4	135.9
5	41.6
6	171.0, 170.95
7	59.1
8	57.9
9	164.6, 164.5
10	128.4, 127.4 or 125.1, 125.0
11	163.2, 163.1
12	128.4, 127.4 or 125.1, 125.0
13	26.9
14	34.0, 33.3
15	27.4
16	47.3 (q, J = 24.5 Hz)
17	124.6 (q, J = 281.8 Hz)
18	164.1, 163.3 (q, J = 2.1 Hz)
19	47.5, 46.5
20	24.2, 22.1
21	30.4, 28.9
22	59.6, 59.0
23	172.8, 172.7

LC-MS-based hydrolysis experiments

Hydrolysis using basic conditions

The conjugates (6 µL, 0.174 M in DMSO) were added to 1 M $Na_2CO_{3(aq)}$ or 3 M $(NH_4)_2CO_{3(aq)}$. At the indicated time intervals, samples were taken from the reaction mixture and diluted in water or methanol (1:1 v/v) and analyzed following the general procedure for UHPLC-HRMS (Fig. S1–S9).

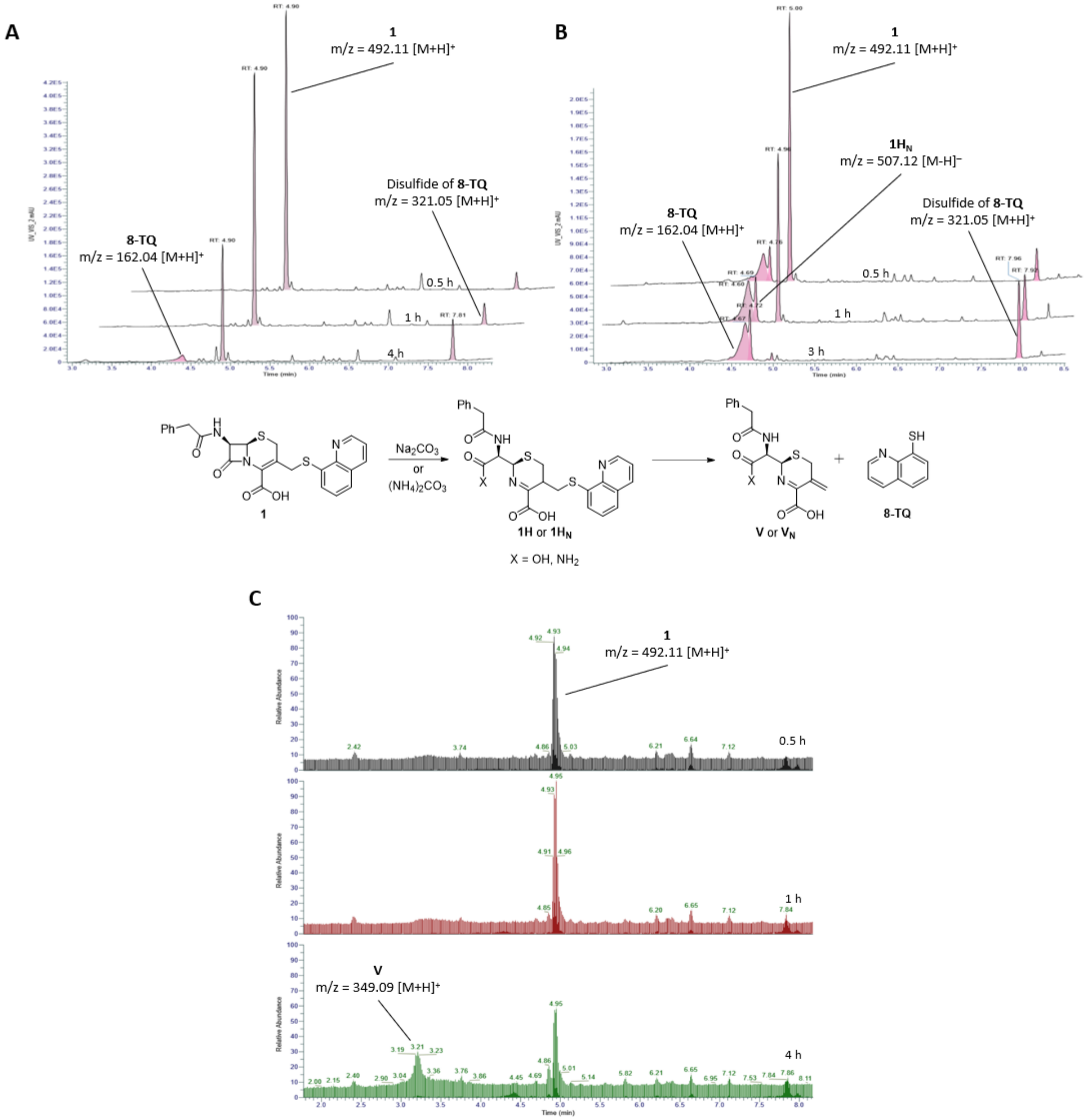

Figure S1. LC-MS analysis of Na_2CO_3 (A and C) and $(NH_4)_2CO_3$-mediated (B) degradation of **1**. A, B – UV traces; C – TIC.

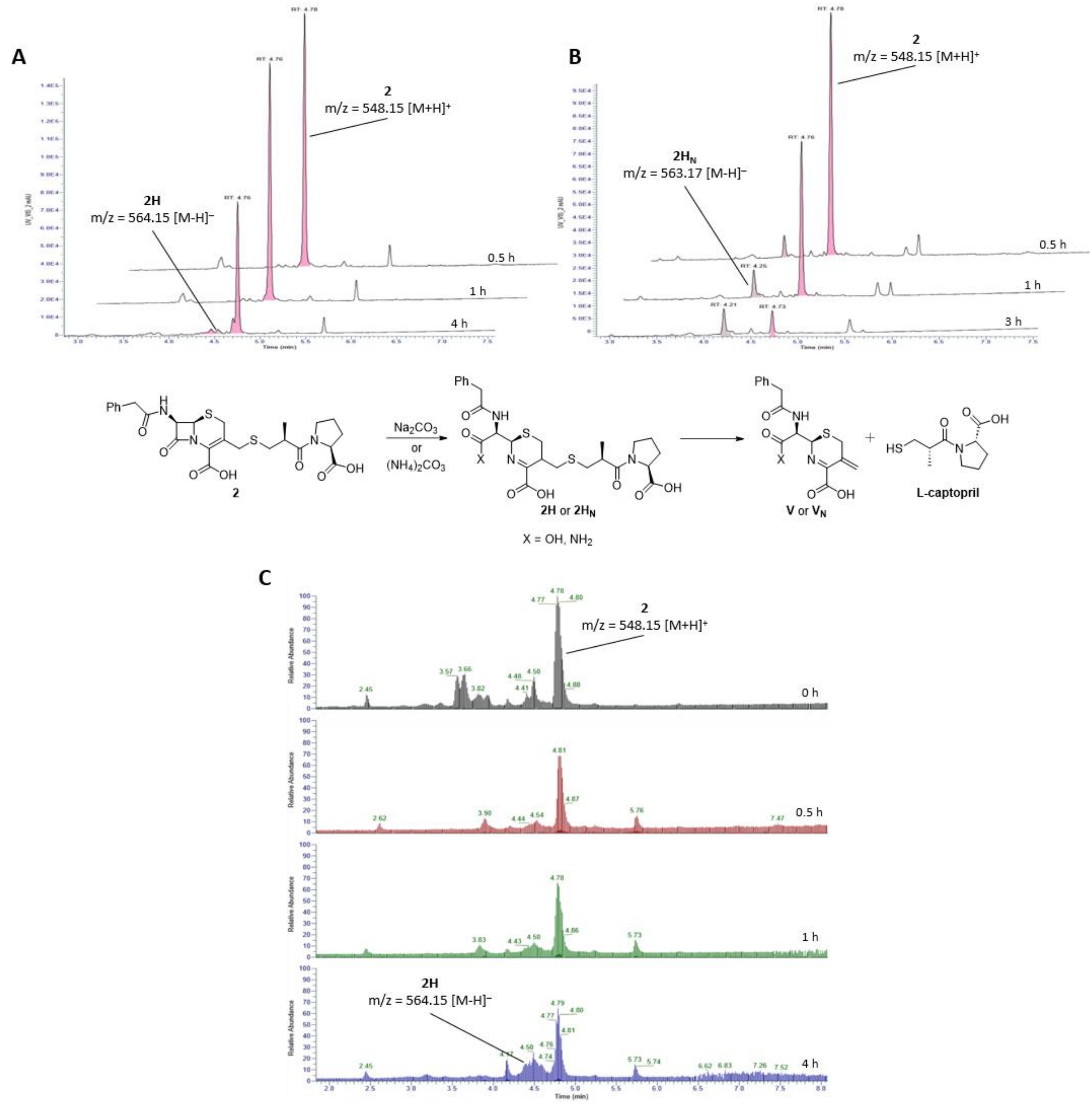

Figure S2. LC-MS analysis of Na_2CO_3 (A and C) and $(NH_4)_2CO_3$-mediated (B) degradation of **2**. A, B – UV traces; C – TIC.

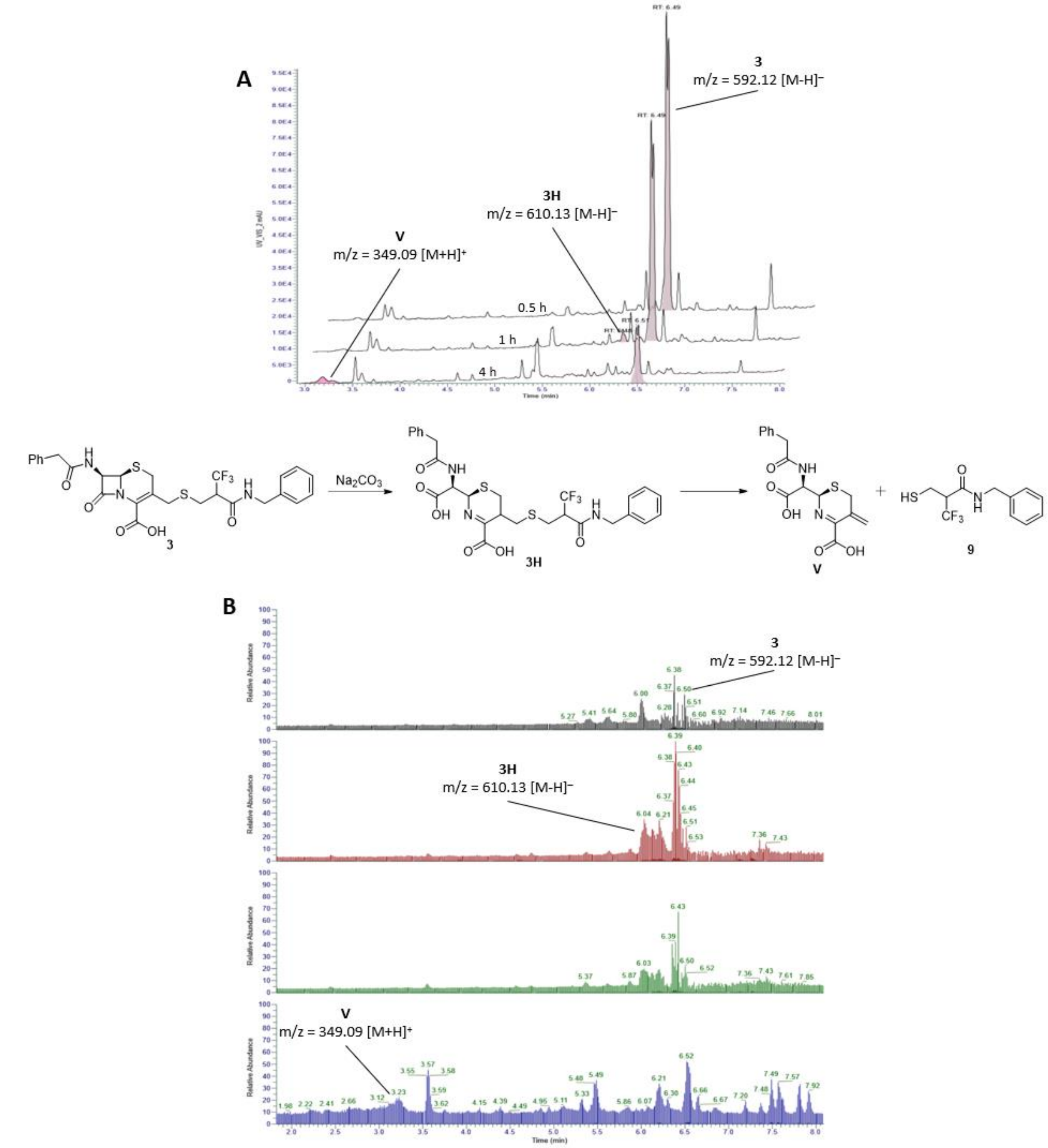

Figure S3. LC-MS analysis of Na_2CO_3-mediated degradation of **3**. A – UV traces; B – TIC.

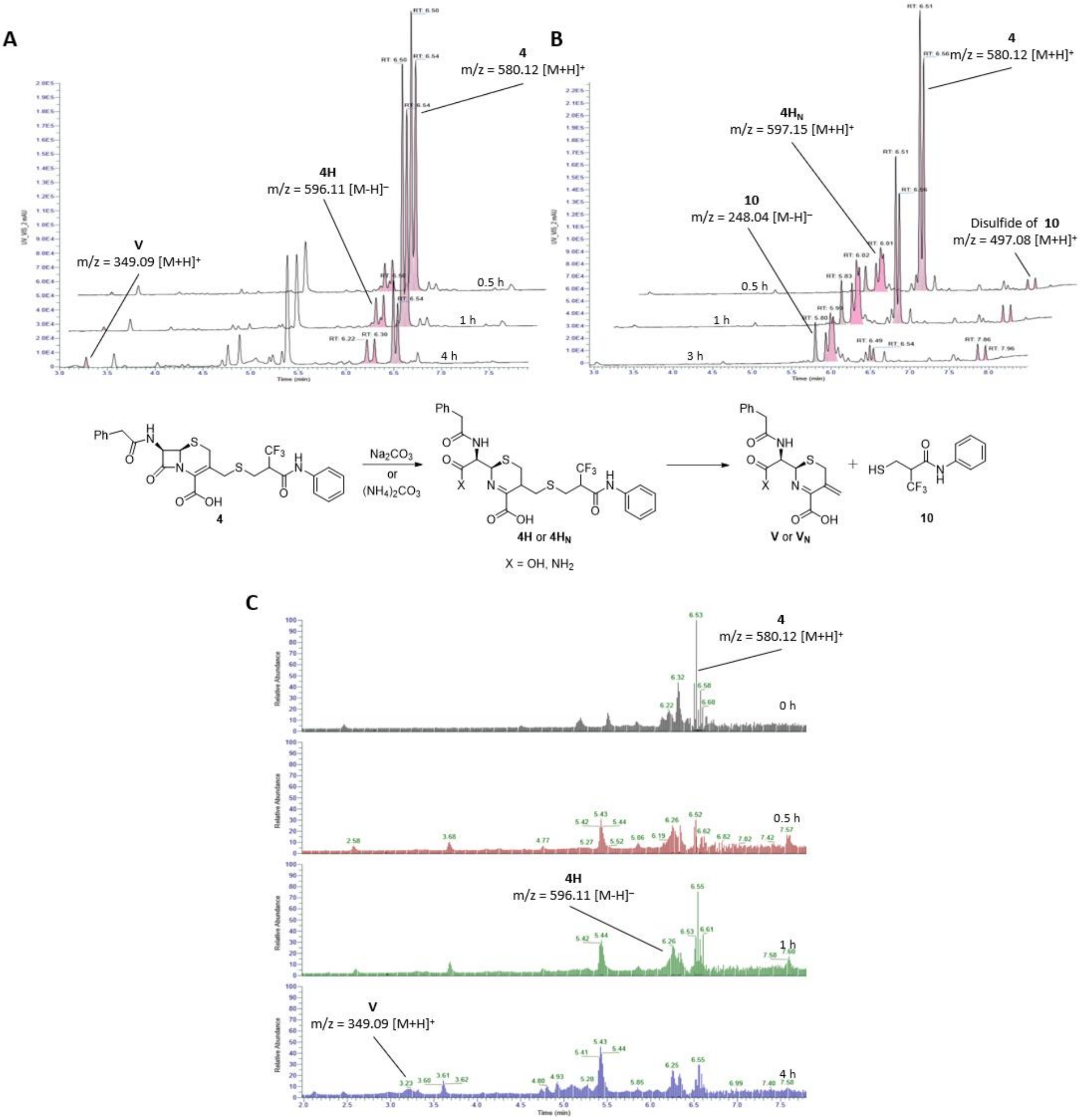

Figure S4. LC-MS analysis of Na_2CO_3 (A and C) and $(NH_4)_2CO_3$-mediated (B) degradation of **4**. A, B – UV traces; C – TIC.

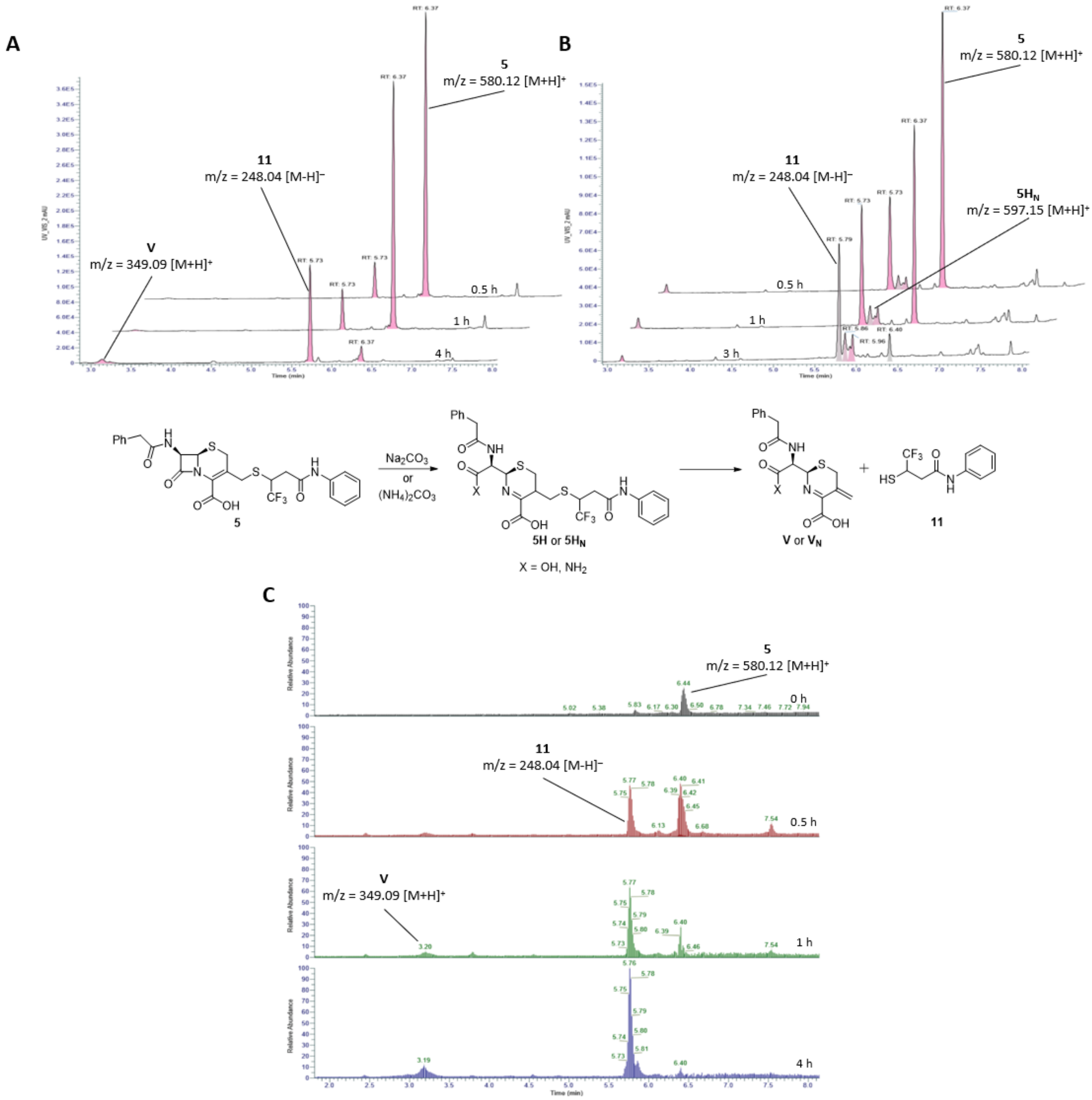

Figure S5. LC-MS analysis of Na_2CO_3 (A and C) and $(NH_4)_2CO_3$-mediated (B) degradation of **5**. A, B – UV traces; C – TIC.

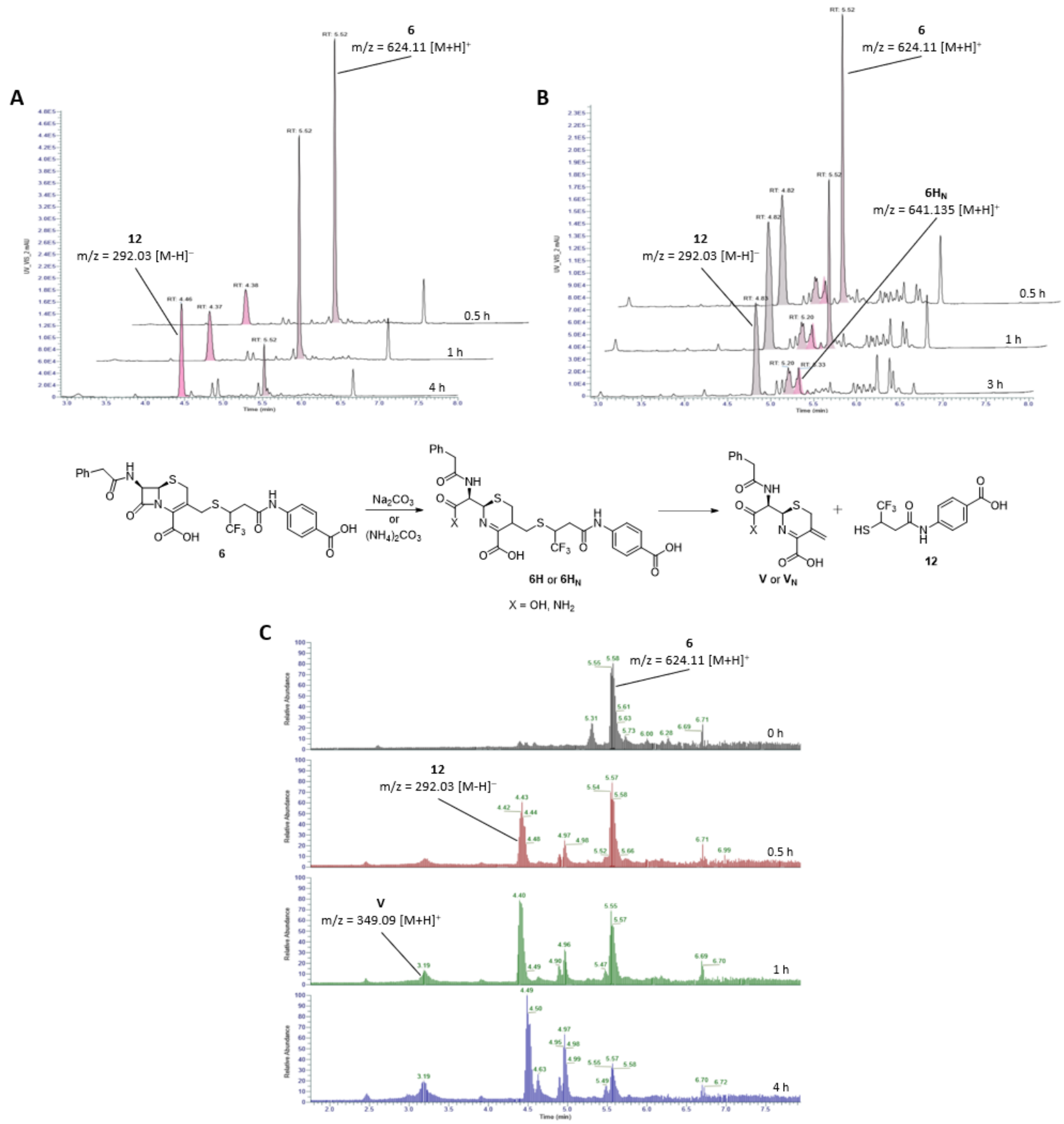

Figure S6. LC-MS analysis of Na_2CO_3 (A and C) and $(NH_4)_2CO_3$-mediated (B) degradation of **6**. A, B – UV traces; C – TIC.

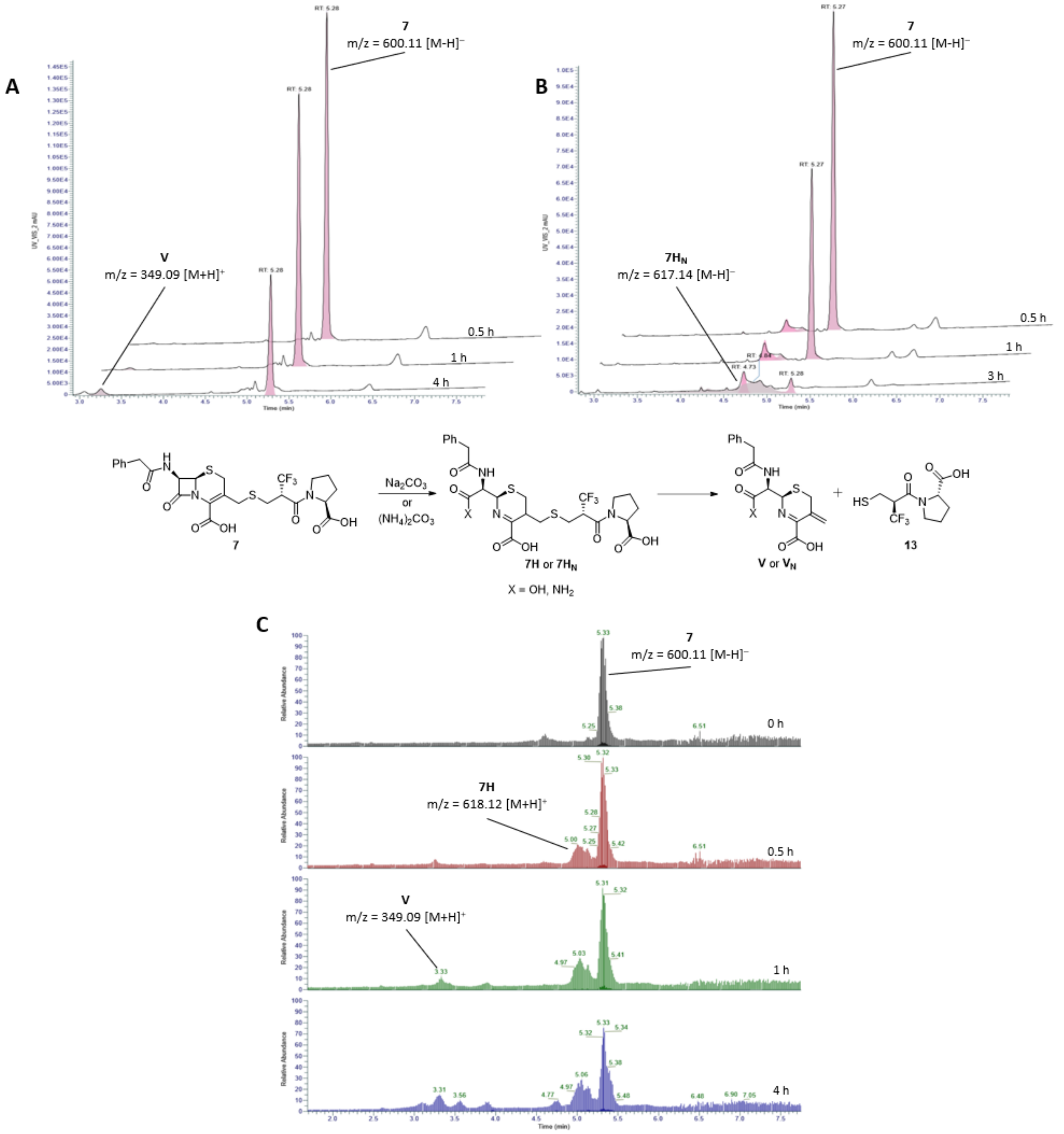

Figure S7. LC-MS analysis of Na_2CO_3 (A and C) and $(NH_4)_2CO_3$-mediated (B) degradation of **7**. A, B – UV traces; C – TIC.

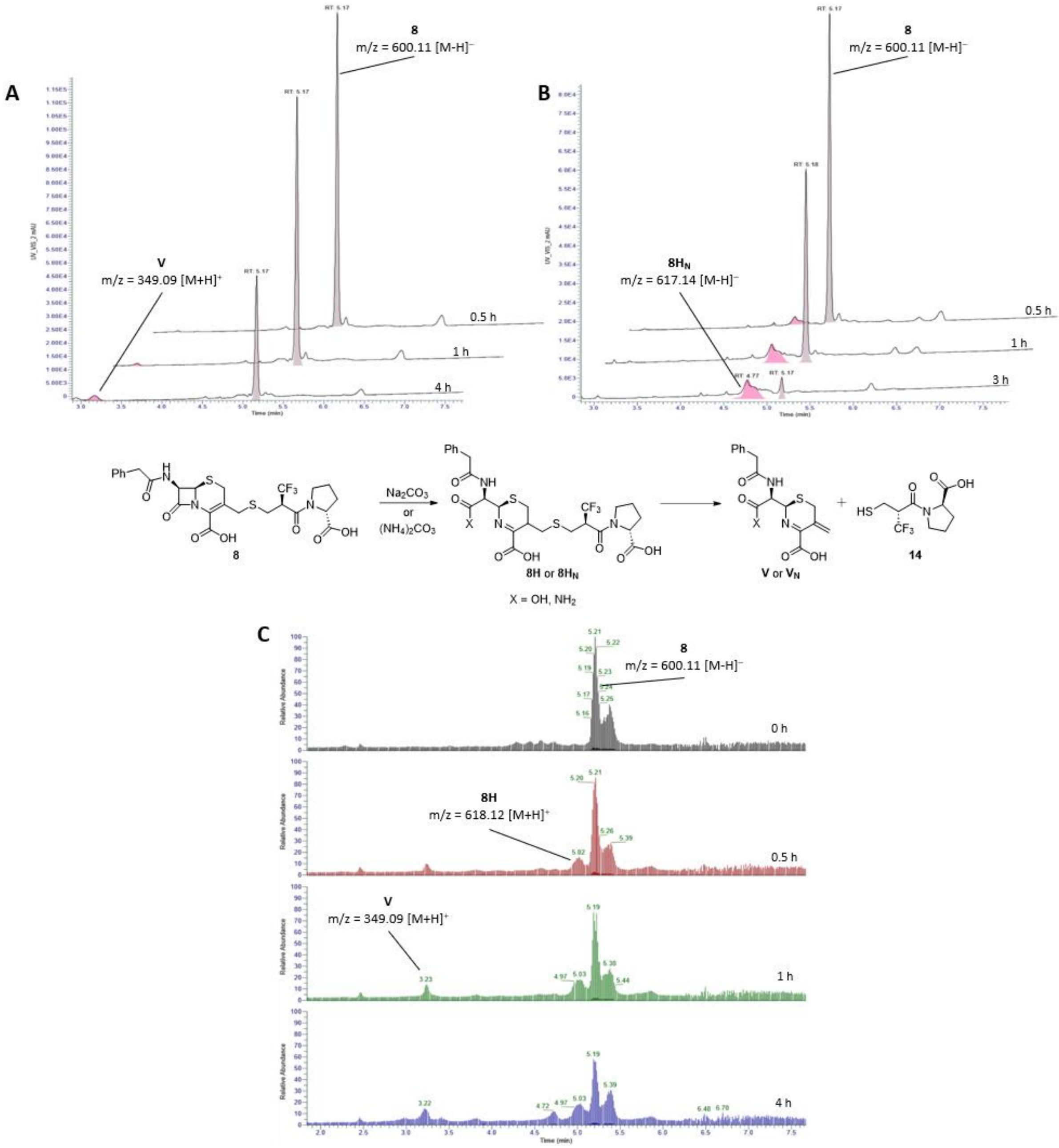

Figure S8. LC-MS analysis of Na_2CO_3 (A and C) and $(NH_4)_2CO_3$-mediated (B) degradation of **8**. A, B – UV traces; C – TIC.

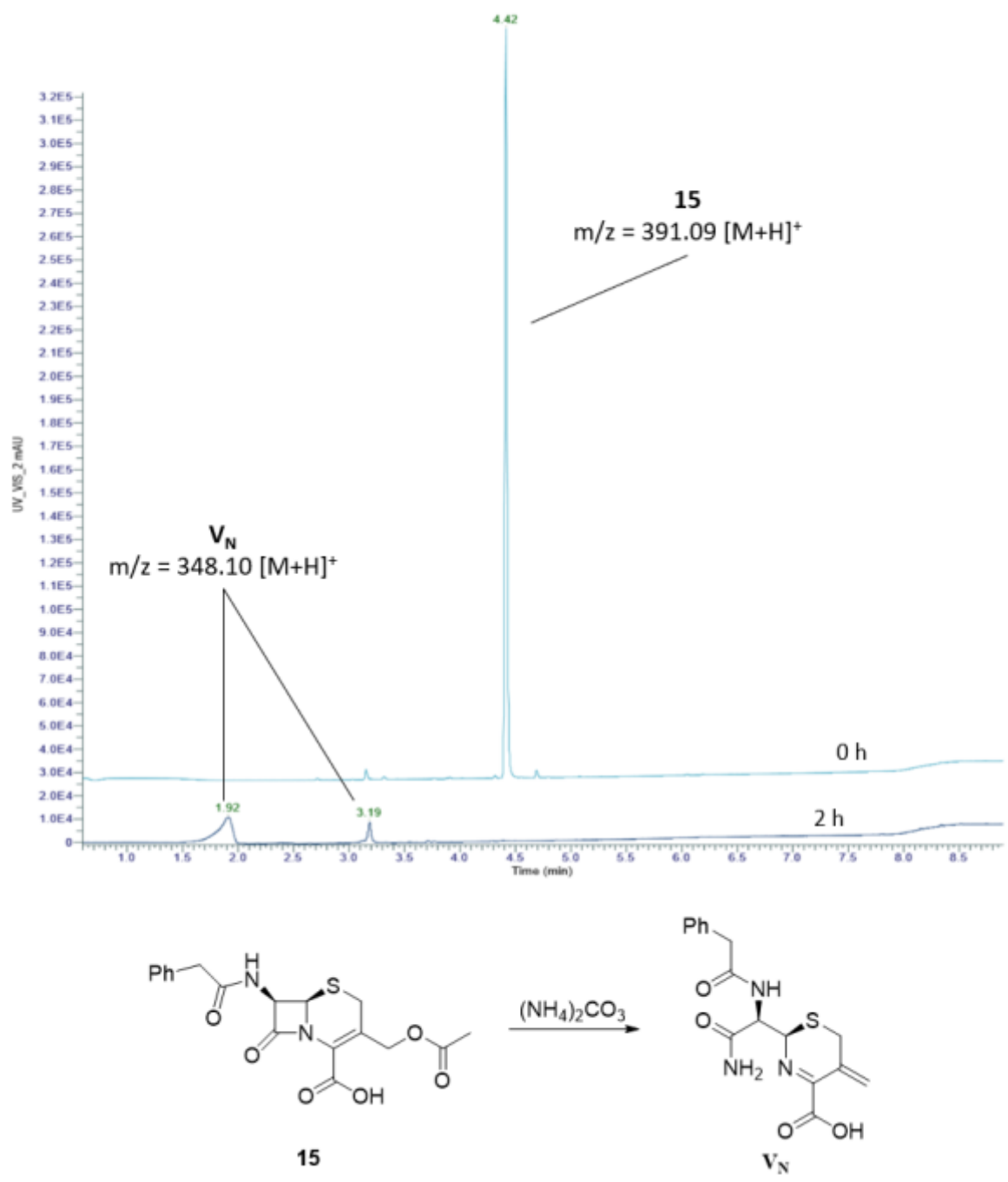

Figure S9. LC-MS analysis of $(NH_4)_2CO_3$-mediated degradation of **15**.

Enzymatic hydrolysis

Stock solutions of conjugates **4–6** in DMSO-*d6* were prepared (0.174 M). To a mixture of 586 µl 20 mM HEPES buffer (pH 7.4), 6 µl $ZnSO_4$ (50 mM in H_2O) and 6 µl compound **4–6** (stock solution: 0.174 M in DMSO-*d6*) 2 µl of NDM-1 (37.4 µM) were added. At various time-points after incubation at ambient temperature, samples were taken from the reaction mixture and diluted in acetonitrile (1:2 v/v). After centrifugation for 5 minutes at 6000 rpm, the supernatant was analyzed following the general procedure for UHPLC-HRMS.

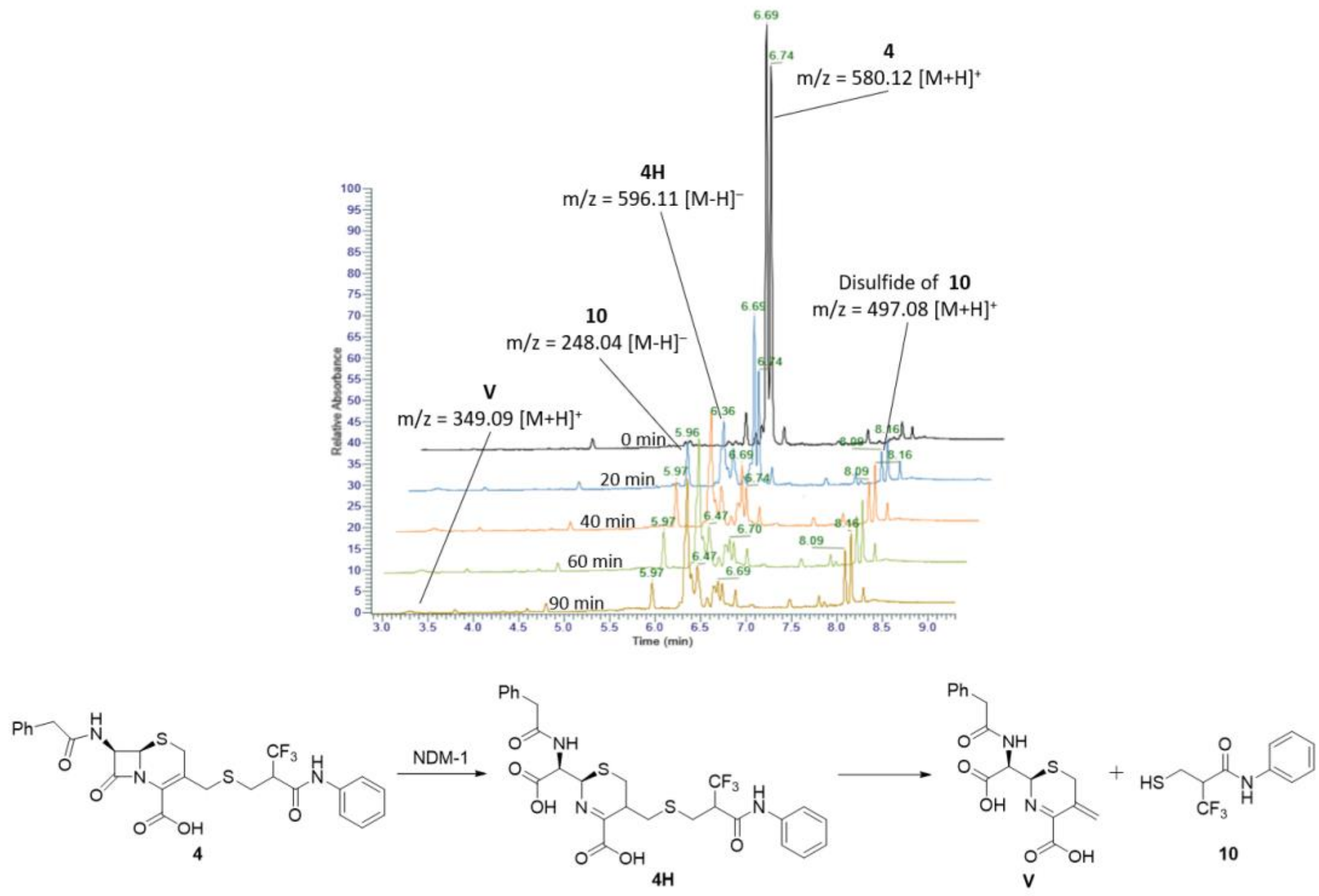

Figure S10. LC-MS analysis of NDM-1-mediated degradation of **4**.

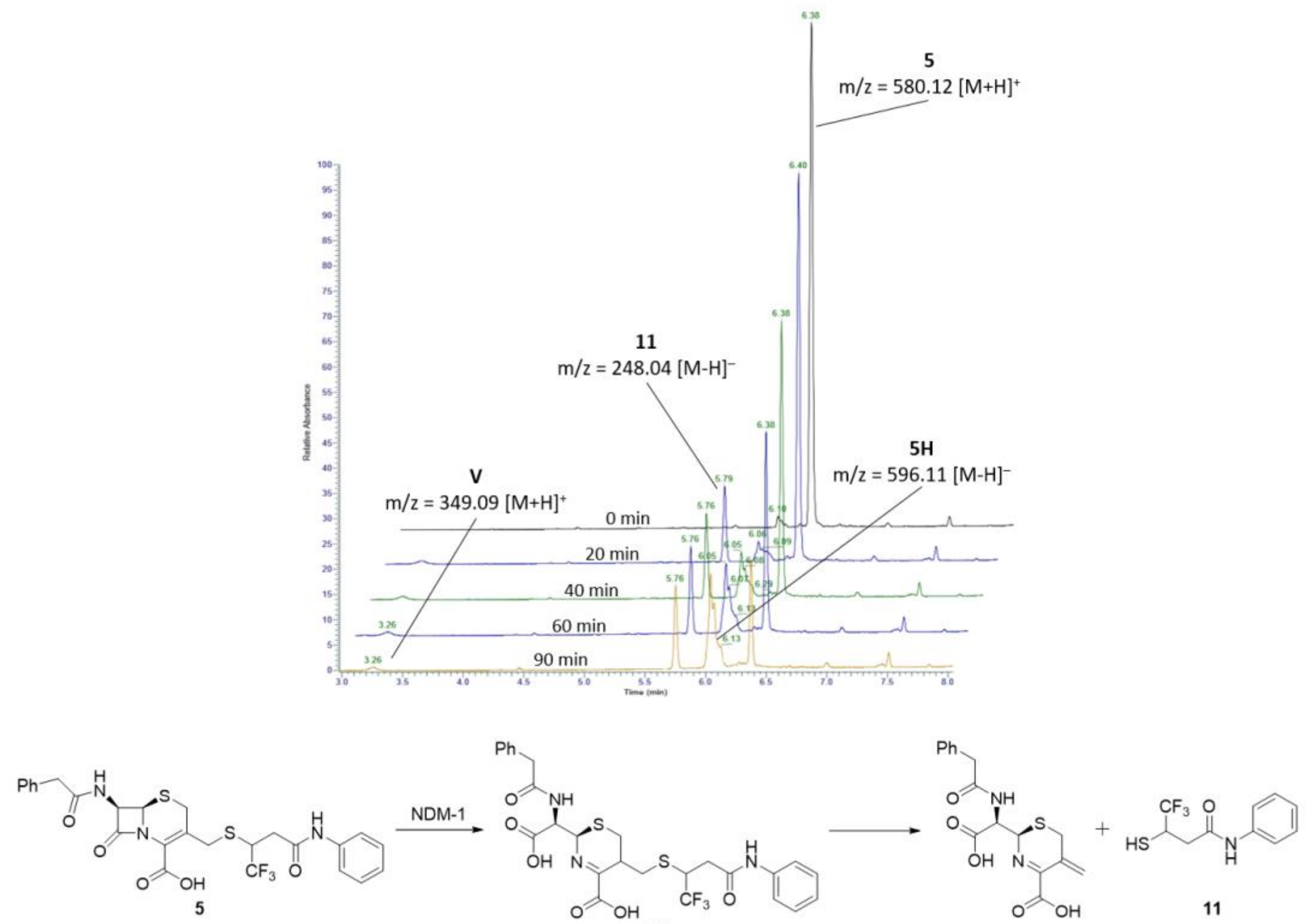

Figure S11. LC-MS analysis of NDM-1-mediated degradation of **5**.

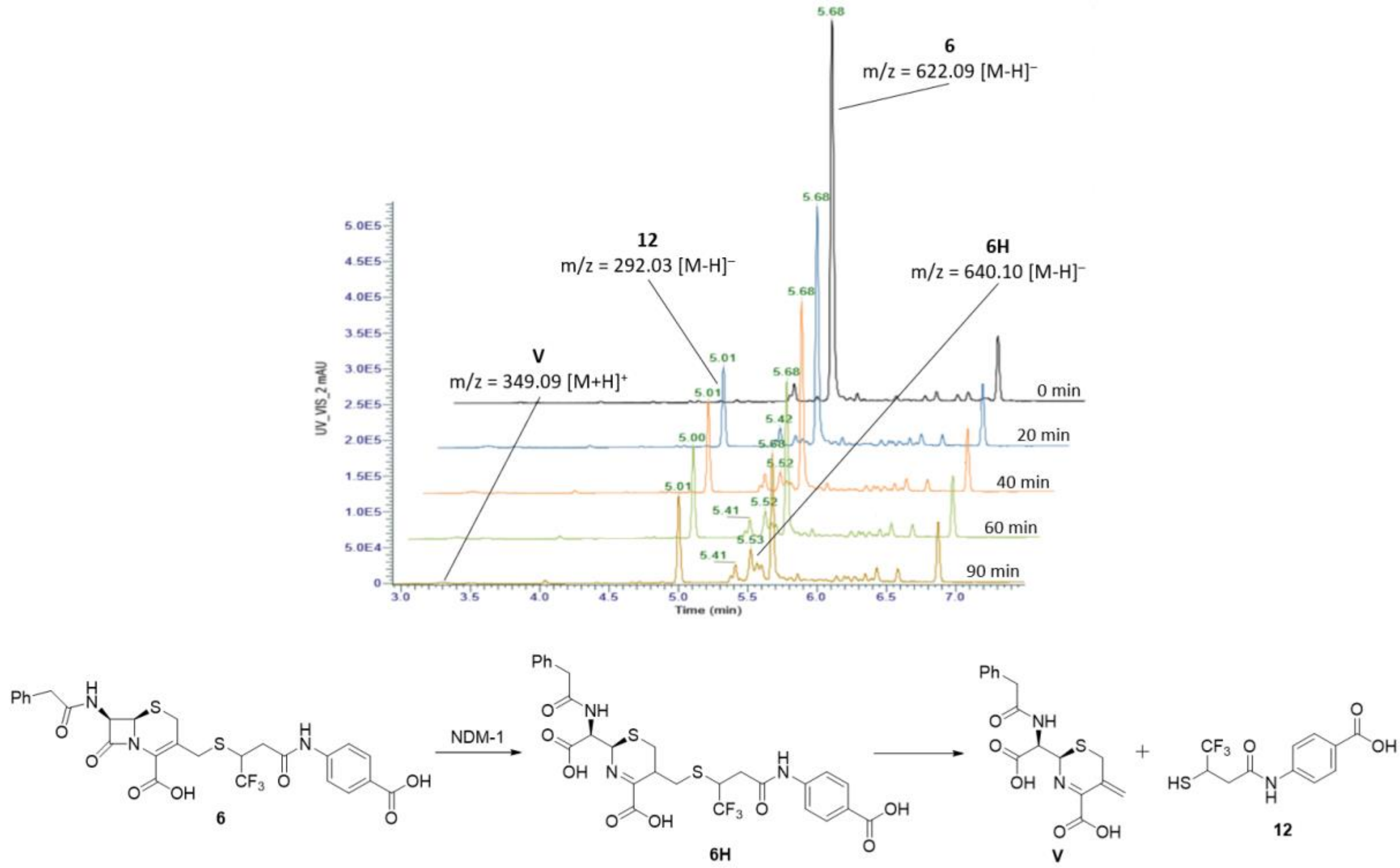

Figure S12. LC-MS analysis of NDM-1-mediated degradation of **6**.

NMR-based enzymatic hydrolysis experiments

1D ^{1}H NMR spectra were acquired with a fid size of 65k points over a 4 second acquisition time and a 4 second relaxation delay (D1) for 32 scans (4 dummy scans) for a total experiment time of 5 minutes per spectra. Excitation sculpting was utilized to remove the residual water peak at 4.7 ppm. 1D ^{19}F spectra were acquired with a fid size of 131k points with an acquisition time of 700 ms and a relaxation delay of 2 s for 64 scans with a total experiment time of 3 minutes.

Stock solutions of conjugates **1–8** in DMSO-*d6* were prepared (0.174 M). The NMR sample consisted of 582 µl HEPES buffer (20 mM in D_2O), 6 µl $ZnSO_4$ (50 mM in D_2O) and 6 µl compound **1–8** (stock solution: 0.174 M in DMSO-*d6*). An initial ^{1}H spectra was acquired before the addition of 2 µl NDM-1 (37.4 µM). After NDM-1 addition, ^{1}H spectra were acquired in a continuous manner over a period of 2 hours. Conjugate **1** was used as a reference compound to optimize the conditions of the hydrolysis experiment and the obtained data was found to be in accordance with literature reports.[2] Conjugate **3** displayed limited solubility in the conditions of the experiment, therefore, the results are not shown.

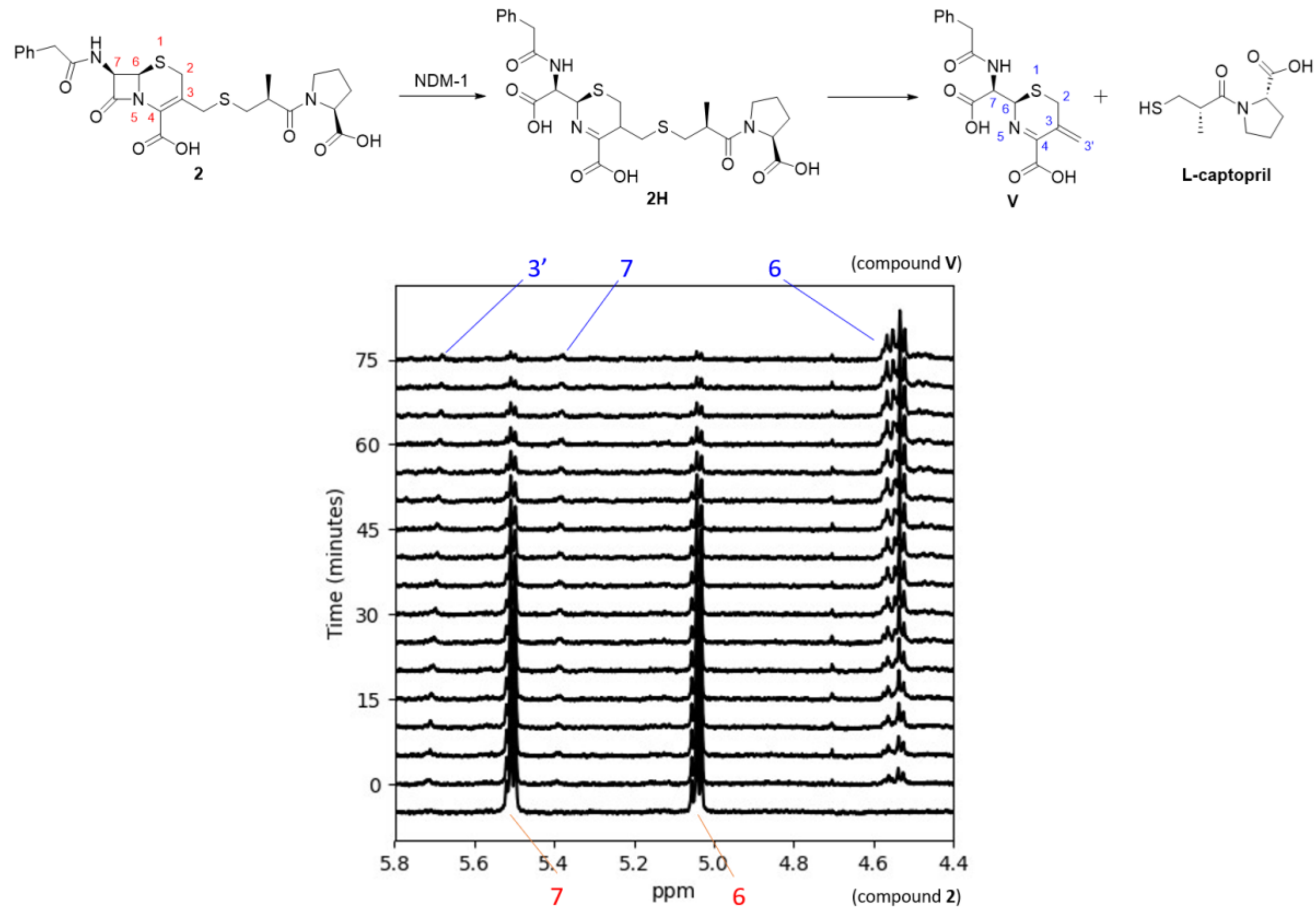

Figure S13. NMR analysis of NDM-1-mediated degradation of **2**.

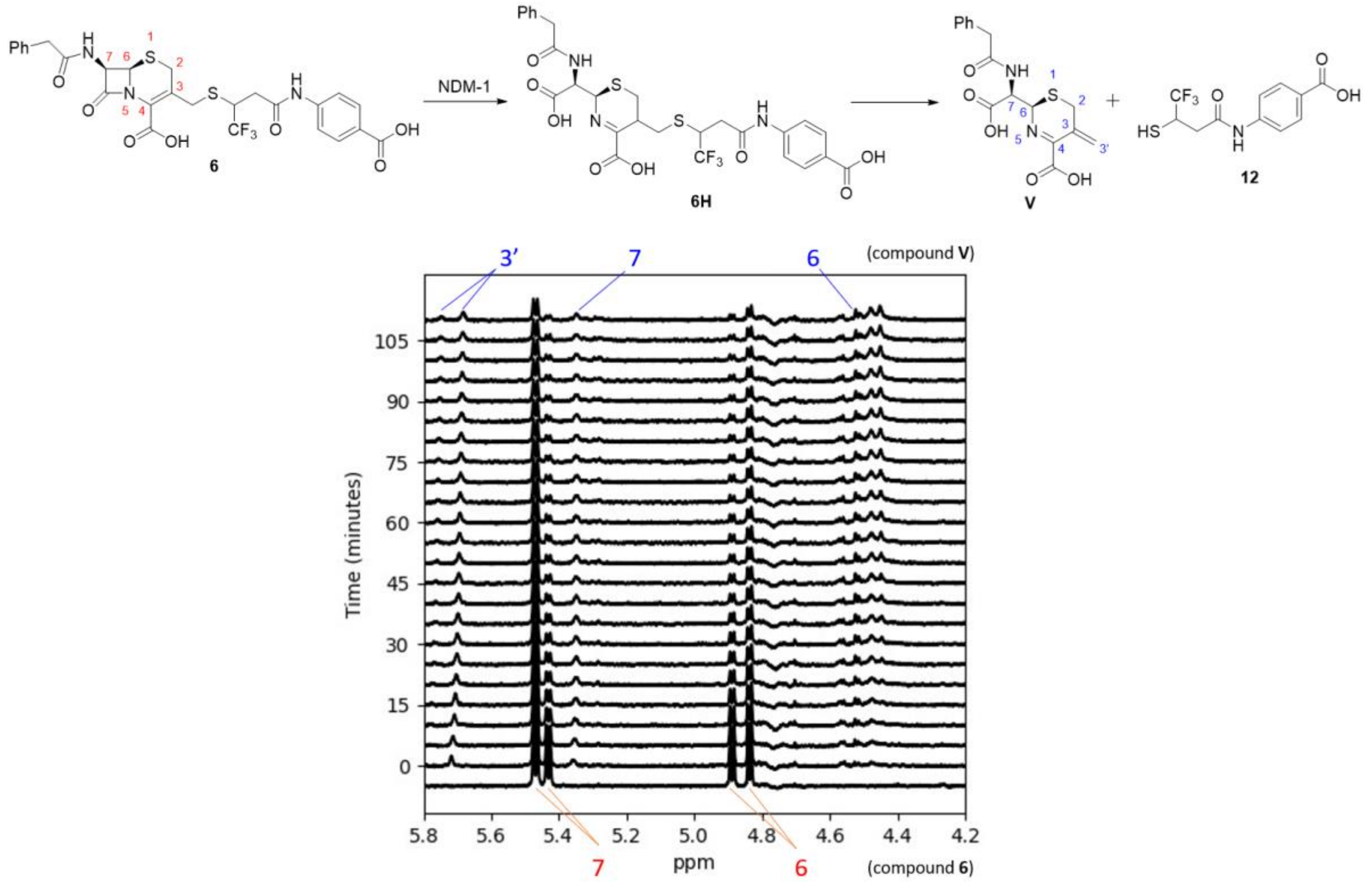

Figure S14. NMR analysis of NDM-1-mediated degradation of **6**.

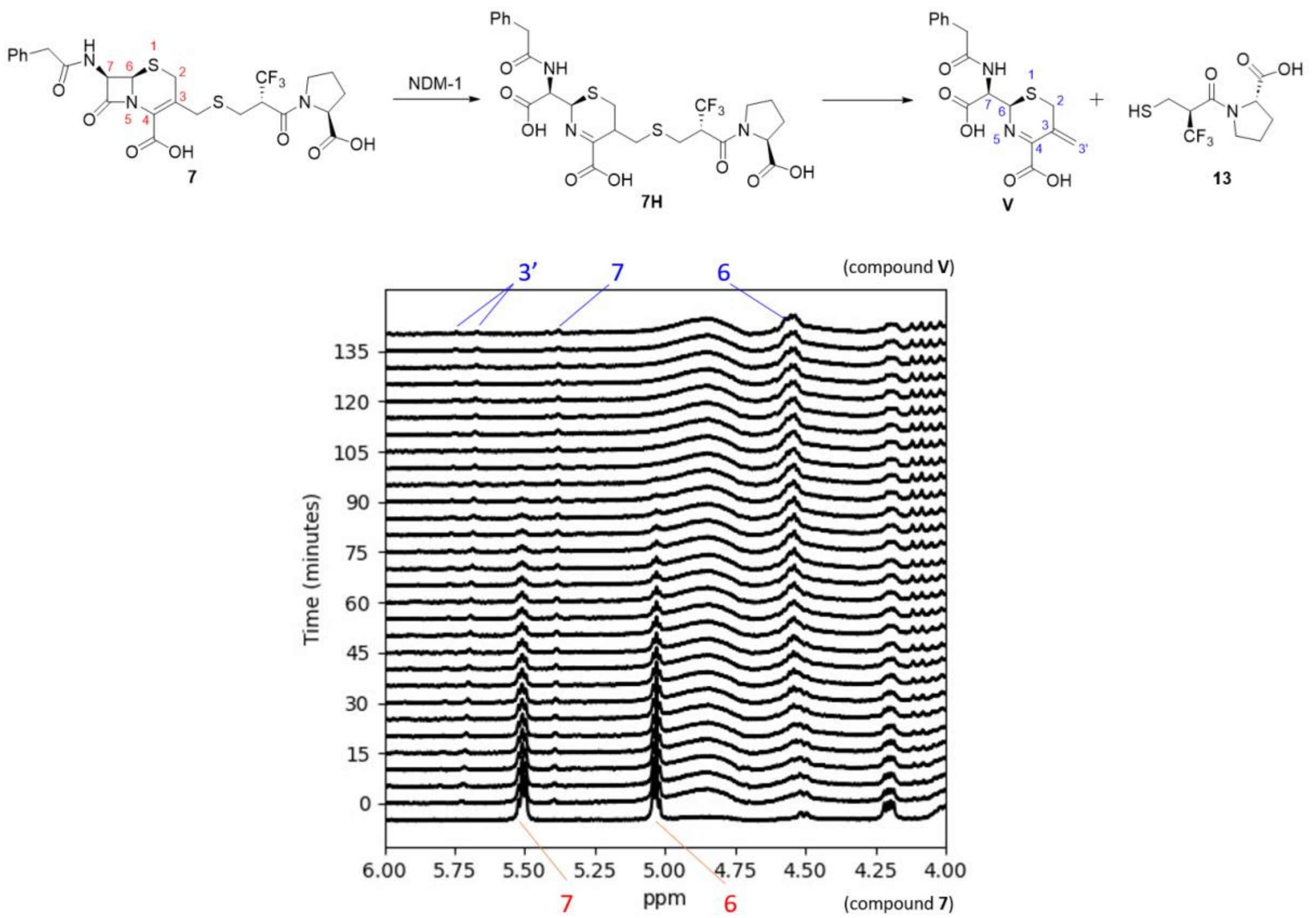

Figure S15. NMR analysis of NDM-1-mediated degradation of **7**.

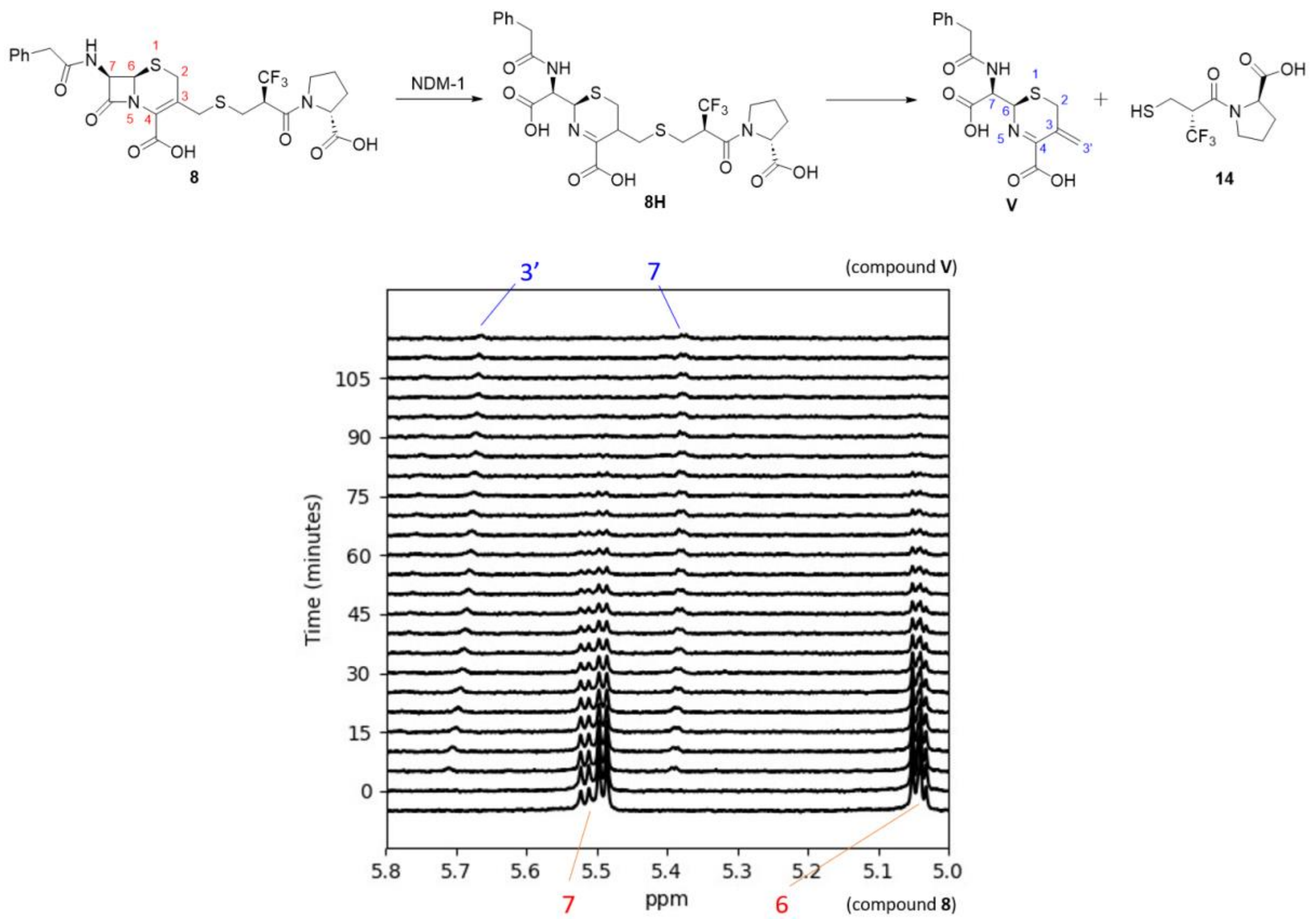

Figure S16. NMR analysis of NDM-1-mediated degradation of **8**.

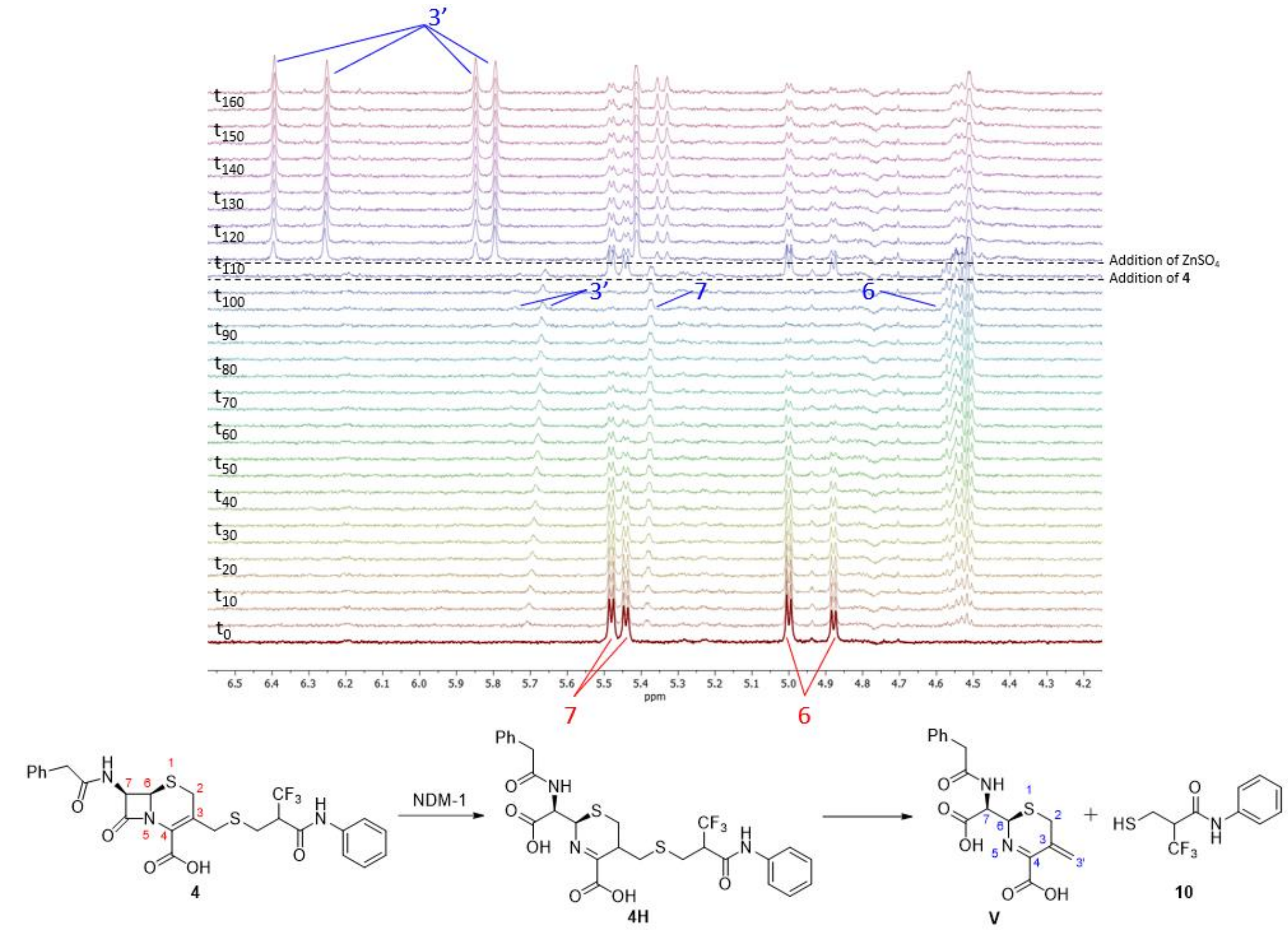

Figure S17. ^{1}H NMR analysis of NDM-1-mediated degradation of **4**. Addition of conjugate (lower dashed line) and zinc sulfate (higher dashed line) after full consumption of **4**.

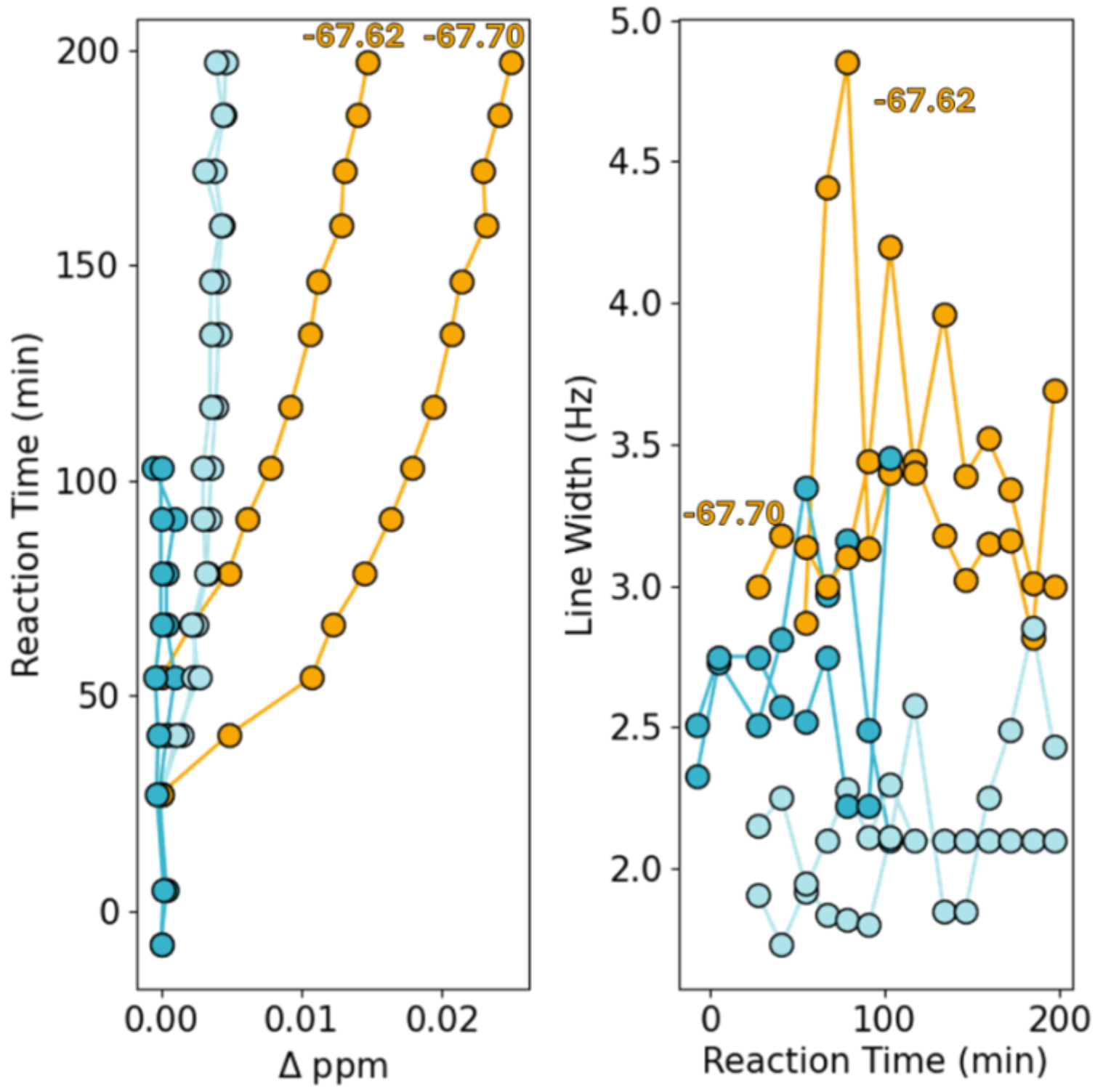

Figure S18. ^{19}F NMR analysis of NDM-1-mediated degradation of **4**. Left: chemical shift perturbations shown as a delta chemical shift. Right: changes in line width of the observed peaks over time.

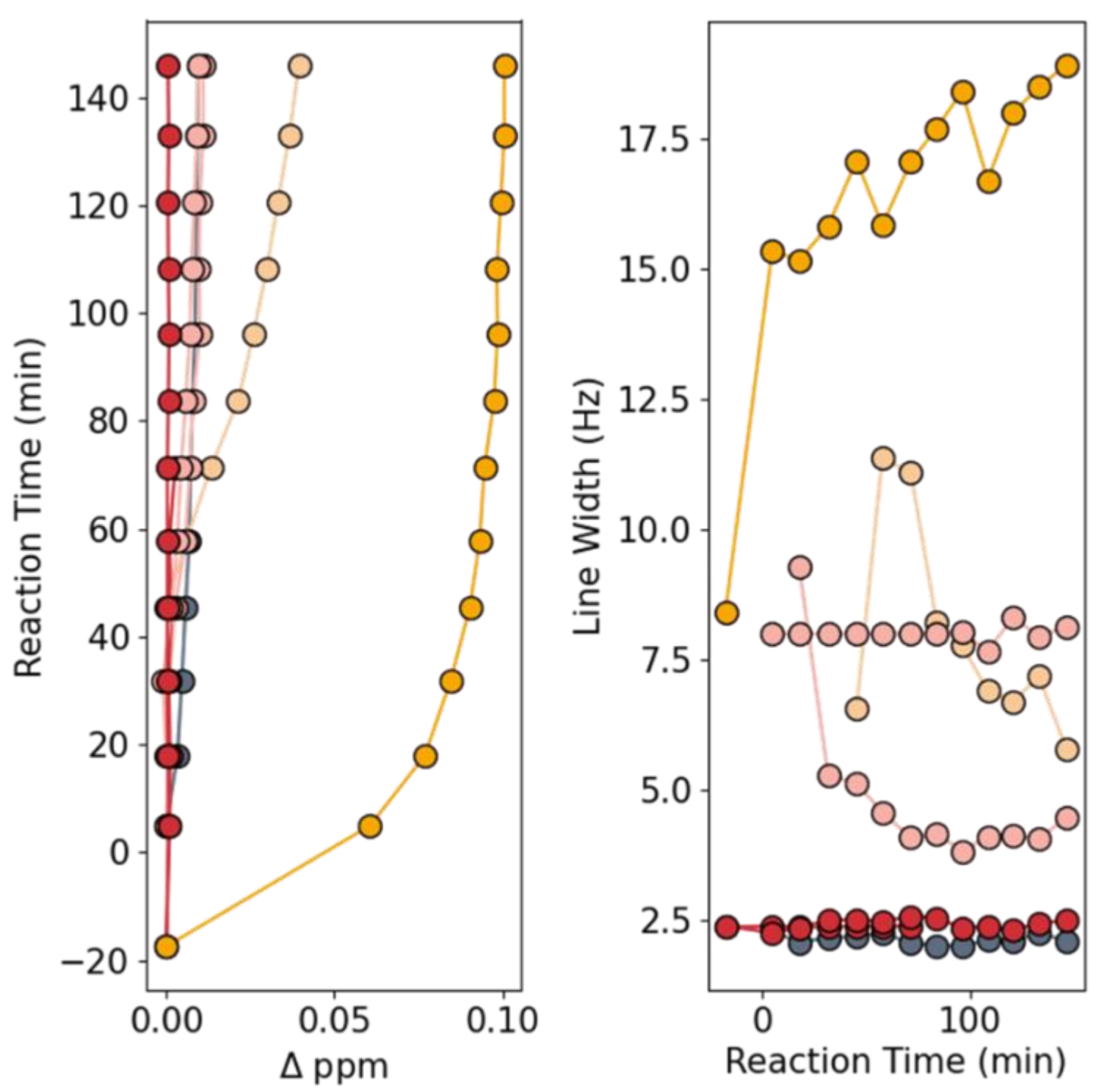

Figure S19. ^{19}F NMR analysis of NDM-1-mediated degradation of **5**. Left: chemical shift perturbations shown as a delta chemical shift. Right: changes in line width of the observed peaks over time.

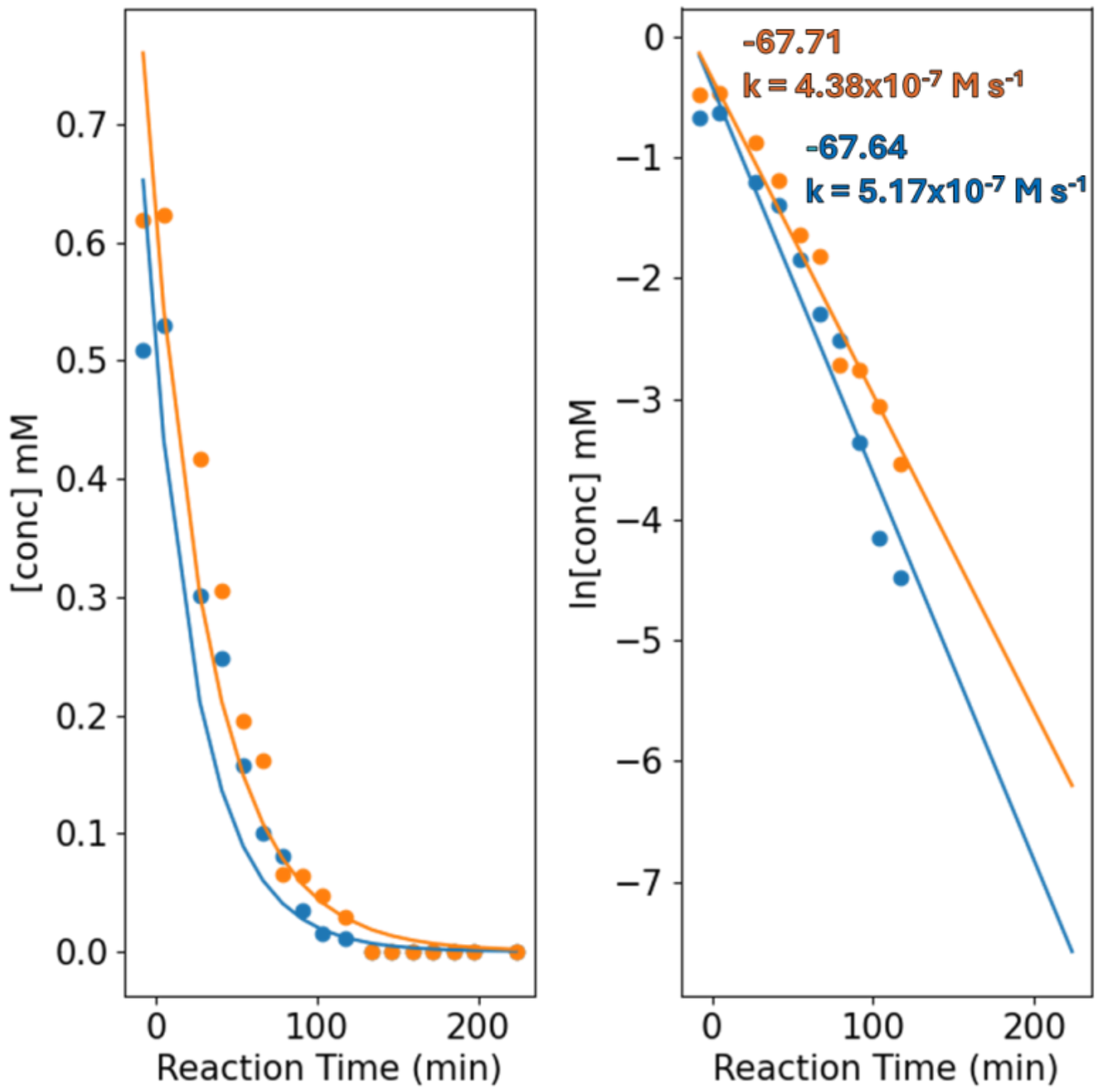

Figure S20. ^{19}F NMR curve fitting for NDM-1-mediated degradation of the diastereomers of **4**.

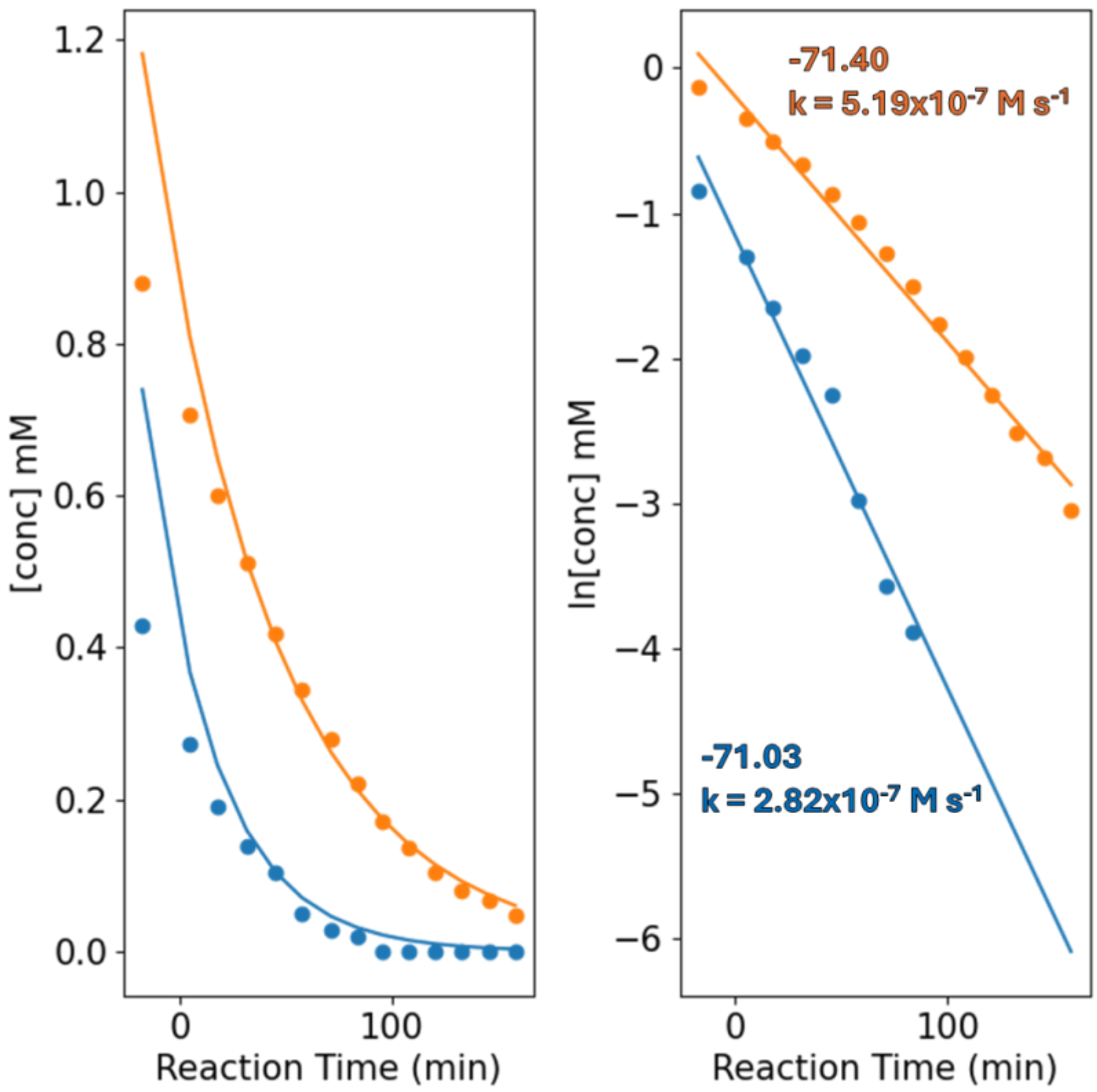

Figure S21. ^{19}F NMR curve fitting for NDM-1-mediated degradation of the diastereomers of **5**.

MBL inhibition assay and IC_{50} curves

All the compounds were tested for their inhibitory activity against the purified enzymes of NDM-1, VIM-2, IMP-2 and IMP-7. The molecules at 2-fold dilution (88-0.085938 µg/ml) were tested in triplicates in a direct competition assay with nitrocefin in HEPES buffer 50 mM pH 7.2, supplemented with 0.01% triton X-100 at room temperature. All inhibitors were preincubated with the respective metallo- β-lactamase for 10 minutes prior to substrate addition to allow enzyme–inhibitor equilibration. Unless otherwise stated, a 10 min preincubation was used for IC_{50} determinations. To assess the effect of preincubation time on inhibitory activity, IC_{50} values were additionally determined following 0, 10 and 30 min pre-incubation with the respective enzymes (Table S2). The reaction was initiated by adding 20 µM nitrocefin and the hydrolysis was recorded every 2 s for 2 minutes at A_{486}. IC_{50} values were calculated by plotting the *log[I] vs normalized response (variable slope)* program in GraphPad prism. The normalized response was obtained by taking the initial rate of hydrolysis at different concentrations of the inhibitor converted to the percentage of residual activity, compared to a reaction without inhibitor. The rate of the reaction without an inhibitor was considered as 100% activity.

$$\%\mathrm{RA} = \frac{100}{1 + 10^{(\log \mathrm{IC}_{50} - \log [\mathrm{I}]) * h}}$$

Where h is the Hill coefficient, IC_{50} is the half-maximal inhibitory concentration and %RA is the percentage of residual activity.

Table S1. IC_{50} values of conjugates and thiols against selected B1 MBLs.

		IC_{50} (μM)			
Compound	Corresponding thiol	NDM-1	VIM-2	IMP-2	IMP-7
**1**	8-TQ	1.92 ± 0.06<br>(2.31 ± 0.25[a])	1.99 ± 0.07<br>(3.64 ± 0.27[a])	1.4 ± 0.09	2.4 ± 0.17
**2**	L-captopril	31.81 ± 4.93	10.37 ± 1.64	22.44 ± 3.11	6.91 ± 0.49
**3**	**9**	27.76 ± 8.4	4.58 ± 0.22	19.24 ± 0.73	18.61 ± 1.32
**4**	**10**	3.76 ± 0.35	0.61 ± 0.05	11.28 ± 2.17	13.47 ± 1.75
**5**	**11**	8.22 ± 0.34	7.11 ± 0.37	6.07 ± 0.83	8.61 ± 0.45
**6**	**12**	4.5 ± 0.38	33.64 ± 4.49	2.79 ± 0.93	4.12 ± 0.11
**7**	**13**	91.72 ± 5.08	15.34 ± 1.22	82.33 ± 26.17	21.84 ± 0.84
**8**	**14**	2.02 ± 0.55	4.28 ± 0.86	1.98 ± 0.52	3.19 ± 0.11
**9**	-	4.25 ± 0.08<br>(20 ± 2[b])	7.39 ± 0.54	6.18 ± 0.47	33.82 ± 2.58
**10**	-	4.28 ± 0.50<br>(3.4 ± 0.5[b])	5.95 ± 0.96	5.82 ± 0.16	17 ± 0.45
**11**	-	27.65 ± 0.95<br>(22 ± 6[b])	86.14 ± 7.76	43.53 ± 3.59	89.51 ± 0.46
**12**	-	(5.6 ± 1.4[b])	-	-	-
**13**	-	(60 ± 20[b])	-	-	-
**14**	-	(0.3 ± 0.1[b])	-	-	-
**16**[c]	-	>300	49.94 ± 5.18	>300	60.05 ± 6.94
L-captopril	-	13.12 ± 3.23<br>(7 ± 2[b])	4.487 ± 0.19	6.16 ± 0.31	10.15 ± 0.76

[a-b] Previously reported IC_{50} values in a[2] and b[1]. [c] Structure **16** (right), synthesized as reported.[4]

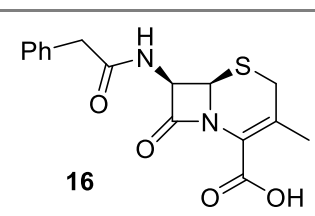

16

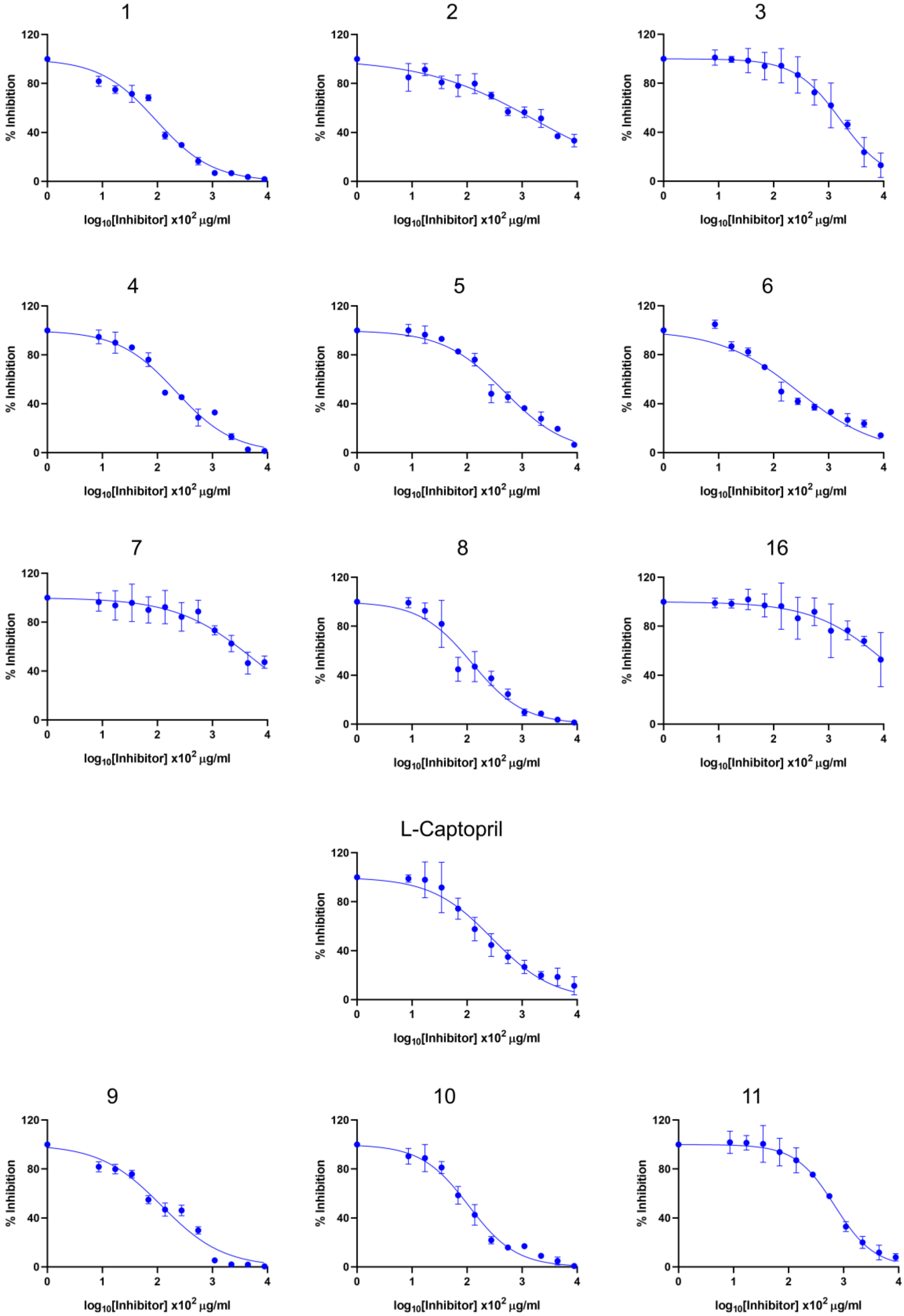

Figure S22. IC_{50} curves for compounds **1-11**, **16** and L-captopril against NDM-1.

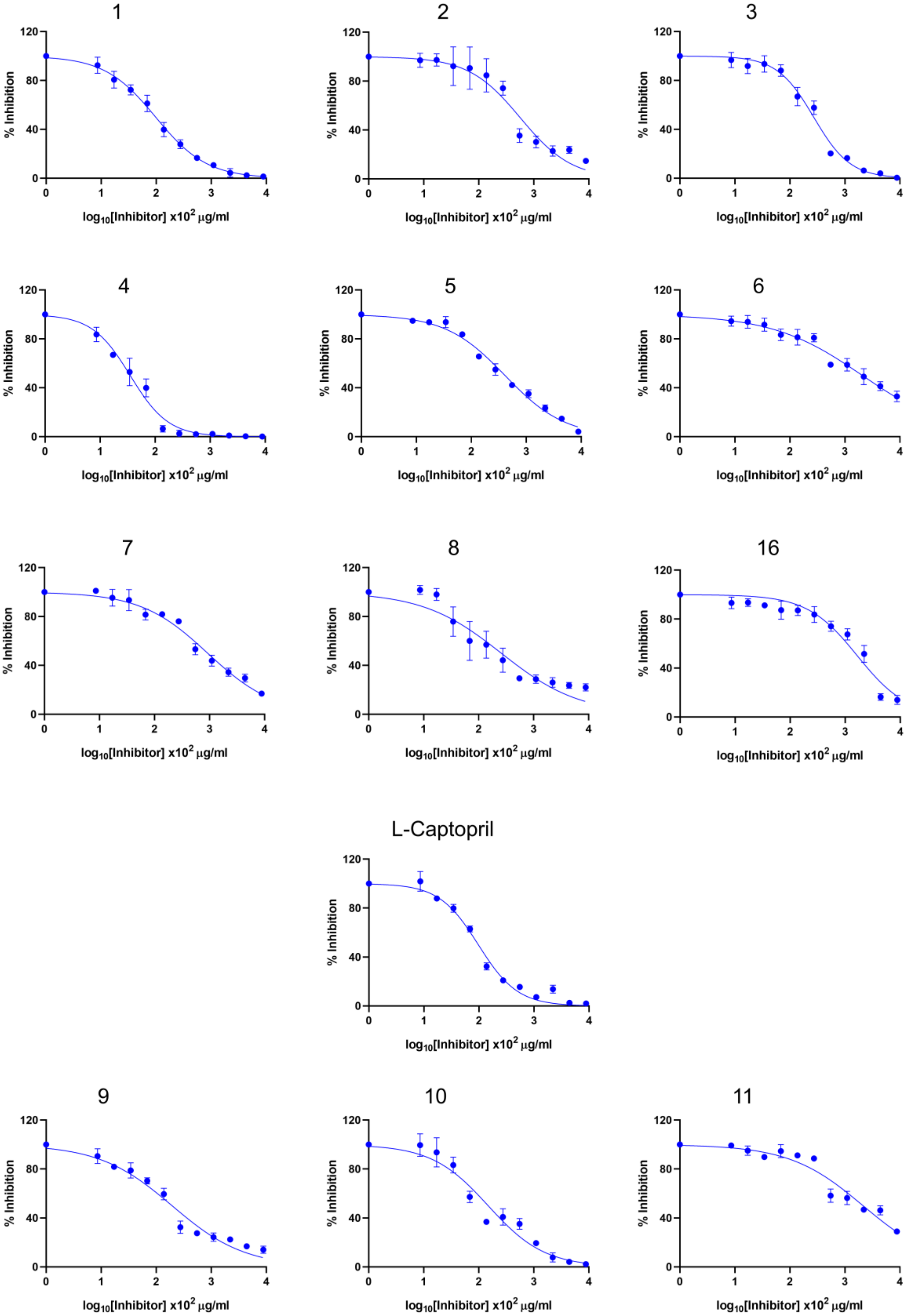

Figure S23. IC_{50} curves for compounds **1-11**, **16** and L-captopril against VIM-2.

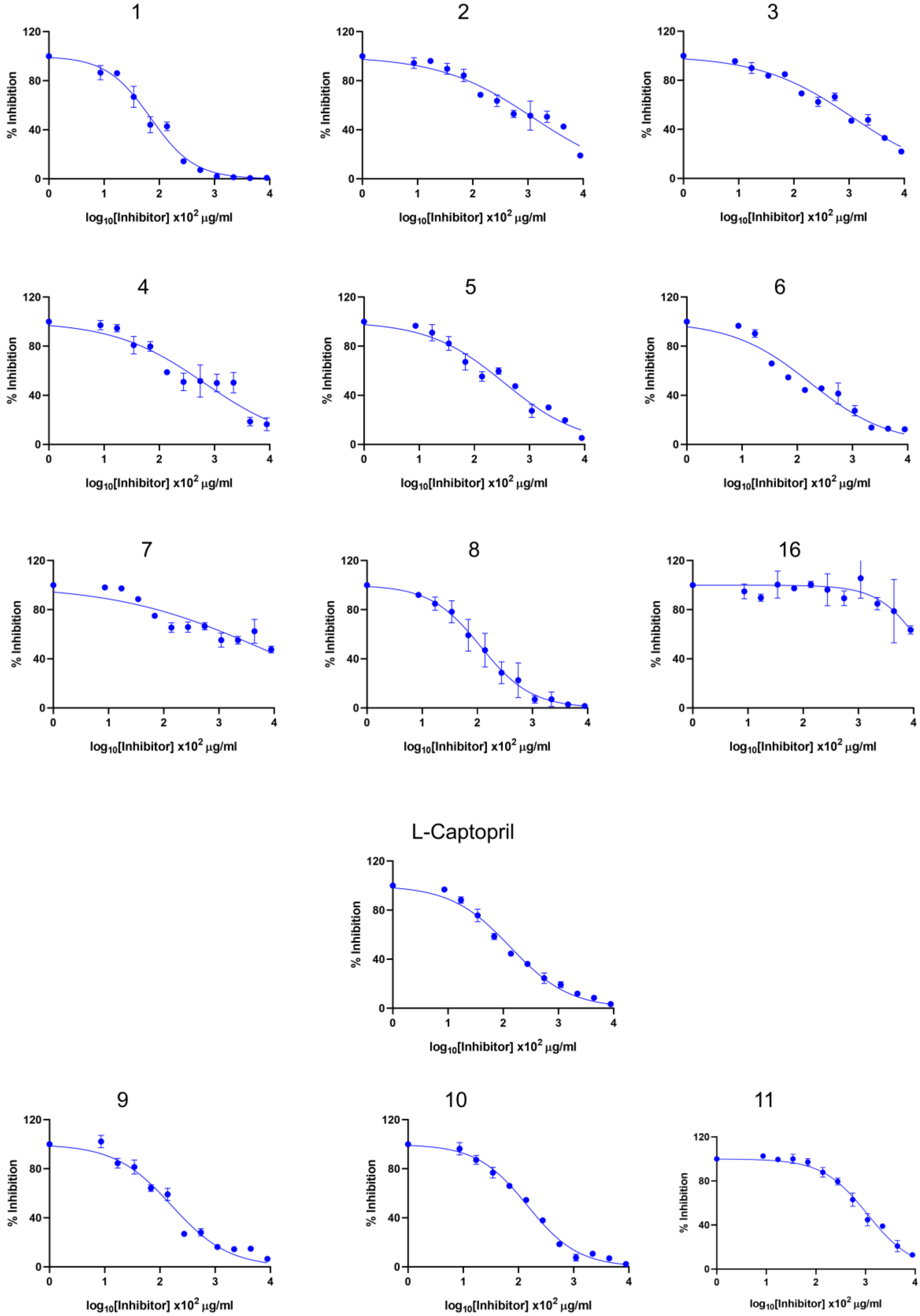

Figure S24. IC_{50} curves for compounds **1-11**, **16** and L-captopril against IMP-2.

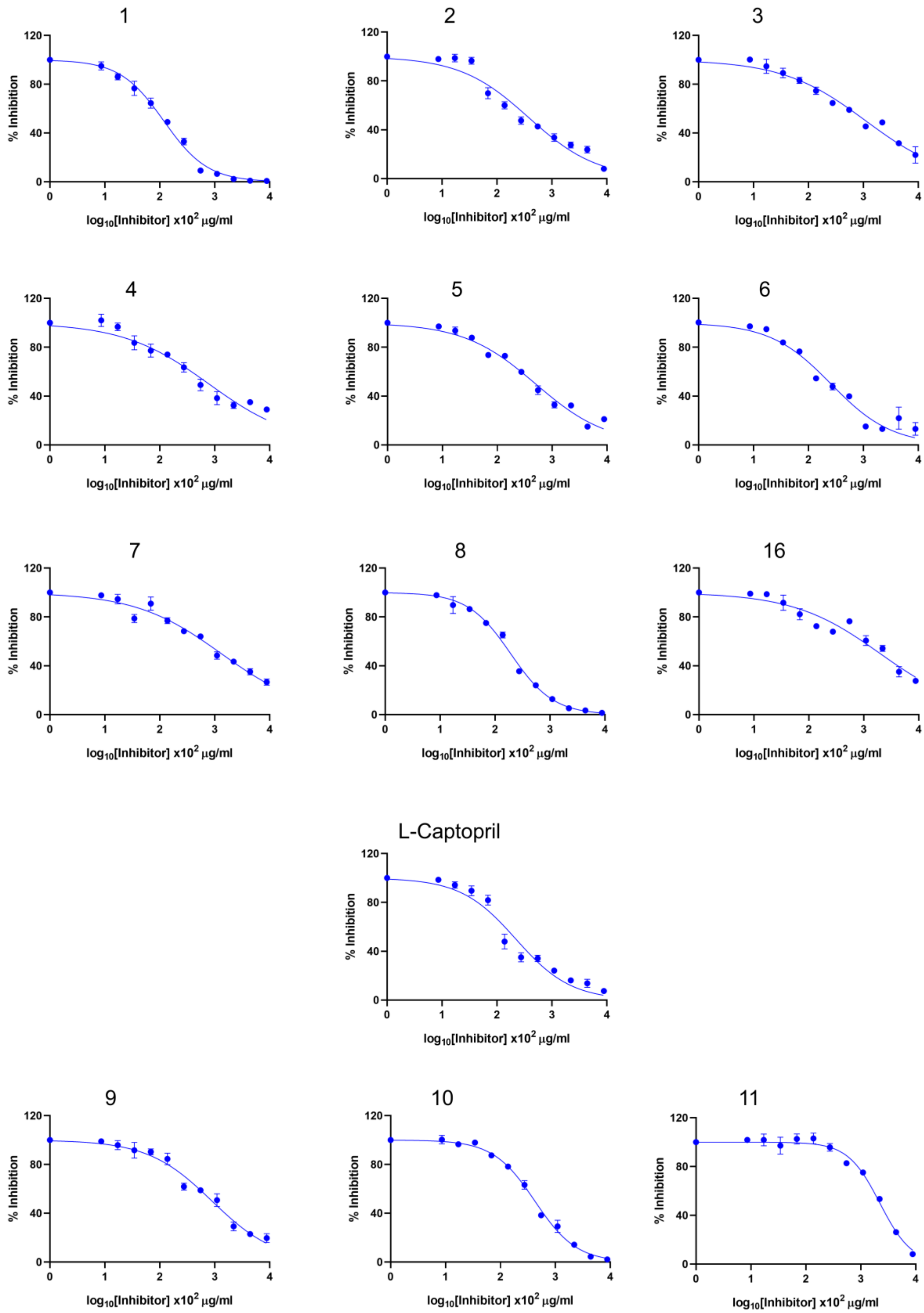

Figure S25. IC_{50} curves for compounds **1-11**, **16** and L-captopril against IMP-7.

Table S2: IC_{50} values of conjugates against selected B1 MBLs at varying pre-incubation times.

Comp ID	Direct	10 min incubation	30 min incubation
		**IC_{50} (μM) against NDM-1**	
**2**	90.44 ± 7.28	37.29 ± 1.15	31.06 ± 1.20
**3**	32.09 ± 1.47	2.75 ± 0.34	2.48 ± 0.10
**4**	5.61 ± 0.38	3.64 ± 0.61	0.63 ± 0.06
**5**	25.50 ± 2.88	5.79 ± 0.26	16.03 ± 0.80
**7**	550.91 ± 100.20	56.20 ± 6.73	72.72 ± 7.43
**8**	66.09 ± 3.71	2.26 ± 0.30	1.49 ± 0.07
		**IC_{50} (μM) against VIM-2**	
**2**	10.07 ± 1.81	7.56 ± 0.30	7.50 ± 0.14
**3**	15.17 ± 1.02	2.97 ± 0.26	0.56 ± 0.04
**4**	178.69 ± 14.91	1.82 ± 0.09	0.51 ± 0.03
**5**	10.52 ± 0.26	5.30 ± 0.09	4.86 ± 0.32
**7**	185.88 ± 6.37	13.05 ± 1.18	34.44 ± 0.82
**8**	13.44 ± 0.91	3.46 ± 0.23	0.92 ± 0.08
		**IC_{50} (μM) against IMP-2**	
**2**	61.23 ± 3.72	20.18 ± 0.39	17.86 ± 1.24
**3**	31.37 ± 1.64	17.30 ± 0.17	11.95 ± 0.14
**4**	24.24 ± 1.66	8.63 ± 0.99	5.31 ± 0.13
**5**	12.85 ± 1.15	5.74 ± 0.20	0.61 ± 0.02
**7**	148.10 ± 15.13	66.46 ± 4.03	11.27 ± 0.69
**8**	280.81 ± 71.48	2.13 ± 0.15	0.67 ± 0.05
		**IC_{50} (μM) against IMP-7**	
**2**	71.12 ± 8.11	7.20 ± 0.28	3.62 ± 0.19
**3**	36.81 ± 3.98	13.88 ± 0.78	17.84 ± 2.01
**4**	18.20 ± 2.42	13.05 ± 0.75	4.91 ± 0.40
**5**	23.10 ± 0.62	6.48 ± 0.60	4.41 ± 0.66
**7**	112.02 ± 3.10	19.03 ± 1.03	12.01 ± 0.74
**8**	202.21 ± 34.12	2.50 ± 0.17	0.72 ± 0.05

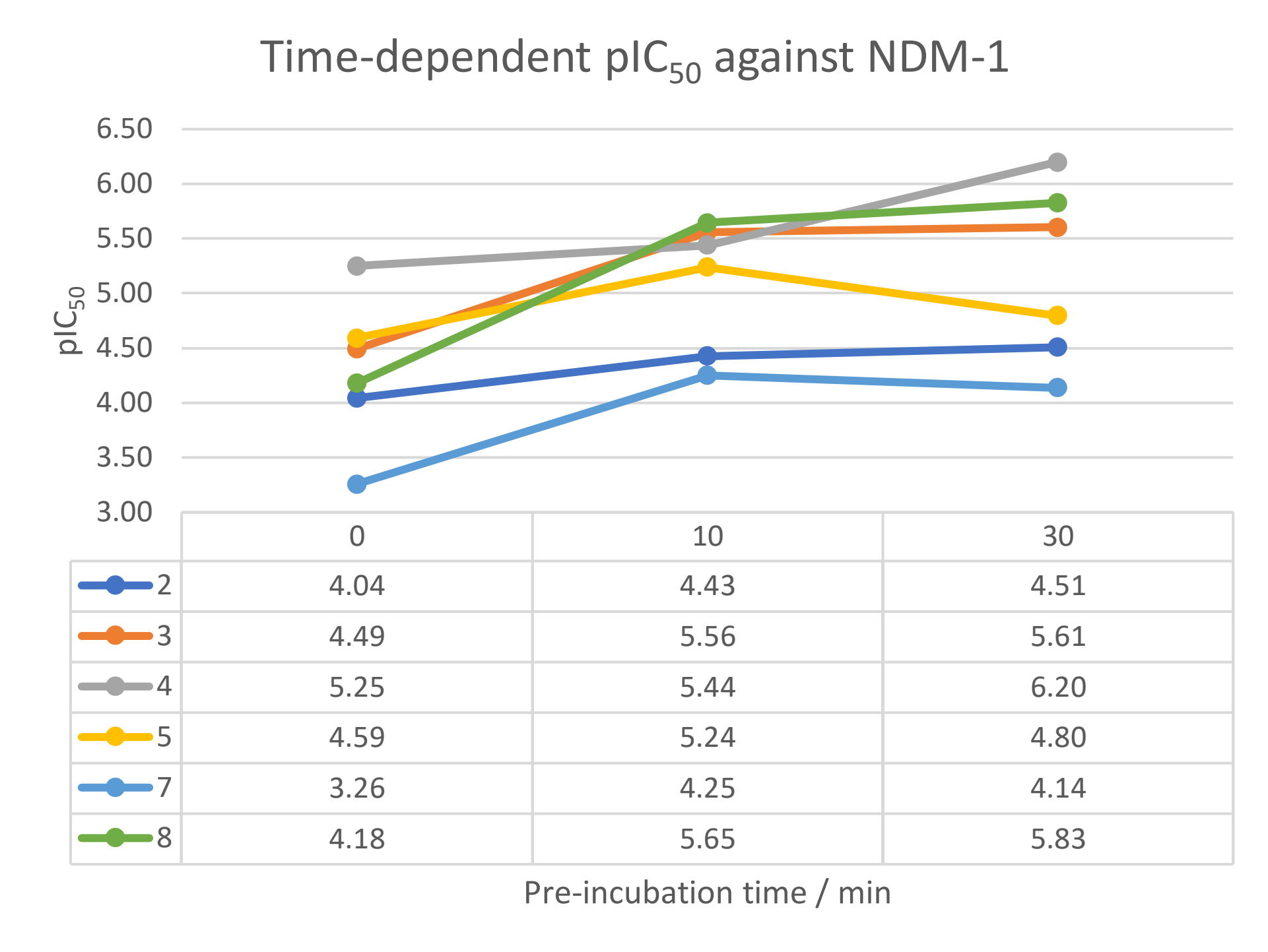

	0	10	30
2	4.04	4.43	4.51
3	4.49	5.56	5.61
4	5.25	5.44	6.20
5	4.59	5.24	4.80
7	3.26	4.25	4.14
8	4.18	5.65	5.83

Figure S26. Influence of pre-incubation time on pIC$_{50}$ against NDM-1.

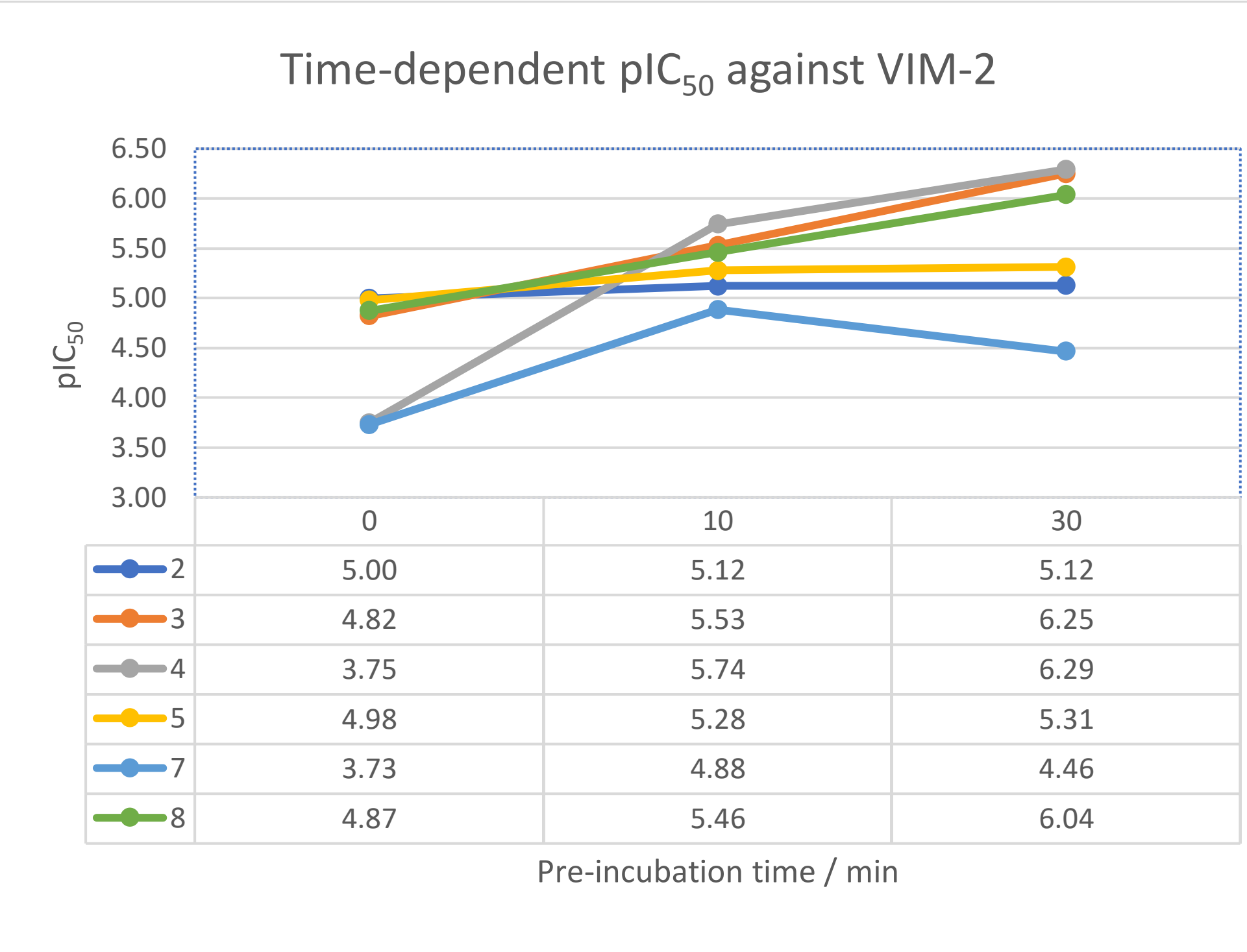

	0	10	30
2	5.00	5.12	5.12
3	4.82	5.53	6.25
4	3.75	5.74	6.29
5	4.98	5.28	5.31
7	3.73	4.88	4.46
8	4.87	5.46	6.04

Figure S27. Influence of pre-incubation time on pIC$_{50}$ against VIM-2.

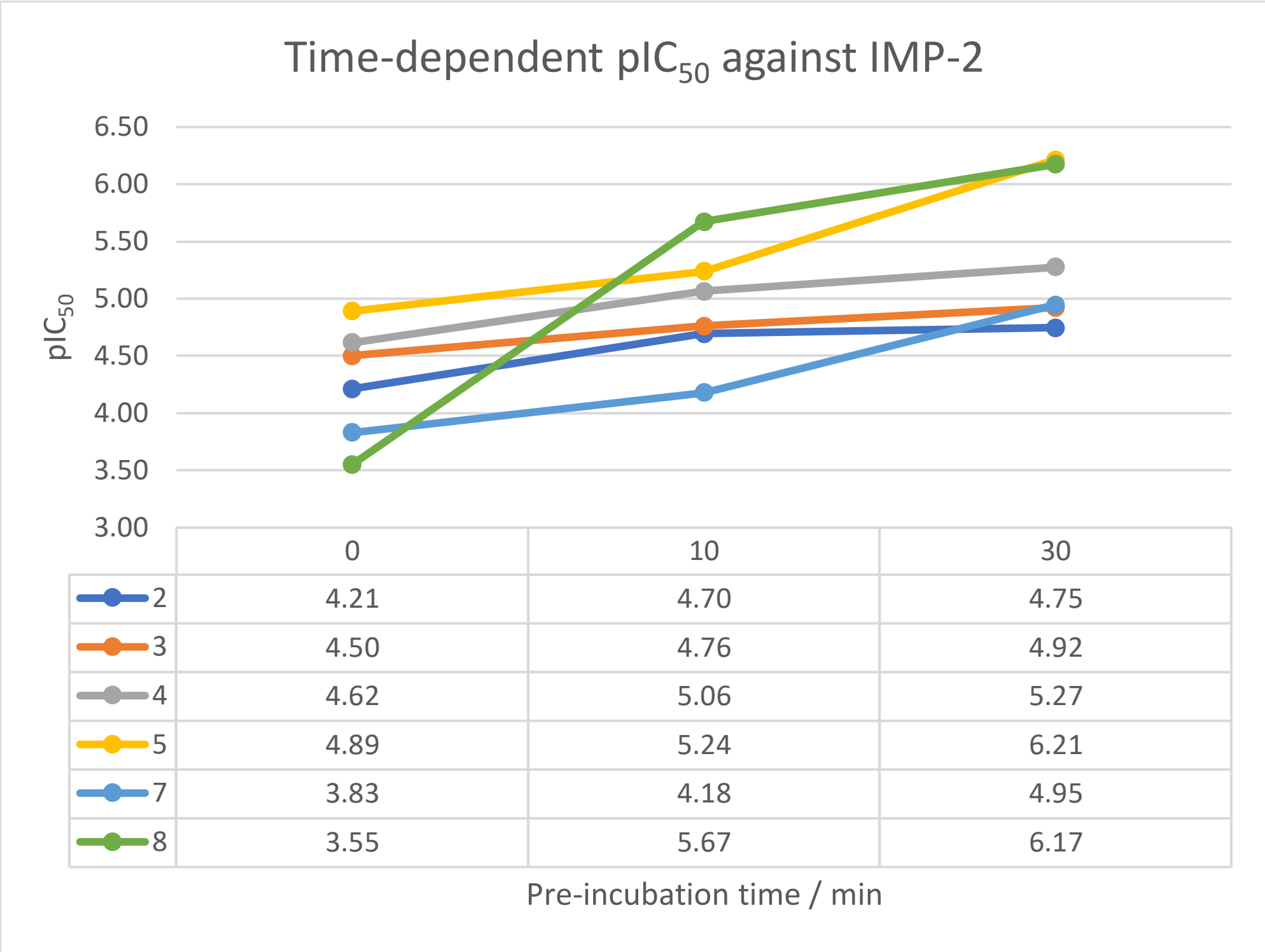

	0	10	30
2	4.21	4.70	4.75
3	4.50	4.76	4.92
4	4.62	5.06	5.27
5	4.89	5.24	6.21
7	3.83	4.18	4.95
8	3.55	5.67	6.17

Figure S28. Influence of pre-incubation time on pIC_{50} against IMP-2.

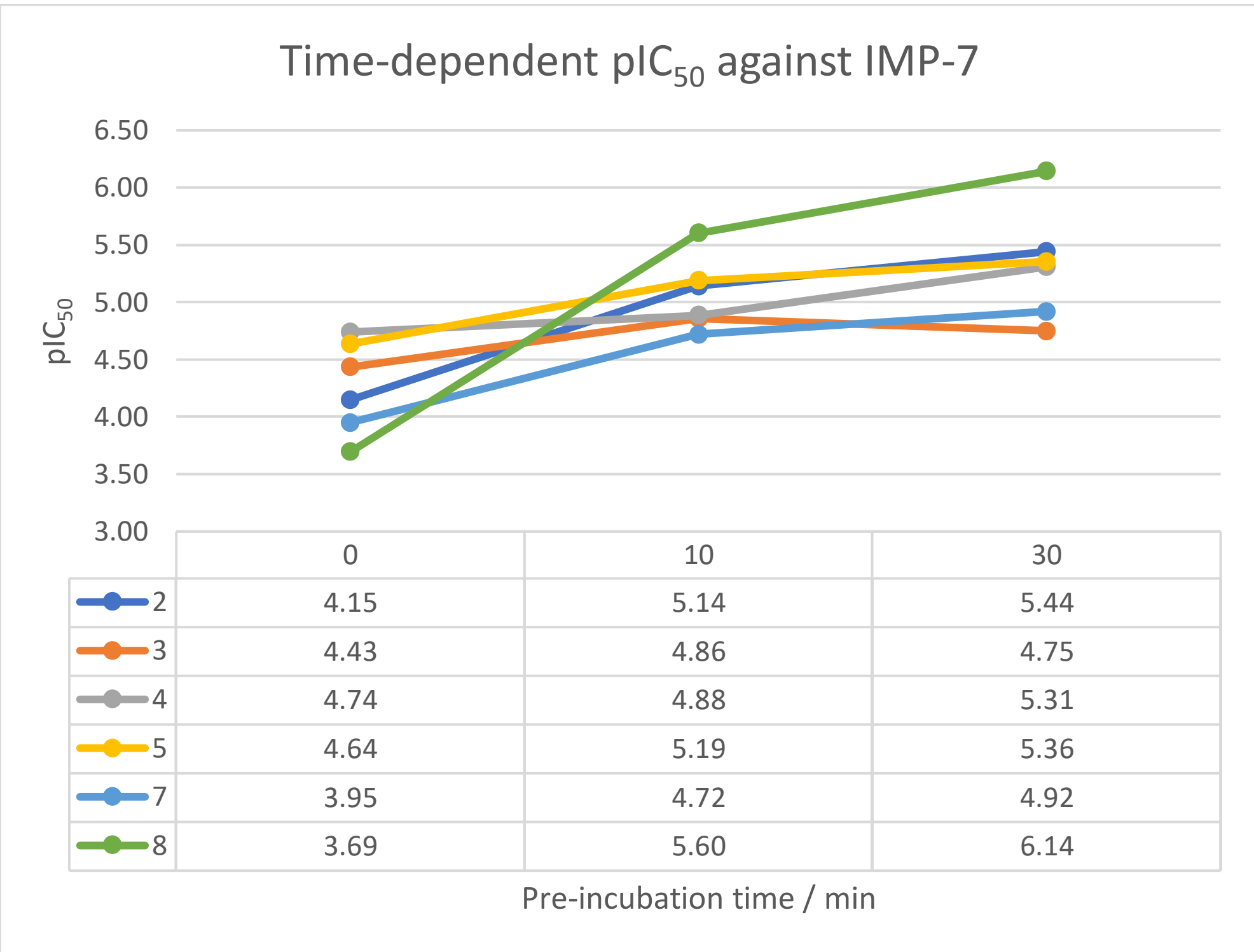

	0	10	30
2	4.15	5.14	5.44
3	4.43	4.86	4.75
4	4.74	4.88	5.31
5	4.64	5.19	5.36
7	3.95	4.72	4.92
8	3.69	5.60	6.14

Figure S29. Influence of pre-incubation time on pIC_{50} against IMP-7.

Meropenem restoration assay in bacteria

The determination of the reversal potential of molecules in combination with meropenem (MEM) involved conducting a series of two-fold serial dilutions of meropenem while maintaining a fixed concentration of each molecule (256 µg/ml). NDM-1 producing *E. coli* bacterial cultures were grown overnight at 37°C and 180 rpm in Mueller Hinton (MH) broth. Subsequently, these cultures were subcultured by adding 1% overnight culture to fresh MH broth and allowed to grow until reaching an optical density at 600 nm (OD600) of 0.3. To conduct the reversal assay, a two-fold dilution of meropenem was prepared in the final 200 µL of MH broth, with each row supplemented with a fixed concentration of the respective molecule (as depicted in the following figure). Subsequently, 10^5 CFU/mL of bacteria were added to initiate the assay. The plates were subjected to steady-state incubation at 37°C for 16 hours, and bacterial growth was measured at OD600.

Table S3: Potentiation of meropenem activity by conjugates against NDM-1-producing *E. coli*.

Compound	MIC alone (µg/mL)	Meropenem MIC alone (µg/mL)	Meropenem MIC in combination with compound (256 µg/mL) (µg/mL)	Fold reduction
**L-Captopril**	>256	32	< 1	>32×
**1**	>256	32	< 1	>32×
**2**	>256	32	32	1×
**3**	>256	32	16	2×
**4**	>256	32	16	2×
**5**	>256	32	16	2×
**6**	>256	32	16	2×
**7**	>256	32	32	1×
**8**	>256	32	4	8×
**16**	>256	32	32	1×

1H and 13C NMR spectra of final compounds

(6R,7R)-3-((((S)-3-((S)-2-carboxypyrrolidin-1-yl)-2-methyl-3-oxopropyl)thio)methyl)-8-oxo-7-(2-phenylacetamido)-5-thia-1-azabicyclo[4.2.0]oct-2-ene-2-carboxylic acid **(2)**

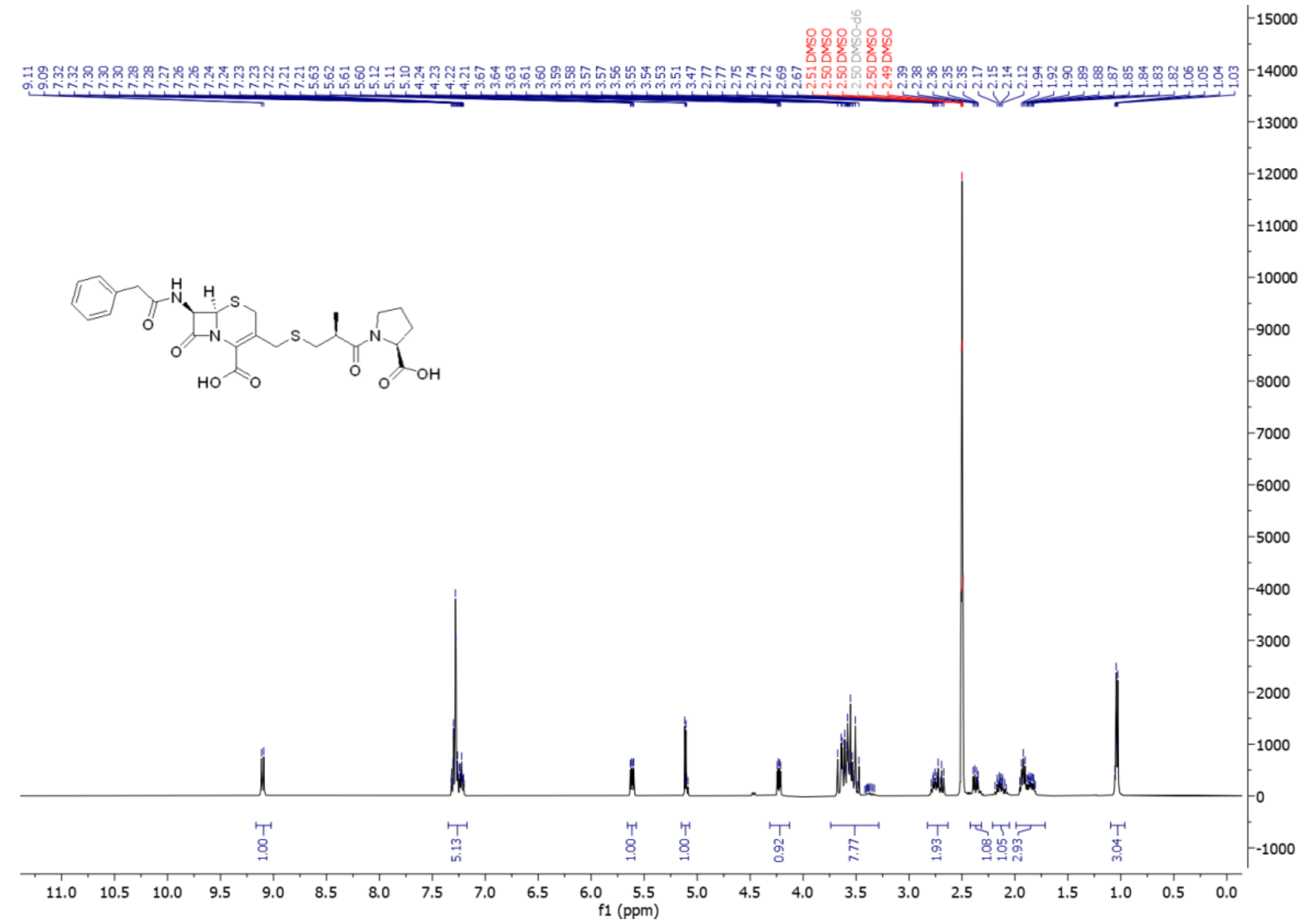

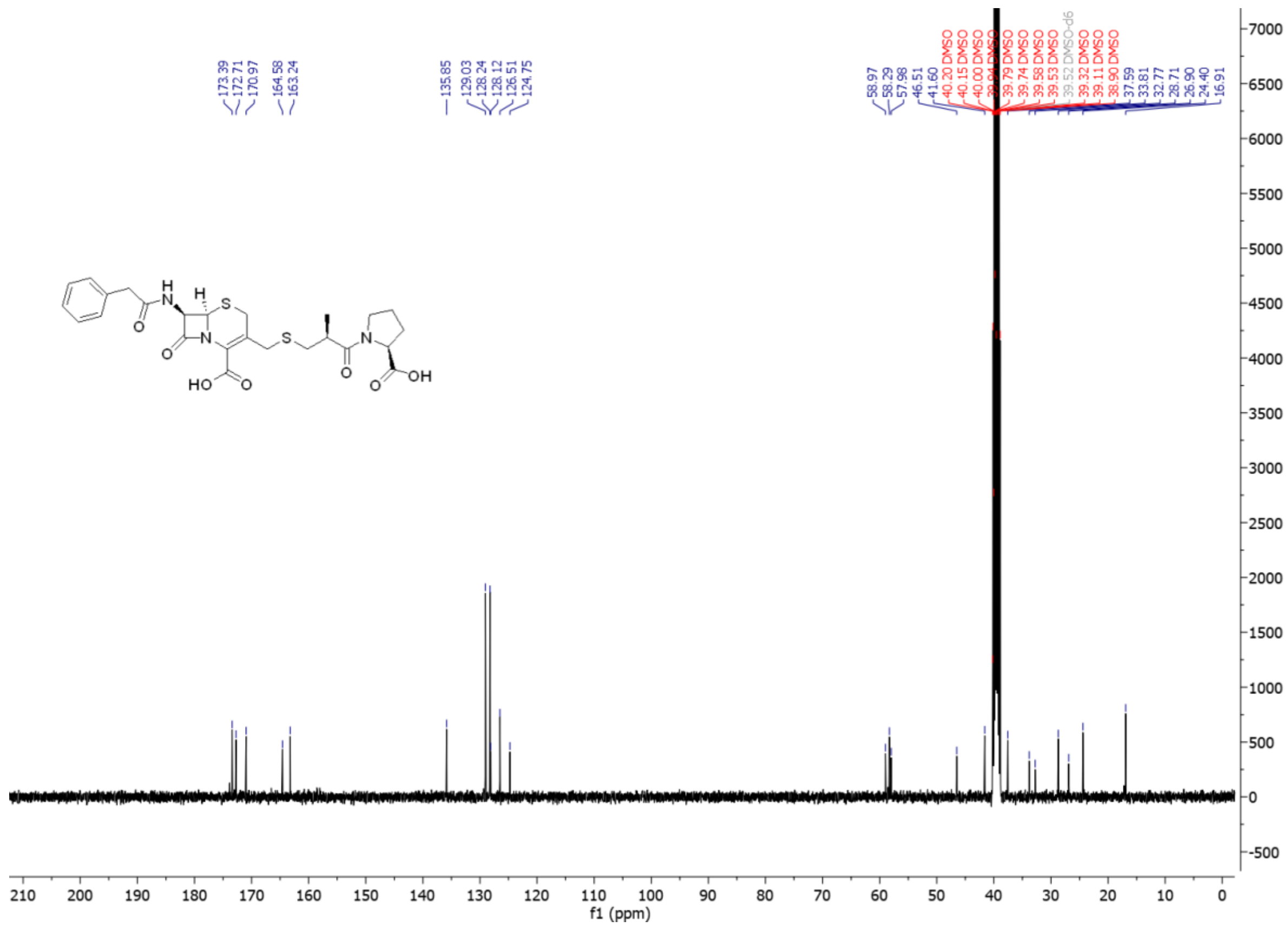

(6R,7R)-3-(((2-(benzylcarbamoyl)-3,3-trifluoropropyl)thio)methyl)-8-oxo-7-(2-phenylacetamido)-5-thia-1-azabicyclo[4.2.0]oct-2-ene-2-carboxylic acid **(3)**

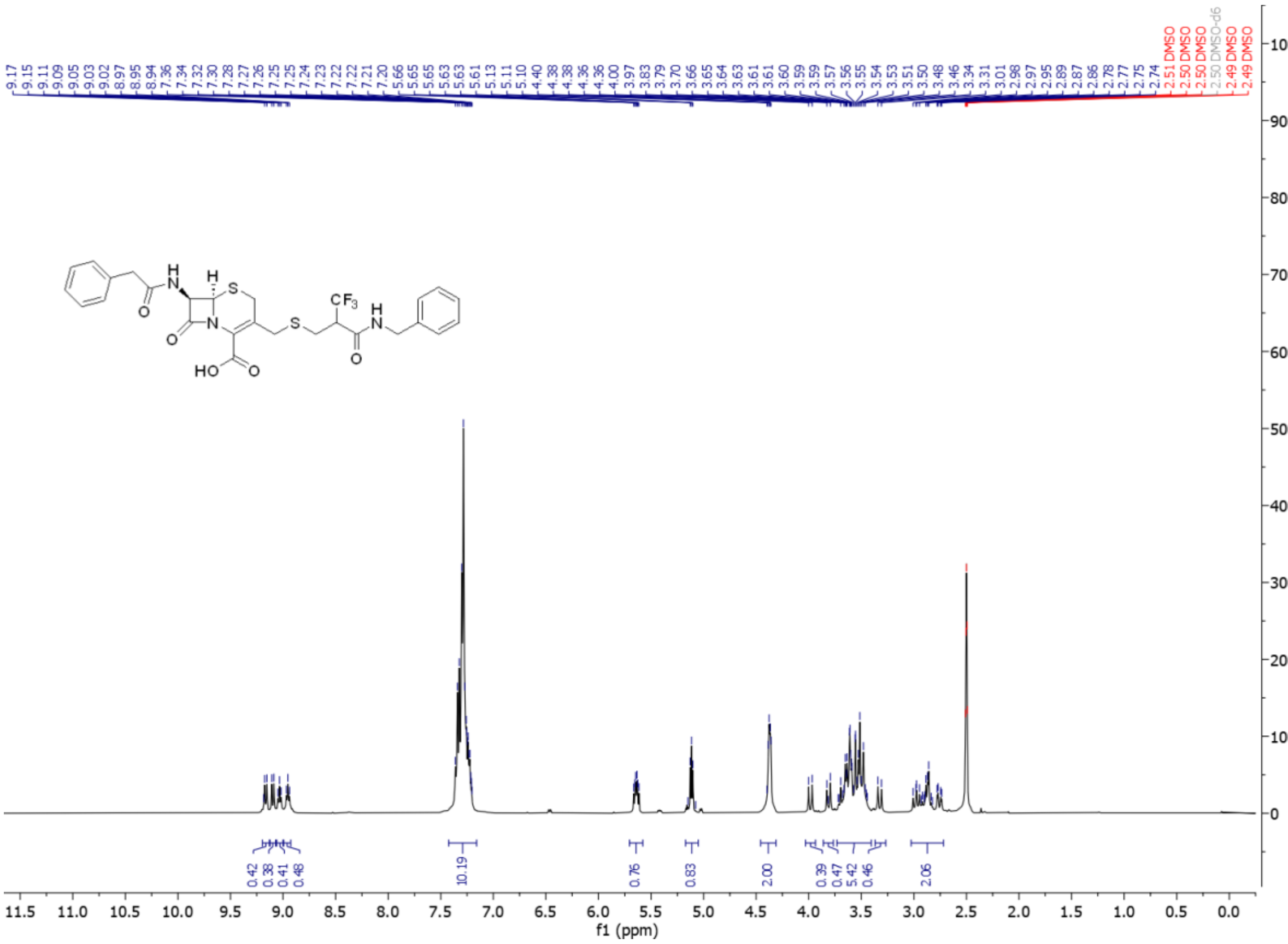

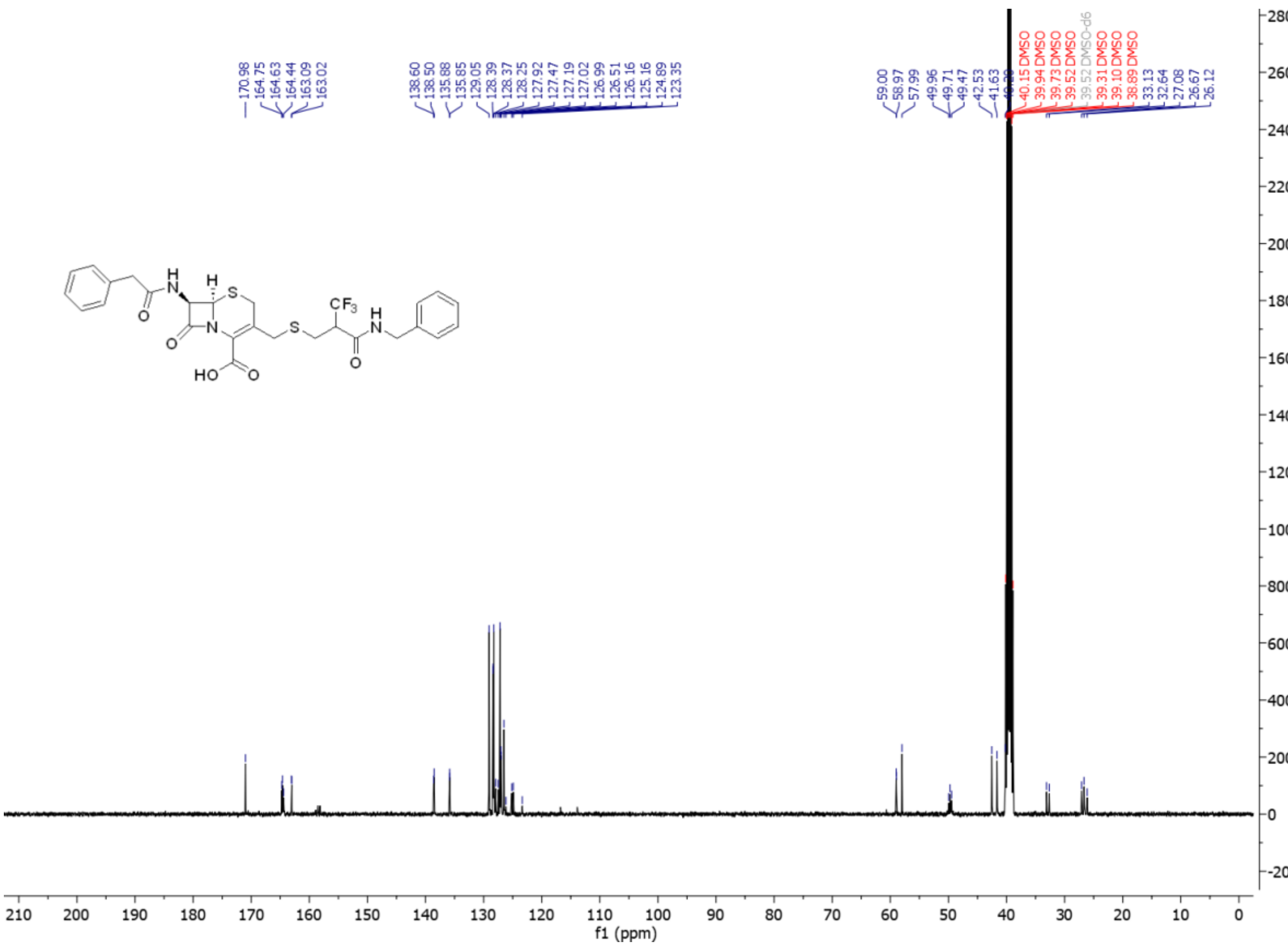

(6R,7R)-8-oxo-7-(2-phenylacetamido)-3-(((3,3,3-trifluoro-2-(phenylcarbamoyl)propyl)thio)methyl)-5-thia-1-azabicyclo[4.2.0]oct-2-ene-2-carboxylic acid **(4)**

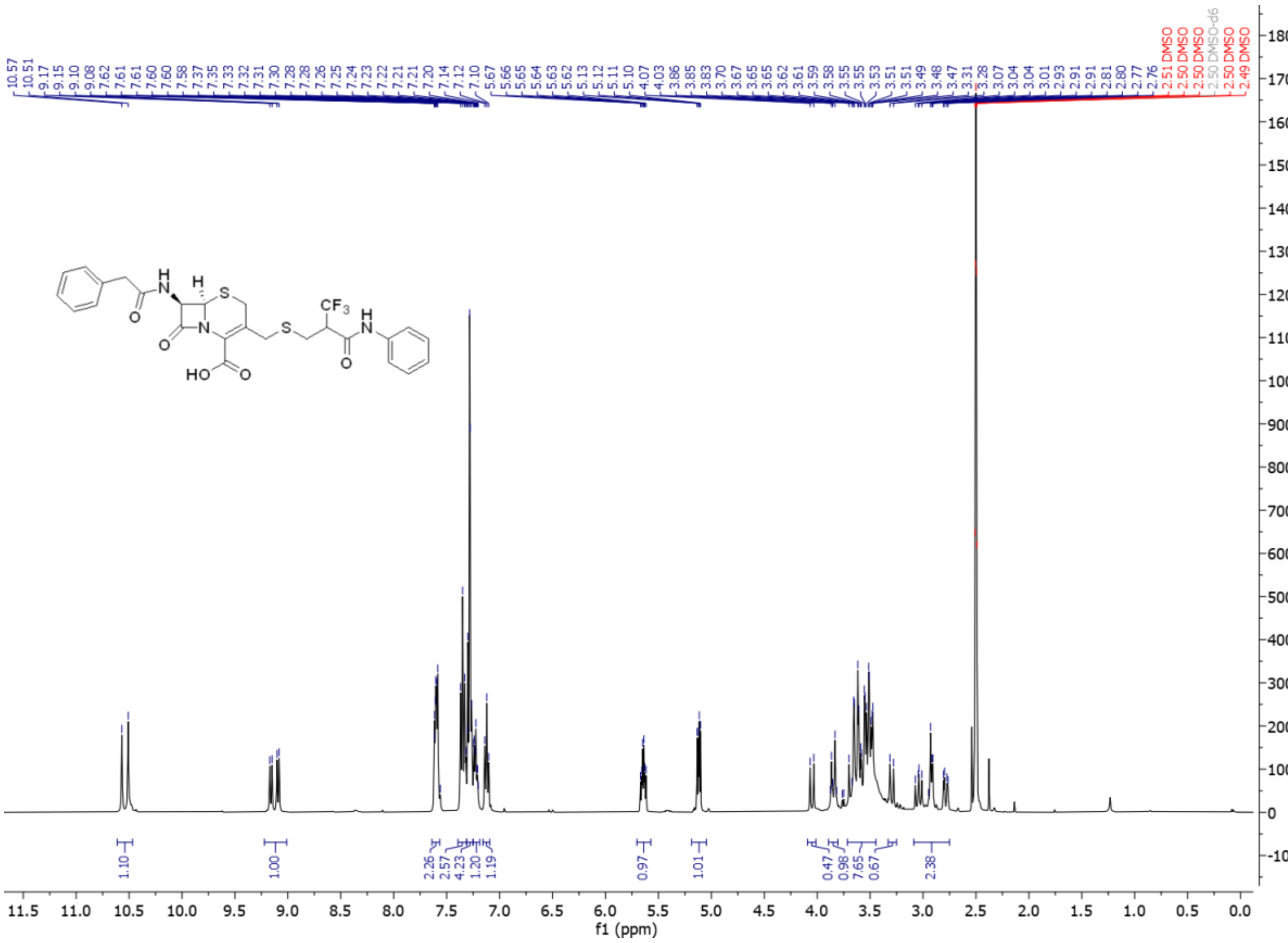

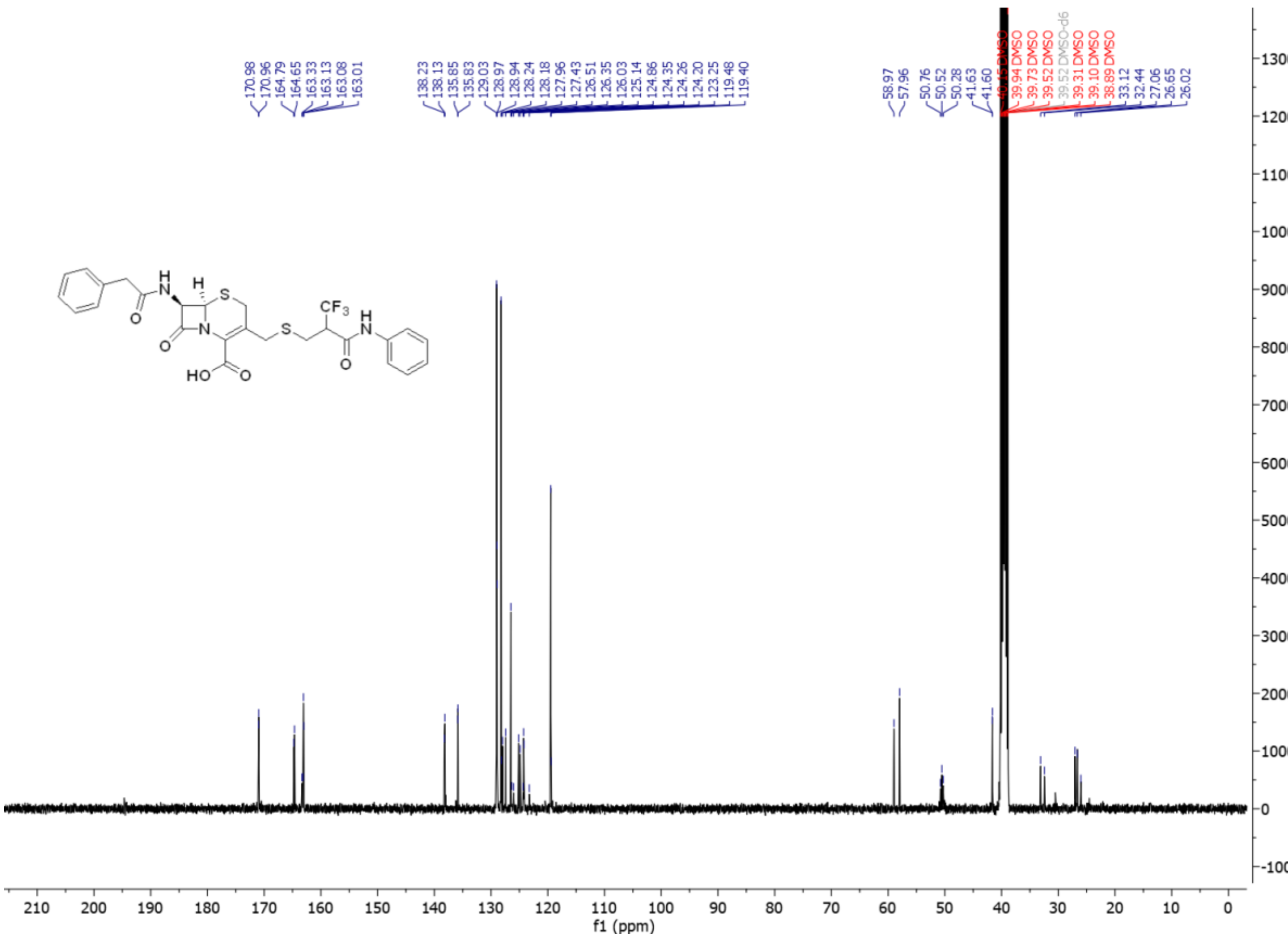

(6R,7R)-8-oxo-7-(2-phenylacetamido)-3-(((1,1,1-trifluoro-4-oxo-4-(phenylamino)butan-2-yl)thio)methyl)-5-thia-1-azabicyclo[4.2.0]oct-2-ene-2-carboxylic acid **(5)**

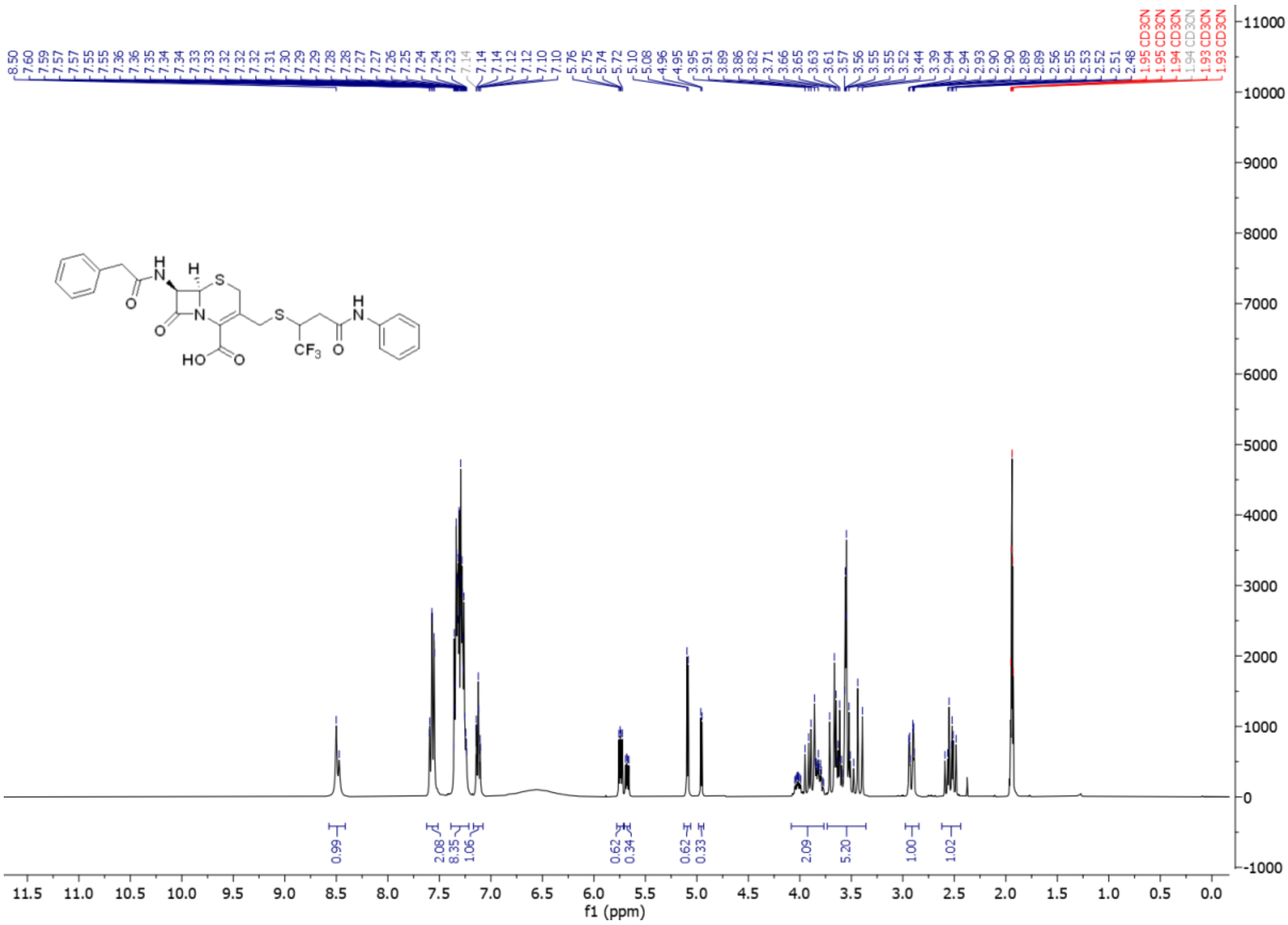

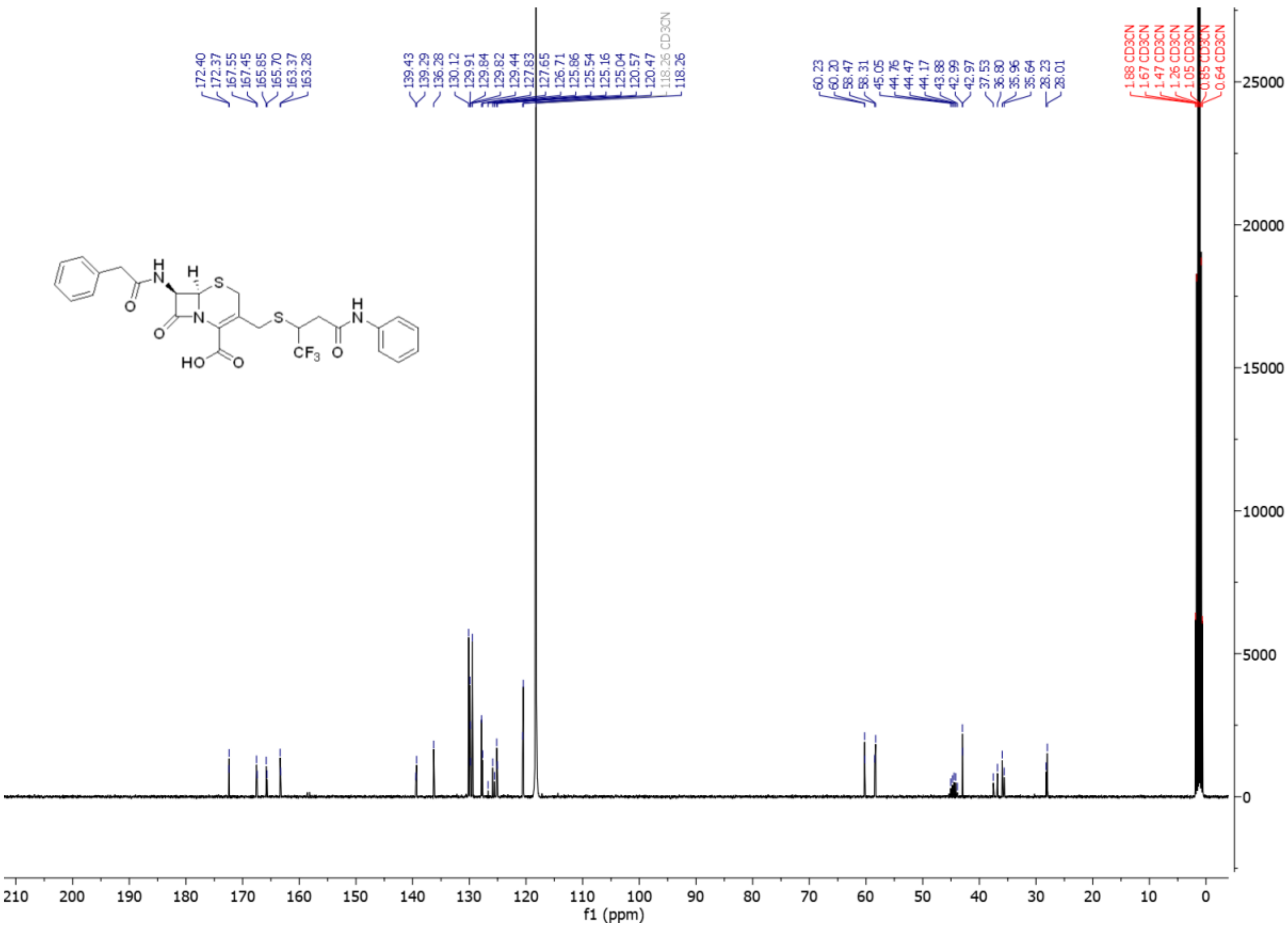

(6R,7R)-3-(((4-((4-carboxyphenyl)amino)-1,1,1-trifluoro-4-oxobutan-2-yl)thio)methyl)-8-oxo-7-(2-phenylacetamido)-5-thia-1-azabicyclo[4.2.0]oct-2-ene-2-carboxylic acid **(6)**

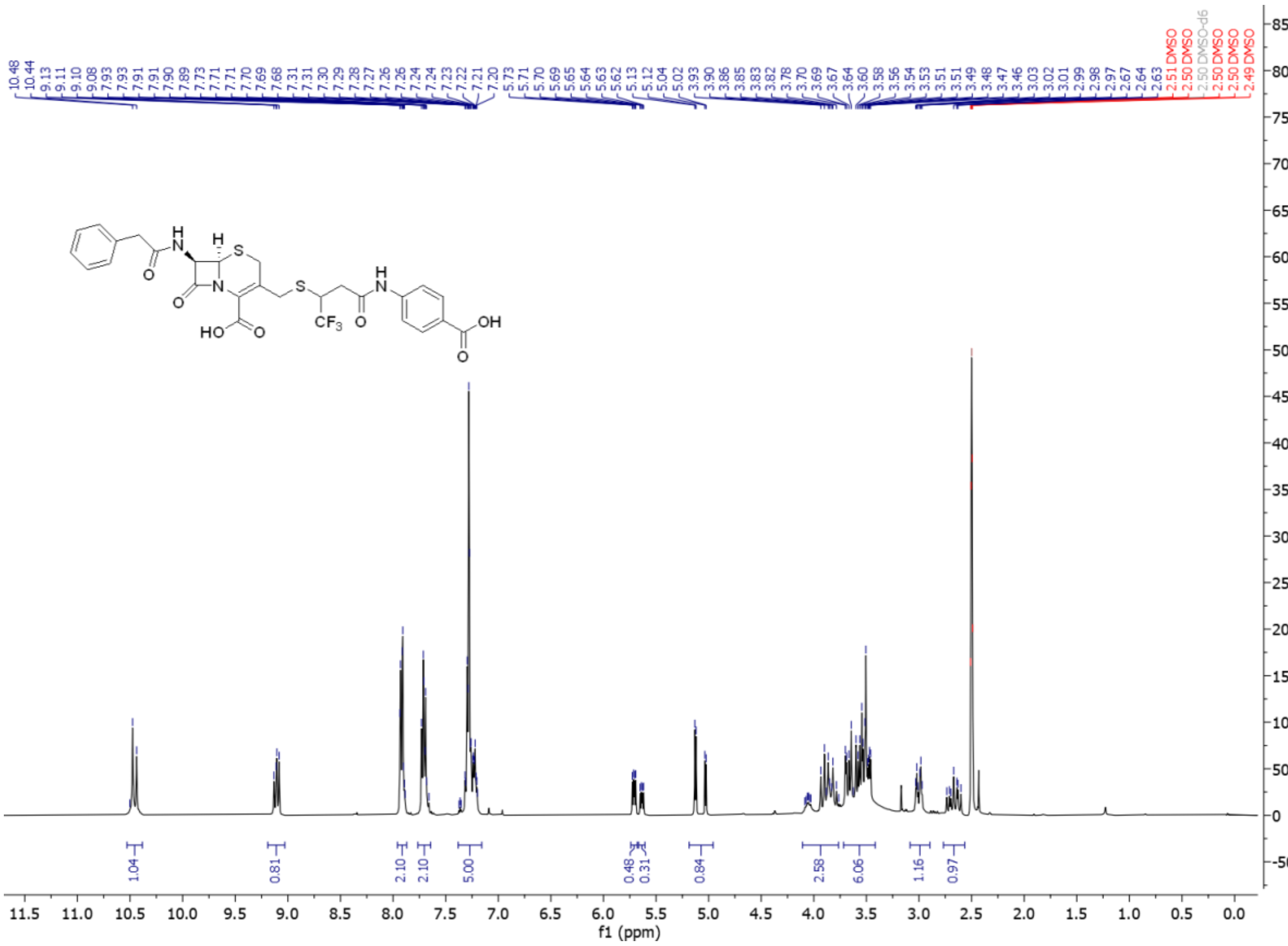

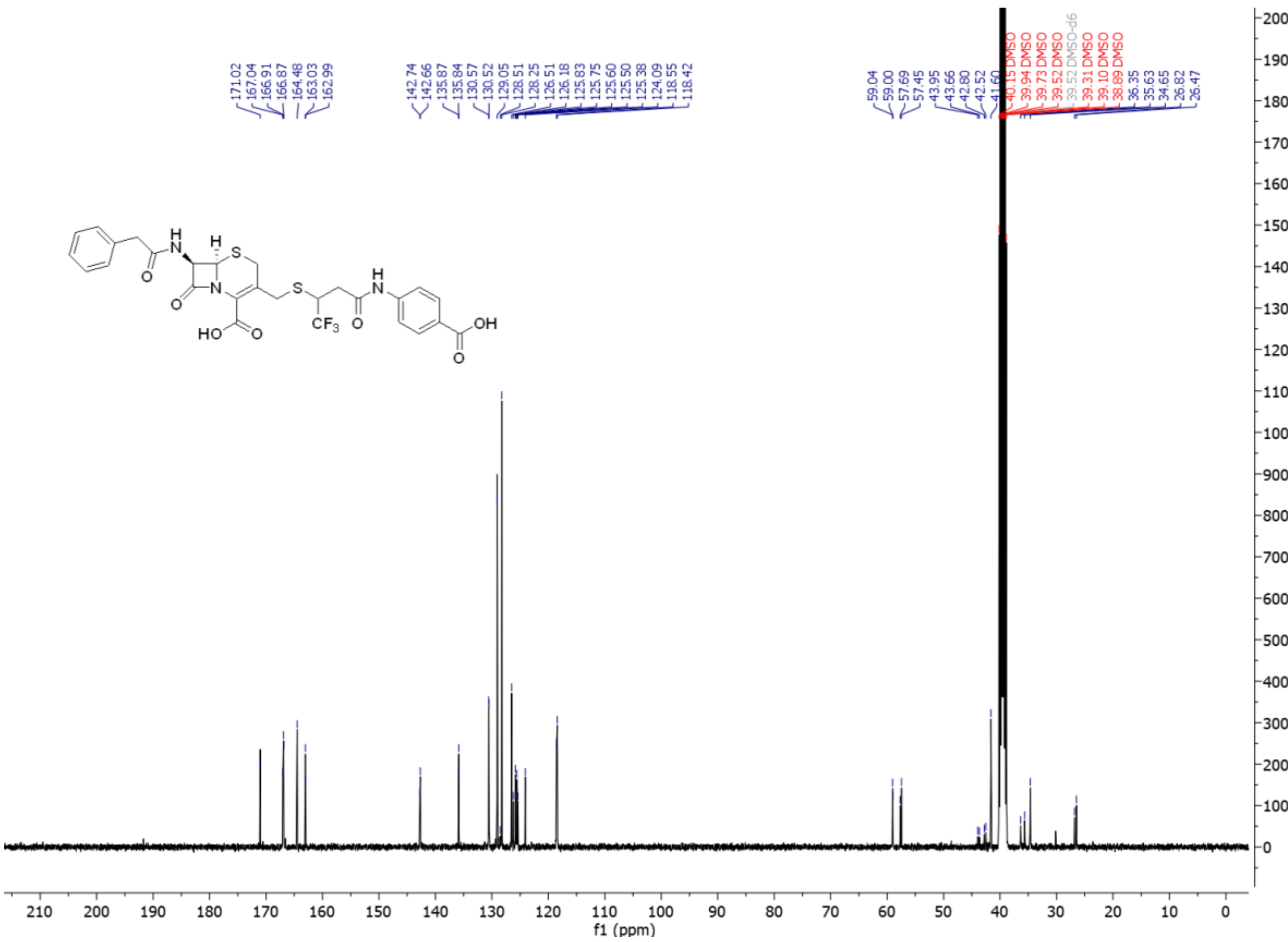

(6R,7R)-3-((((S)-2-((S)-2-carboxypyrrolidine-1-carbonyl)-3,3,3-trifluoropropyl)thio)methyl)-8-oxo-7-(2-phenylacetamido)-5-thia-1-azabicyclo[4.2.0]oct-2-ene-2-carboxylic acid **(7)**

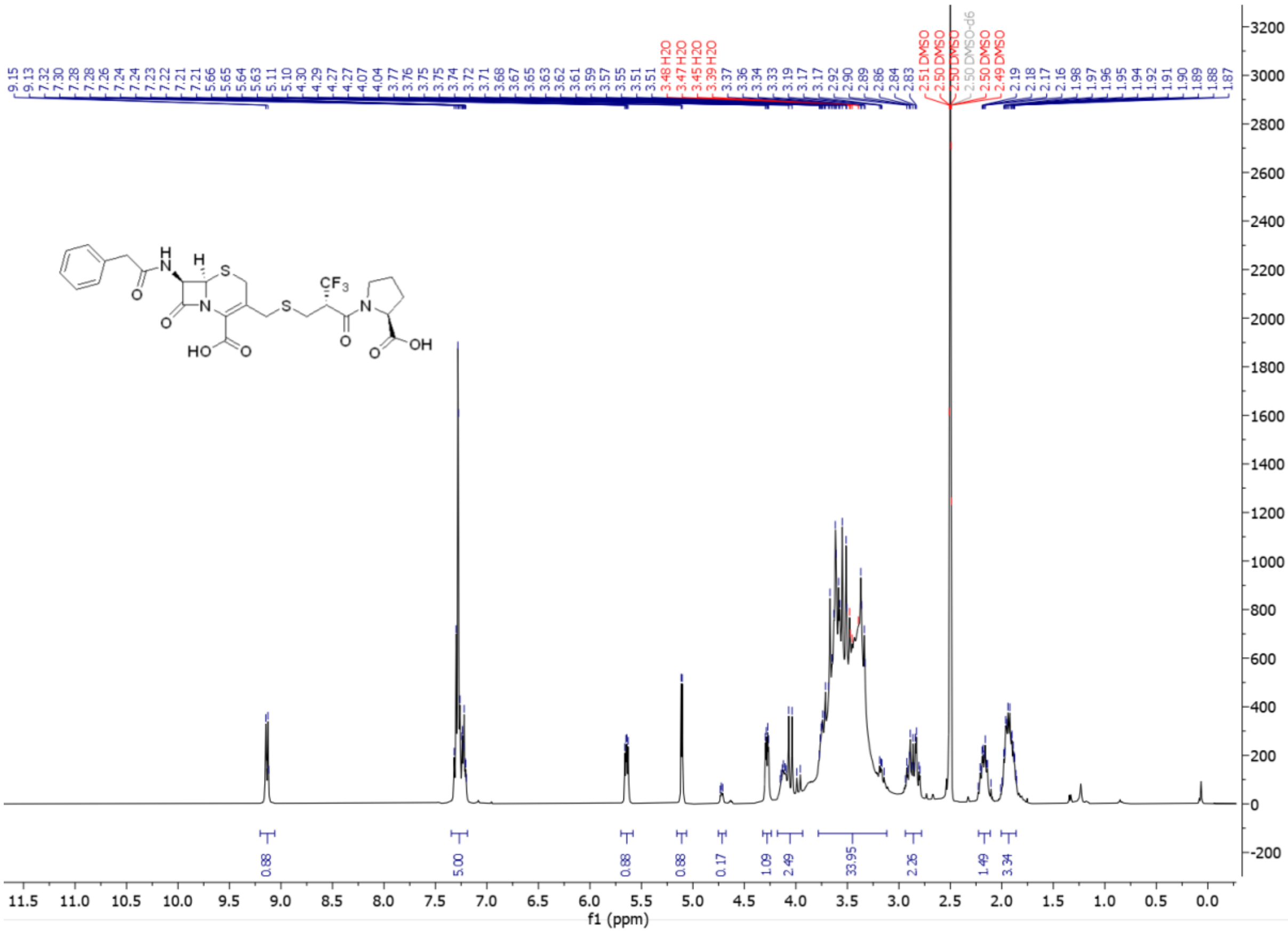

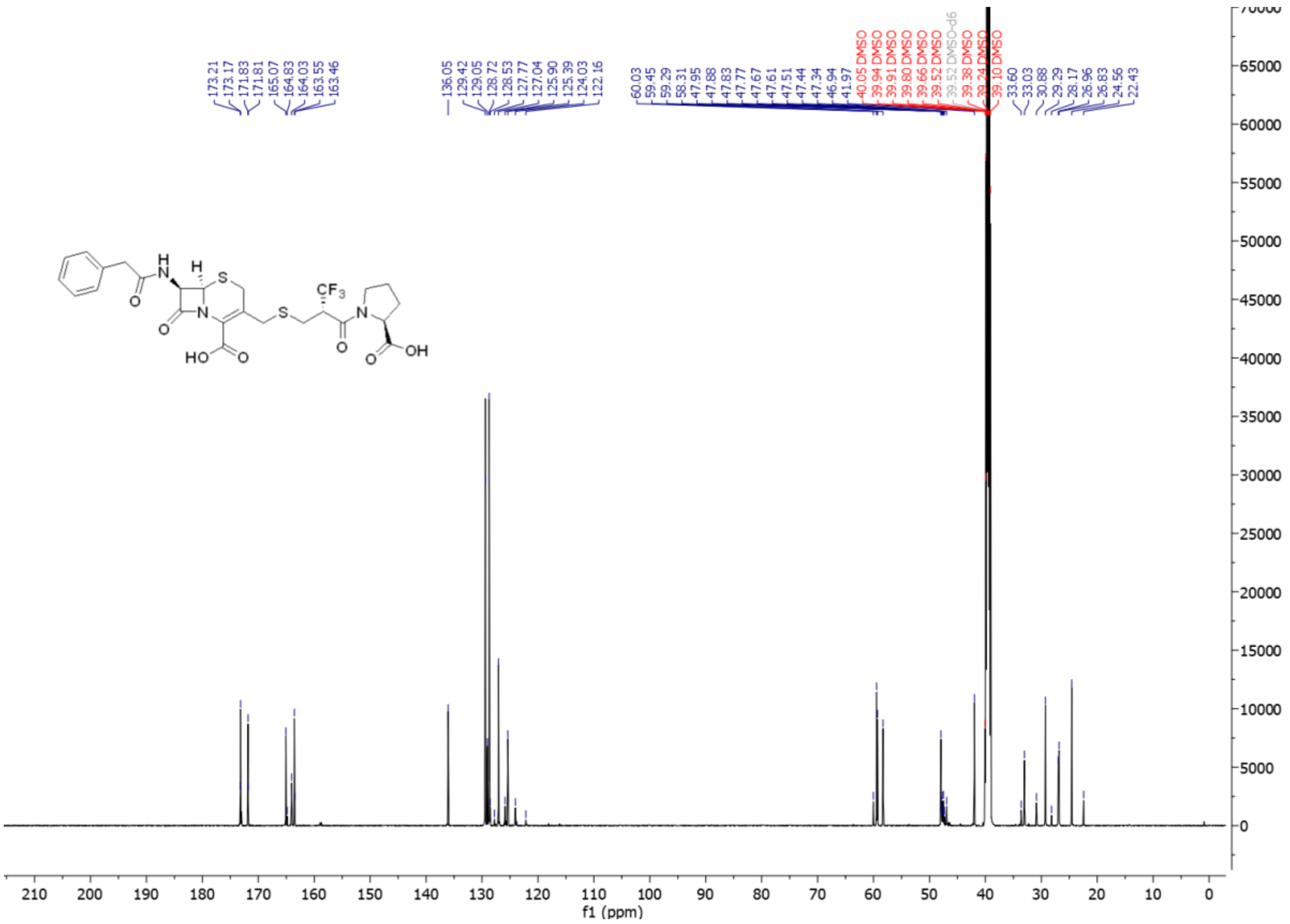

(6R,7R)-3-((((R)-2-((R)-2-carboxypyrrolidine-1-carbonyl)-3,3-trifluoropropyl)thio)methyl)-8-oxo-7-(2-phenylacetamido)-5-thia-1-azabicyclo[4.2.0]oct-2-ene-2-carboxylic acid **(8)**

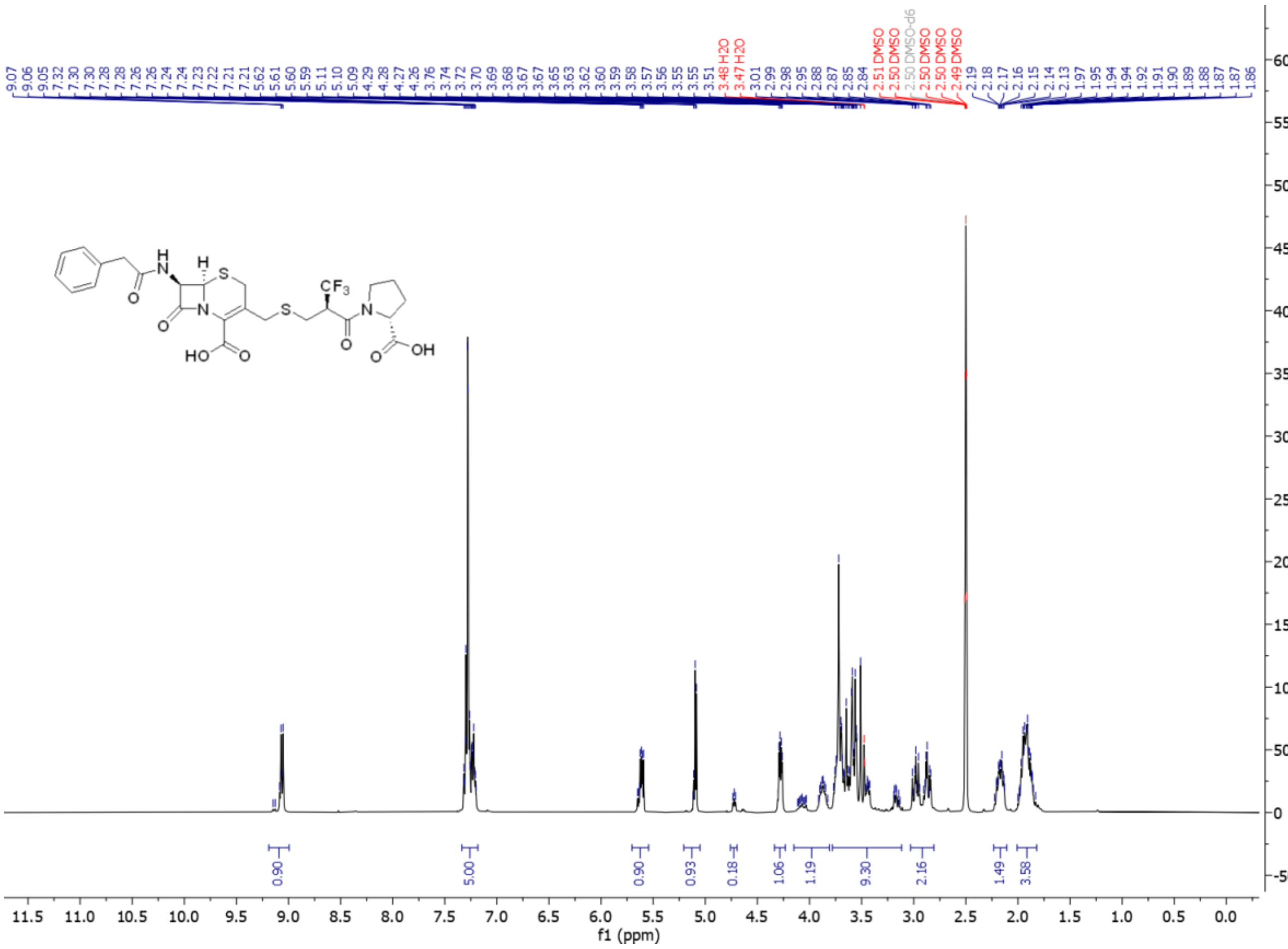

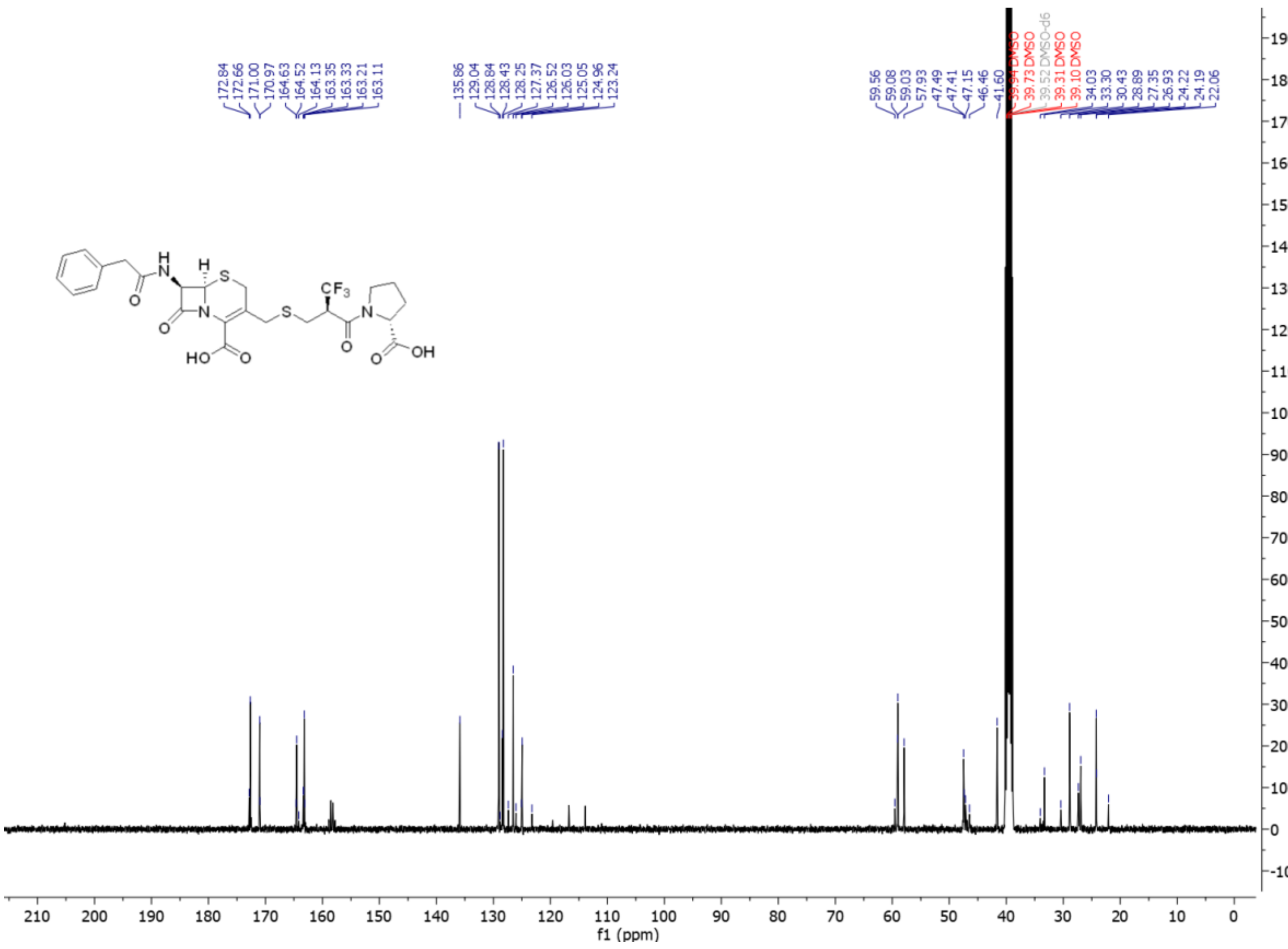

References

(1) Kondratieva, A.; Palica, K.; Frøhlich, C.; Hovd, R. R.; Leiros, H.-K. S.; Erdelyi, M.; Bayer, A. Fluorinated Captopril Analogues Inhibit Metallo-β-Lactamases and Facilitate Structure Determination of NDM-1 Binding Pose. *Eur. J. Med. Chem.* **2024**, *266*, 116140. DOI: https://doi.org/10.1016/j.ejmech.2024.116140.

(2) van Haren, M. J.; Tehrani, K. H. M. E.; Kotsogianni, I.; Wade, N.; Bruchle, N. C.; Mashayekhi, V.; Martin, N. I. Cephalosporin Prodrug Inhibitors Overcome Metallo-β-Lactamase Driven Antibiotic Resistance. *Chemistry* **2021**, *27* (11), 3806-3811. DOI: 10.1002/chem.202004694 From NLM Medline.

(3) Keltjens, R.; Vadivel, Subramanian K.; de Vroom, E.; Klunder, Antonius J. H.; Zwanenburg, B. A New Convenient Synthesis of 3-Carboxycephems Starting from 7-Aminocephalosporanic Acid (7-ACA). *Eur. J. Org. Chem.* **2001**, *2001* (13), 2529-2534. DOI: https://doi.org/10.1002/1099-0690(200107)2001:13<2529::AID-EJOC2529>3.0.CO;2-9 (acccessed 2024/01/15).

(4) Tehrani, K. H. M. E.; Wade, N.; Mashayekhi, V.; Bruchle, N. C.; Jespers, W.; Voskuil, K.; Pesce, D.; van Haren, M. J.; van Westen, G. J. P.; Martin, N. I. Novel Cephalosporin Conjugates Display Potent and Selective Inhibition of Imipenemase-Type Metallo-β-Lactamases. *J. Med. Chem.* **2021**, *64* (13), 9141-9151. DOI: 10.1021/acs.jmedchem.1c00362 From NLM Medline.

(5) Christopeit, T.; Leiros, H.-K. S. Fragment-based discovery of inhibitor scaffolds targeting the metallo-β-lactamases NDM-1 and VIM-2. *Bioorg. Med. Chem. Lett.* **2016**, *26* (8), 1973-1977. DOI: http://dx.doi.org/10.1016/j.bmcl.2016.03.004.
